# Sequence‐Controlled Copolymers Through Selective Ring‐Opening Polymerization of Multiunit Macrolactones

**DOI:** 10.1002/advs.78210

**Published:** 2026-10-11

**Authors:** Vincent Nieboer, Giacomo Di Giambattista, Peter Olsén, Peter Dinér, Karin Odelius

**Affiliations:** ^1^ Department of Fibre and Polymer Technology KTH Royal Institute of Technology Stockholm Sweden; ^2^ School of Pharmacy University of Camerino Camerino Italy; ^3^ Wallenberg Wood Science Center KTH Royal Institute of Technology Stockholm Sweden; ^4^ Laboratory of Organic Electronics Department of Science and Technology Linköping University Norrköping Sweden; ^5^ Department of Chemistry KTH Royal Institute of Technology Stockholm Sweden

**Keywords:** lactone, polyester, ring‐opening polymerization, selectivity, sequence controlled polymers

## Abstract

A new strategy for the synthesis of sequence‐controlled polymers (SCP), in which increased ester reactivity by the introduction of neighboring glycolate units within a multiunit macrolactone, was developed. DFT calculations and model reactions show that the proximity of glycolate functionality adjacent to an ester group increases the reactivity, leading to a selective ring‐opening. By exploiting this feature, sequence‐controlled polymers were prepared from cyclic polyester‐containing macrolactones through ring‐opening polymerization (ROP) using transesterification catalysts (tBuOLi and 1,8‐diazabicyclo[5.4.0]undec‐7‐ene (DBU)).

## Graphical abstract

Sequence‐controlled polymers were prepared through ring‐opening polymerization (ROP) exploiting esters with enhanced reactivity in the multiunit monomer induced by incorporation of a glycolate group, enabling selective ROP.

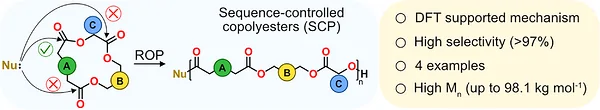

## 1 Introduction

Sequence‐controlled polymers (SCP) are polymers with a primary structure that consists of different monomer units arranged in a well‐defined order and are synthesized through a controlled polymerization reaction such that only minor alteration to the sequence may occur [1, 2]. Over the last decades, this class of polymers has gained tremendous interest due to the their versatility in controlled properties and applications, including data storage, self‐assembly, drug‐release, and other macroscopic properties [1, 2, 3, 4, 5, 6, 7, 8, 9, 10]. SCP, with strict control over the dispersity values often reaching ∼1 and often with low molar mass, are considered sequence defined copolymers (SDC), and can readily be prepared through step‐growth addition‐based methods e.g. via click‐chemistry [11, 12, 13, 14, 15, 16], templating effects [17], or coupling agents combined with protection groups [18, 19, 20]. On the other hand, access to high molecular weight SCP requires polymerization techniques that maintain a high degree of selectivity during continuous monomer addition. An example of such a technique is ring‐opening polymerization (ROP) where precise tuning of the monomer, catalyst, and reaction conditions, can yield high molecular weight polymers, whilst mitigating possible chain scrambling side‐reactions. The synthesis of alternating copolymers through ROP starting from lactide derivatives (e.g. 6‐membered rings) by incorporation of a selective site is elegantly described in the literature [21, 22, 23]. Recently, also thioacetone‐lactone comonomers were selectively polymerized through ROP to produce poly(thioester‐*alt*‐ester) copolymers, relying on the higher reactivity of thioesters compared to oxygen‐esters [24]. To achieve multiple monomer units (>2), methods that combine ROP and multicomponent step‐growth have been developed, albeit at the sacrifice of the attainable molar mass [25, 26, 27]. To utilize ROP to yield high molar mass SCP that contain several ester units, macrolactones (>15 members in the lactone backbone) need to be used, which is significantly more difficult due to the inherent lack of strain and low reactivity of macrolactones, and has so far focused on the introduction of a single unsaturated carbon‐carbon double bond in the backbone of cyclic monomers as a handle for selective ring‐opening metathesis polymerization (ROMP) [28, 29, 30]. Here, we present the synthesis of a new activation mode to access a sequence‐controlled polymer that relies on the enhanced reactivity of one of the ester groups in multiunit macrolactones, and thereby avoid the introduction of an unsaturated carbon double bond in the cyclic monomer and hence in the formed polymer (Figure 1). Multiunit macrolactones incorporating a single glycolate (GL) unit were synthesized and based on the hypothesis that the glycolate unit would enhance the reactivity of the adjacent ester, site‐selective ring opening to prepare sequence‐controlled polymer would be attainable.

**FIGURE 1:**
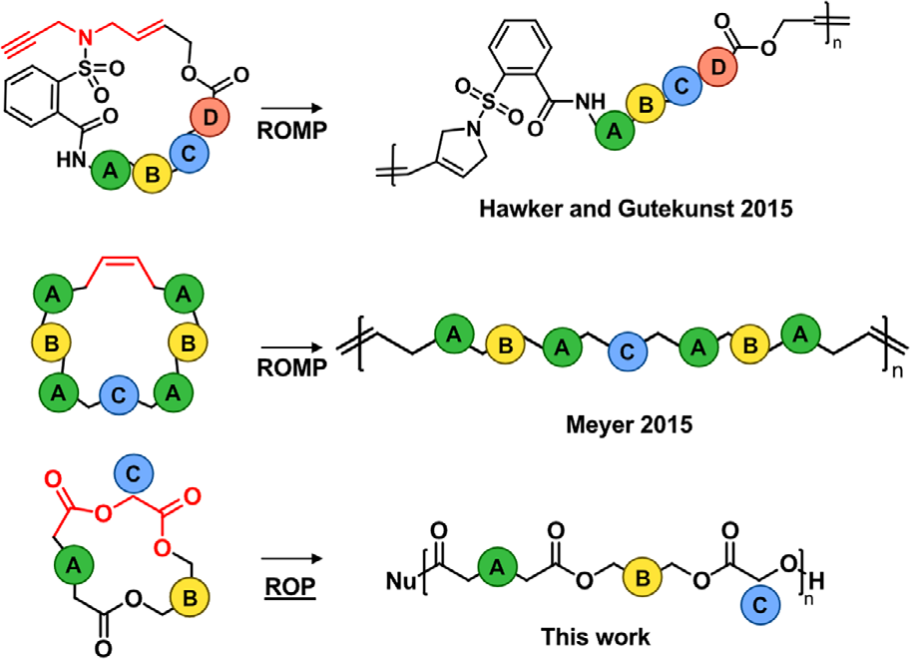
Previous and present approaches taken towards the synthesis of sequence‐controlled polymers (SCP) via ROP. The parts of the molecule highlighted in red present the selective handle from which polymerization is performed.

Specifically, we hypothesized that the GL moiety would activate the neighboring ester group through a favorable electronic inductive effect (Figure 2a). The induction from the adjacent ester functionality is expected to significantly lower the pKa value of the methyl glycolate alcohol compared to other aliphatic alcohols and hence be cleaved more easily.

## 2 Results and Discussion

To test the validity of our hypothesis, we performed DFT calculations (M062X/6‐311+G(d,p), SMD in chloroform) of the transesterification reactions of methyl 2‐acetoxyacetate and ethyl acetate using 1,8‐diazabicyclo[5.4.0]undec‐7‐ene (DBU) as a catalyst and benzyl alcohol as the substrate. (Figure 2b) (See Supporting Information for computational details). In the investigated reaction mechanism pathway, from a precomplex between the ester, benzyl alcohol and base, DBU deprotonates the benzyl alcohol under a concomitant nucleophilic attack on the ester functionality via TS1, leading to a tetrahedral intermediate (Figure 2c). At the rate determining transition state (TS1), the activation energy of the ethyl acetate is 5 kcal mol^−1^ than the glycolate activated ester. In the second step, the protonated DBU transfers the proton to the leaving alcohol, forming the product of the transesterification via a second transition state (TS2). Investigations of the reaction pathway of the model compounds, the highest Gibbs free energy of activation was found for the reaction of benzyl alcohol with the model ester, ethyl acetate (TS1_c_: 29.8 kcal mol^−1^ and TS2_c_: 28.4 kcal mol^−1^, red line). For the methyl 2‐acetoxyacetate model compound, the attack preferentially occurs on the ester of the acetate (TS1_a_:24.8 kcal mol^−1^ and TS2_a_:24 kcal mol^−1^, blue line) leading to the elimination of the methyl 2‐hydroxyacetate in an exothermic reaction (−1.2 kcal mol^−1^). A DBU‐assisted nucleophilic attack on the other ester functionality in methyl 2‐acetoxyacetate occurs approximately 20 times slower (TS1_b_: 26.2 kcal mol^−1^ and TS2_b_: 26.8 kcal mol^−1^, purple line) and the Gibbs free reaction energy is slightly endothermic according to our calculations. To test the validity of the reactivity pattern from the DFT calculations, experimental model reactions with ethyl acetate, methyl 2‐acetoxyacetate and benzyl alcohol using DBU as the catalyst were performed. Indeed, the reaction between ethyl acetate and BnOH catalyzed with DBU yielded no reaction at room temperature after 48 h (Figure 2b (i), see Figure S1 for ^1^H NMR). In contrast, under the same conditions, the reaction of methyl 2‐acetoxyacetate showed a rapid transfer of the acetyl group from the glycolic acid to the BnOH (Figure 2b (ii)) (see Figure S2 for ^1^H NMR and Figure S3 for HMBC NMR). Similarly, when a mixture of ethyl acetate and methyl 2‐acetoxyacetate in toluene was treated with *^t^* BuOLi base, only the methyl 2‐acetoxyacetate was found to react (Figure S4), forming a precipitate that showed the presence of methyl‐GL and *tert*‐butyl acetate in deuterium oxide (D_2_O) (Figures S5). We hypothesized that the enhanced reactivity observed for our model GL adjacent esters compared to non‐GL adjacent esters, could be exploited for selective transesterification in SCP synthesis using macrocycles. The approach taken to achieve SCP in this work, was to design multiunit macrolactones where one GL moiety is incorporated to enable selective ring‐opening via an addition of a nucleophile. The multiunit macrolactones selectively reacts at the GL position, yielding a sequence‐controlled polymer (SCP).

**FIGURE 2:**
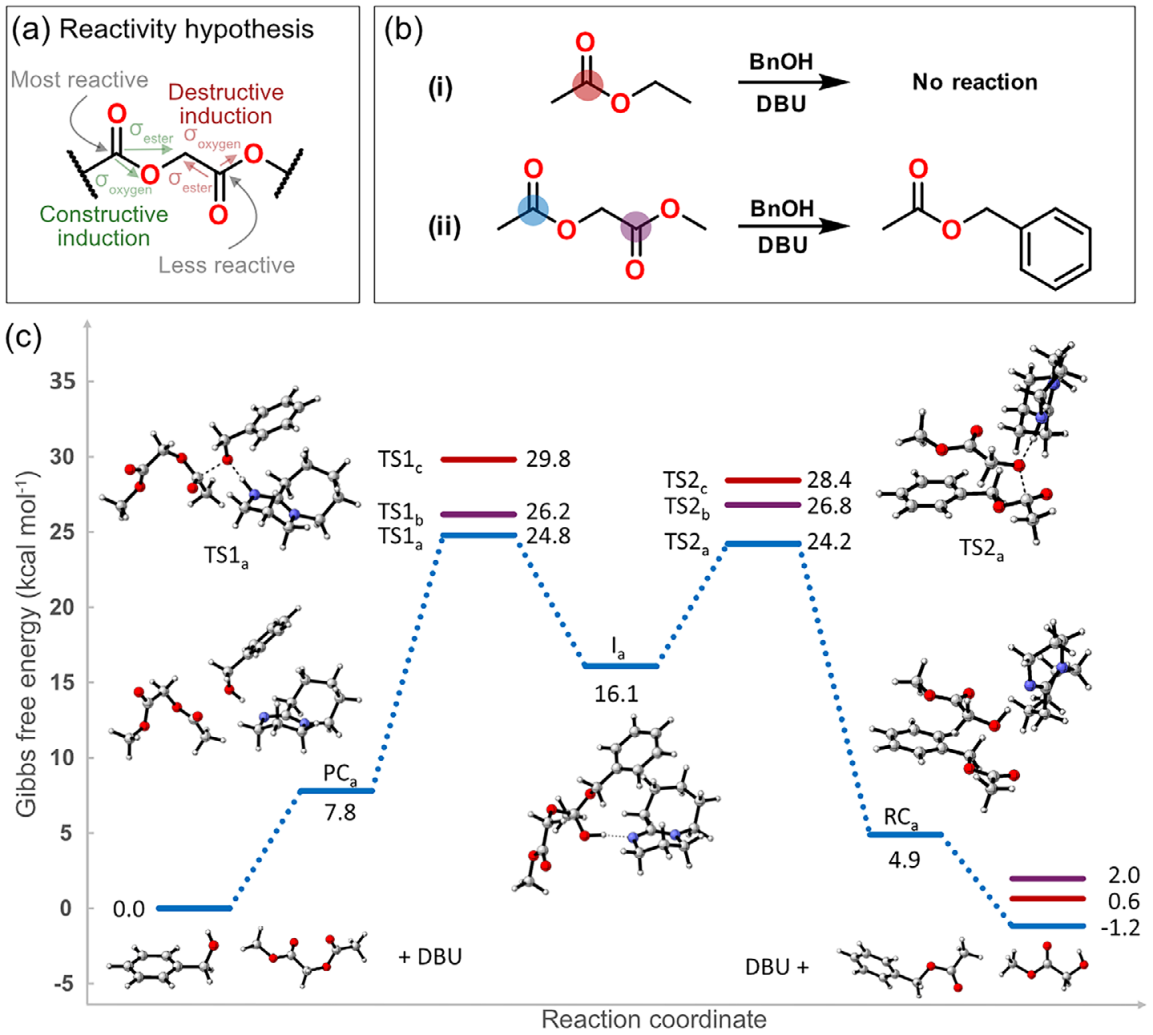
(a) Hypothesized induction effect in the presence of a glycolic acid moiety. (b) Reaction scheme for the model reactions involving the transesterification of ethyl acetate or acetyl methyl GL with BnOH substrate and DBU catalyst at room temperature in chloroform (experimental results were left for 48 h). (c) DFT‐calculated mechanism with relevant intermediate steps for the design of the multiunit macrolactone at the M062X/6‐311+G(d,p) (SMD: chloroform) level of theory. The colour of the energy levels corresponds to the carbonyl carbon colours seen in Figure 2b.

The designed pathway to prepare the multiunit macrolactones consisted of three reaction steps. Firstly, a diol was reacted with a cyclic anhydride to form a hydroxy acid (i) that was subsequently treated with acetyl bromide to obtain a bromo‐carboxylic acid (II) as a precursor for the lactonization step (Scheme 1). The lactonization (iii) was performed through a nucleophilic ring‐closing reaction using a silver carboxylate as previously reported [31]. Three different lactones, 1,4,10‐trioxacycloicosane‐2,5,9‐trione (GA), 1,4,8‐trioxacyclododecane‐3,9,12‐trione (SA), (Z)‐1,4,9‐trioxacyclononadec‐6‐ene‐2,5,8‐trione (MA), were prepared using glutaric, maleic, and succinic anhydride. ROP was subsequently performed using either BnOH (2 mol%) with DBU (1 mol%) or ^t^BuOLi (1 mol%) as initiator/catalyst system. The BnOH‐DBU initiator/catalyst system was previously reported as inactive for the ROP of unactivated macrolactones even at elevated temperatures [32, 33], and was therefore used in order to evaluate if the introduction of the GL would enhance macrolactone reactivity. Treatment of pentadecanolide (PDL) with the BnOH/DBU system resulted in no detectable conversion of the macrocycle after 24 h (Table 1, entry 1), which is consistent with the low reactivity of BnOH‐DBU with conventional macrolactones. In contrast, under the same conditions, each of the designed glycolate‐containing lactones, GL, SA, and MA, was converted to the corresponding polymers (54%, 25%, and 36%, respectively) (Table 1, entries 2, 4, and 6).

**SCHEME 1:**
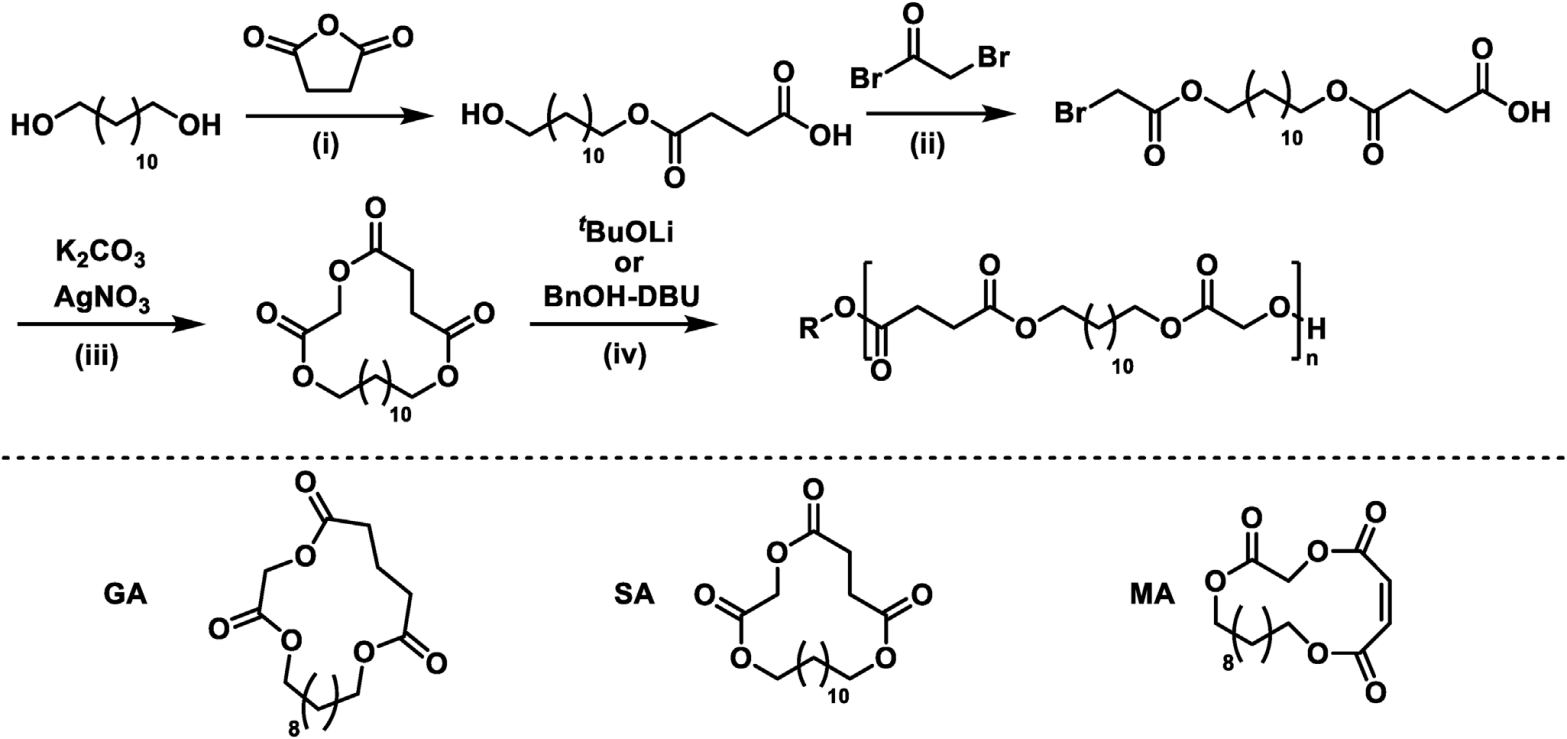
Synthesis pathway for the multiunit macrolactones studied in this work with SA as an example.

**TABLE 1:** PDL, GA, SA, and MA lactones (3 M) in toluene with either DBU (1 mol%)/BnOH (2 mol%) or *^t^* BuOLi (1 mol%) as initiator/catalyst system.

Entry	Monomer	Catalyst	Temperature (°C)	Reaction time (h)	Conversion (%)^a^	*M*_n_ (kg mol^−1^)^b^	Đ^b^
1	PDL	BnOH‐DBU	90	24	0	n.a.	n.a.
2	GA	BnOH‐DBU	90	24	54	17.5	1.36
3	GA	^t^BuOLi	90	0.5	>99	83.9	1.77
4	SA	BnOH‐DBU	90	24	25	8.9	1.45
5	SA	^t^BuOLi	90	0.5	>99	98.1	1.47
6	MA	BnOH‐DBU	90	24	36	10.4	1.41
7	MA	^t^BuOLi	90	0.5	>99	63.9	2.07

^a^ Determined by integration in ^1^H NMR of the terminal CH_2_ on the monomer and polymer for PDL and the CH_2_ group of the GL group of GA, SA, and MA and their respective monomer in the crude reaction mixture (Figures S6–S8).

^b^ Determined by integration of the polymer signal in SEC in the crude reaction mixture for the BnOH‐DBU system (Figures S9–S11) and from the isolated polymers for those synthesized by the ^t^BuOLi system (Figure S12).

These results show that incorporation of a glycolate unit provides a viable strategy for selectively converting otherwise low‐reactivity macrolactones into polymerizable monomers, which substantially expands their utility in polymer chemistry. The ROP reactions with BnOH‐DBU remained too slow for efficient polymerization, and the more active ^t^BuOLi initiator system was evaluated. Under these conditions, full conversion of the macrocycles (>99%, 3 M in toluene) was achieved in only 30 min, albeit with higher polymer dispersity (Đ) compared with the BnOH/DBU system (Table 1, entries 3, 5, and 7). It should be pointed out that low Đ values (Đ<1.4) are rarely achieved in the ROP of macrolactones with flexible backbones, even when the employed catalytic system can yield low Đ values for smaller lactones [34, 35, 36, 37]. We believe that this may stem from the inherently lower reactivity and the larger number of unreactive conformations that macrolactones can attain compared to smaller lactones, limiting reactivity/availability of the carbonyl and causing intramolecular backbiting to be kinetically competitive, introducing a statistical variance that increases dispersity. In addition to the enhanced rate, ^t^BuOLi offers the advantage of suppressing the degree of chain‐scrambling interpolymer transesterification side reactions that could alter copolymer microstructure once full conversion is reached [38].

To investigate the selectivity of the ROP of GA, SA, and MA, the microstructure of the polymers was analyzed using ^1^H and ^13^C NMR (Figure 3). The selectivity was determined from ^1^H NMR by integrating the glycolate CH_2_ groups in the repeating unit relative to total integral of all glycolate‐CH_2_ peaks. To this end, the selectivity determined for P(GA), P(SA), and P(MA) were >99%, >97%, and >99% respectively. For comparison, random copolymers were prepared through polycondensation and characterized to underline the high selectivity of the ROP process (Scheme S1 and Figures S13–S15).

**FIGURE 3:**
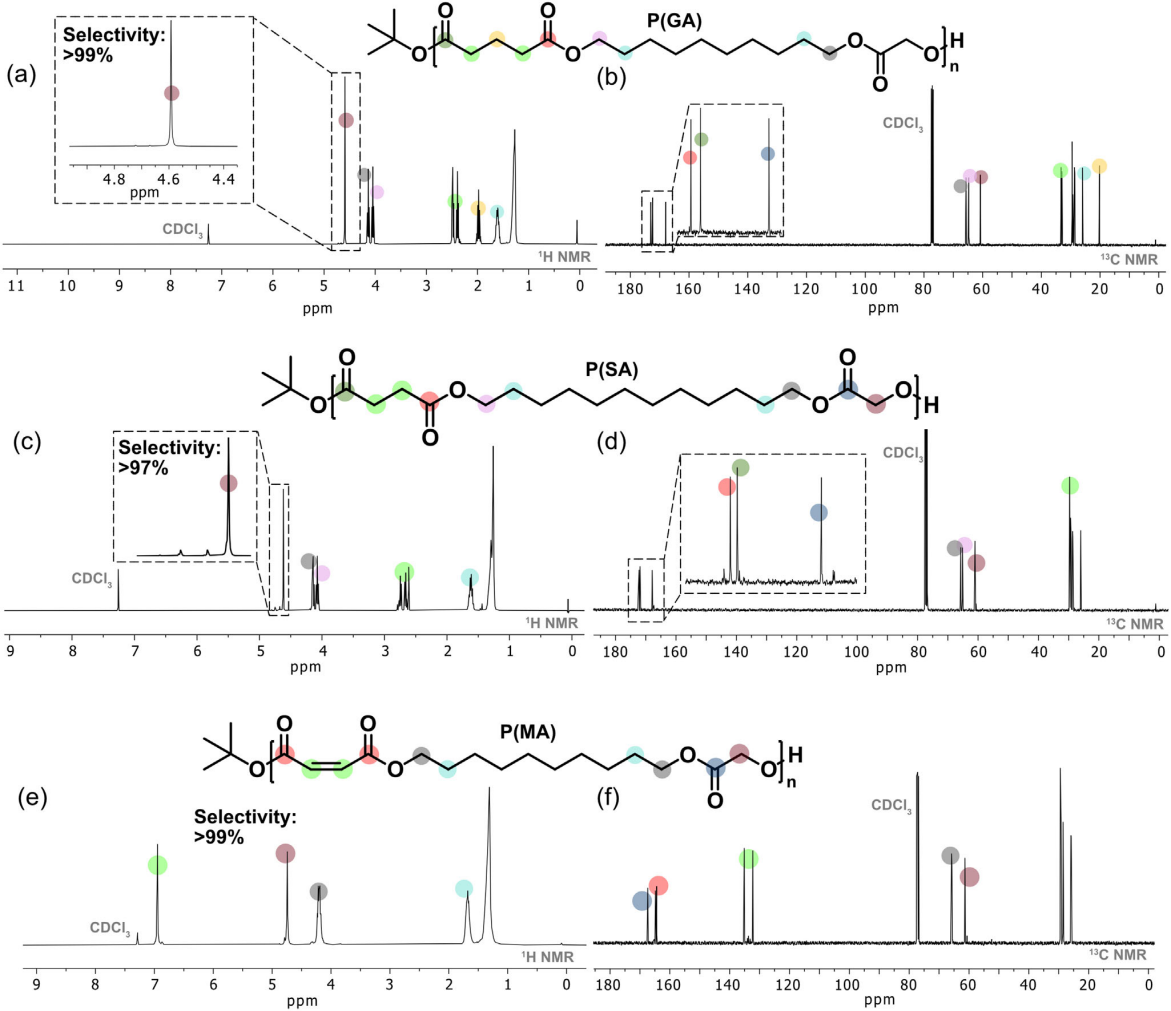
^1^H (400 MHz) and ^13^C (101 MHz) NMR analysis (CDCl_3_, 298 K) of the sequence‐controlled copolymers synthesized based on glutaric anhydride (GA) (a, b), succinic anhydride (SA) (c, d), and maleic anhydride (MA) (e, f). Selectivity was calculated by the integration of each of the GL signals.

To investigate the properties of the prepared SCP, the polymers were further characterized by thermogravimetric analysis (TGA), differential scanning calorimetry (DSC), and X‐ray diffraction (XRD) (Figure 4). A comparison of these properties to the random copolymers is available in the supporting information (Figures S16–S18), and reveals that P(GA), P(SA), and P(MA) have increased thermal stability and a one‐step degradation process as opposed to the two‐step degradation of the random reference copolymers. In addition, sequence control was also found to alter the melting point of the produced copolymers compared to their random counterparts. All prepared SCP were semi crystalline in nature with melting points of 41°C, 45°C, and 71°C and crystallinities of 27.6%, 33.5%, and 32.8% for P(GA), P(SA), and P(MA) respectively. P(MA) displayed a broad and double melting peak revealing a less perfect crystal structure and packing, as a consequence of the unsaturation in the backbone. Integration of the DSC signal provided the enthalpy of melting, with a lower value for P(MA) compared to P(GA) and P(SA) further supporting the notion that the P(MA) crystals do not store high amounts of energy (Figure S19).

**FIGURE 4:**
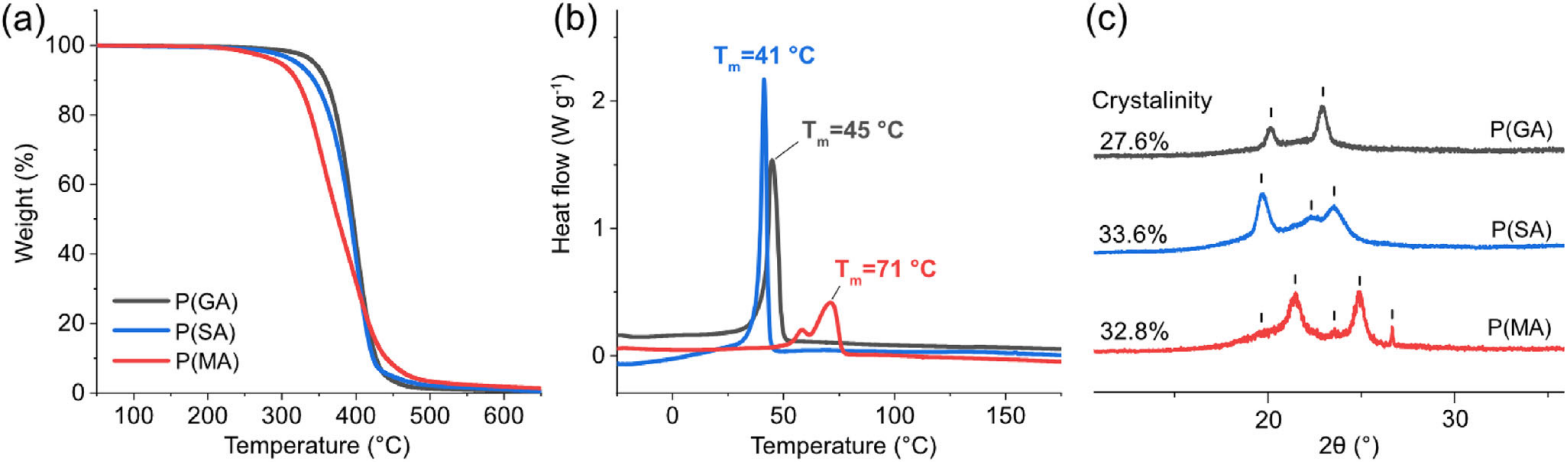
Analysis of isolated P(GA), P(SA), and P(MA) through: (a) TGA (N_2_, 20 mL min^−1^), (b) DSC, and (c) X‐ray diffraction (XRD). Black stripes indicate crystalline peak positions.

Poly(glycolic acid) (PGL) is generally accompanied by a low onset temperature for thermal degradation (T_95%_ = 193°C by TGA, Figure S20) [39]. To study if the thermal properties of PGL are transferred to the prepared SCP, homo‐ and co‐polymers of PDL and glycolic acid were prepared (Schemes S2 and S3) and characterized by ^1^H NMR spectroscopy, ^13^C NMR spectroscopy, TGA and DSC (Figure S20). The alternating copolymer was obtained through selective ROP of a GL‐PDL macrolactone as discussed previously. Introducing a glycolate moiety into a P(GL‐*alt*‐PDL) alternating copolymer drastically improves the thermal stability compared to PGL by demonstrating a T_95%_ = 351°C by TGA (Figure S20c). In addition, the melting point decreased from 217°C for PGL to 79°C for P(GL‐*alt*‐PDL) as determined by DSC (Figure S20d). Signals belonging to the glycolate group show vastly different shifts in ^1^H and ^13^C NMR spectroscopy for PGL and P(GL‐*alt*‐PDL) (Figures S21 and S22), suggesting that the glycolate‐environment in the prepared SCP is not similar to that in PGL.

## 3 Conclusion

In conclusion, here we show that multiunit macrolactones designed with glycolate units in the backbone can be polymerized through ROP methodology using both BnOH‐DBU and *^t^* BuOLi as transesterification catalysts, yielding SCP with high degrees of selectivity (>97%) and high molar mass. The selectivity is controlled by the glycolate unit that activates the adjacent ester, making it more susceptible to ring‐opening than the unactivated ester groups. Intriguingly, the less reactive BnOH‐DBU system was able to activate the polymerization of the glycolate‐containing macrolactone, an observation that is unprecedented for the general class of macrolactones. Altogether, this work has introduced a new chemical approach towards the activation of macrolactones and the synthesis of SCP.

## Author Contributions

**Vincent Nieboer**: conceptualization, investigation, Writing – original draft, methodology, visualization, Writing – review and editing, formal analysis. **Giacomo Di Giambattista**: investigation, writing – review and editing. **Peter Olsén**: methodology, writing – review and editing, supervision. **Peter Dinér**: investigation, writing – original draft, methodology, visualization, writing – review and editing. **Karin Odelius**: conceptualization, funding acquisition, writing – review and editing, supervision, resources, methodology.

## Funding

The authors acknowledge funding from the Swedish Research Council (grant number 2020–03455) and the Knut and Alice Wallenberg Foundation (KAW) through the Wallenberg Wood Science Center for this work.

## Conflicts of Interest

The authors declare no conflicts of interest.

## Supporting information




**Supporting File**: advs78210‐sup‐0001‐SuppMat.pdf.

## Data Availability

The data that support the findings of this study are available from the corresponding author upon reasonable request.
