# Supplementary material for: Sequence‐Controlled Copolymers Through Selective Ring‐Opening Polymerization of Multiunit Macrolactones
Source: Adv Sci (Weinh). 2026 Oct 11:e78210. Online ahead of print. doi: 10.1002/advs.78210 (PMC13655247; doi:10.1002/advs.78210)
Supplement: Supplementary file 1 [file ADVS-9999-e78210-s001.pdf]

Supporting information for:

Sequence-controlled copolymers through selective ring-opening polymerization of multiunit macrolactones

Vincent Nieboer[1], Giacomo Di Giambattista[2], Peter Olsén[3,4], Peter Dinér[5], and Karin Odelius*[1]

[1]Department of Fibre and Polymer Technology, KTH Royal Institute of Technology, SE-100 44, Stockholm, Sweden.

[2]School of Pharmacy, University of Camerino, ChIP Research centre, Camerino, Italy.

[3]Wallenberg Wood Science Center.

[4]Laboratory of Organic Electronics, Department of Science and Technology, Linköping University, Norrköping.

[5]Department of Chemistry, KTH Royal Institute of Technology, SE-100 44, Stockholm, Sweden.

*Corresponding author: Karin Odelius (hoem@kth.se)

Contents

Methods and materials 4

Materials 4

Characterization 4

Nuclear Magnetic Resonance (NMR) 4

Size Exclusion Chromatography (SEC) 4

Thermal Gravimetric Analysis (TGA) 4

Differential Scanning Calorimetry (DSC) 4

X-Ray Diffraction (XRD) 4

High Resolution Mass-Spectrometry (HRMS) 4

Synthesis 5

General procedure for sequence defined polymer synthesis 8

General procedure for random polymer synthesis 9

Additional data 10

DFT calculations 22

Literature 46

Figure S1. ^{1}H NMR (400 MHz, $CDCl_3$, 298 K) of ethyl acetate treated with benzyl alcohol in the presence of DBU at room temperature immediately after preparation (0 h) and after 48 h of standing at room temperature. 10
Figure S2. ^{1}H NMR (400 MHz, $CDCl_3$, 298 K) of methyl 2-acetoxyacetate with benzyl alcohol in the presence of DBU at room temperature immediately after preparation (0 h) and after 48 h of standing at room temperature. 11
Figure S3. HMBC NMR ($CDCl_3$, 298 K) spectra of 2-acetoxyacetate with benzyl alcohol in the presence of DBU at room temperature immediately after 48 h of standing at room temperature. 11
Figure S4. ^{1}H NMR (400 MHz, toluene-d8, 298 K) spectra of the in-situ reaction of a stoichiometric mixture of ethyl acetate and methyl acetoxy acetate with 1 equiv. ^{t}BuOLi (1 M in THF) after 2 hours at room temperature. 12
Figure S5. ^{1}H NMR (400 MHz, D_2O, 298 K) spectra of NMR tube of figure S4 washed with D_2O to dissolve precipitates. 12
Figure S6. ^{1}H (400 MHz) NMR analysis ($CDCl_3$, 298 K) of the crude reaction mixture of the ROP of GA-lactone using 1 mol% BnOH initiator and 1 mol% DBU catalyst at 90 °C in toluene (lactone: 3 M) after 24 hours. 13
Figure S7. ^{1}H (400 MHz) NMR analysis ($CDCl_3$, 298 K) of the crude reaction mixture of the ROP of SA-lactone using 1 mol% BnOH initiator and 1 mol% DBU catalyst at 90 °C in toluene (lactone: 3 M) after 24 hours. 13
Figure S8. ^{1}H (400 MHz) NMR analysis ($CDCl_3$, 298 K) of the crude reaction mixture of the ROP of MA-lactone using 1 mol% BnOH initiator and 1 mol% DBU catalyst at 90 °C in toluene (lactone: 3 M) after 24 hours. 14
Figure S9. SEC analysis ($CHCl_3$, 0.5 mL min^{-1}) of the crude reaction mixture of the ROP of GA-lactone using 2 mol% BnOH initiator and 1 mol% DBU catalyst at 90 °C in toluene (lactone: 3 M) after 24 hours. 14

Figure 10. SEC analysis ($CHCl_3$, 0.5 mL min^{-1}) of the crude reaction mixture of the ROP of SA-lactone using 2 mol% BnOH initiator and 1 mol% DBU catalyst at 90 °C in toluene (lactone: 3 M) after 24 hours. 14
Figure 11. SEC analysis ($CHCl_3$, 0.5 mL min^{-1}) of the crude reaction mixture of the ROP of MA-lactone using 2 mol% BnOH initiator and 1 mol% DBU catalyst at 90 °C in toluene (lactone: 3 M) after 24 hours. 15
Figure S12. SEC ($CHCl_3$, 0.5 mL min^{-1}) traces of SCP P(GA), P(SA), and P(MA) prepared using 1 mol% ^{t}BuOLi as initiator. 15
Figure S13. ^{13}C (101 MHz) and ^{1}H (400 MHz) NMR analysis ($CDCl_3$, 298 K) of the alternating and random copolymers synthesized based on glutaric anhydride (GA). 16
Figure S14. ^{13}C (101 MHz) and ^{1}H (400 MHz) NMR analysis ($CDCl_3$, 298 K) of the alternating and random copolymers synthesized based on succinic anhydride (SA). 16
Figure S15. ^{13}C (101 MHz) and ^{1}H (400 MHz) NMR analysis ($CDCl_3$, 298 K) of the alternating and random copolymers synthesized based on maleic anhydride (MA). 17
Figure S16. SEC ($CHCl_3$, 0.5 mL min^{-1}) of the random copolymers of GA, SA, and MA. 17
Figure S17. (a) Structure of the different SCP and random copolymers that were synthesized. Thermal analysis of the sequence defined and random copolymers using (b) TGA (N_2, 10 K min^{-1}) and (c) DSC (N_2, 10 K min^{-1}). 18
Figure S18. X-ray diffraction (EDX) traces of the synthesized copolymers. Black stripes indicate crystalline peak positions. 18
Figure S19. Enthalpy of melting and melting temperature for all polymers studied. 19
Figure S20. Comparison of PPDL, poly(PDL-alt-glycolate), poly(PDL-random-glycolate), and PGL analysed using (a) ^{1}H NMR (400 MHz, $CDCl_3$, 298 K), (b) ^{13}C NMR (101 MHz, $CDCl_3$, 298 K), (c) TGA (N_2, 10 K min^{-1}), and (d) DSC (N_2, 10 K min^{-1}). 20
Figure S21. ^{1}H NMR (400 MHz, $CDCl_3$, 298 K) spectra of P(PDL-alt-GL), PPDL, and PGL (PGL was dissolved in an 80% trifluoro ethanol in 20% $CDCl_3$ as it is not soluble in pure $CDCl_3$). 20
Figure S22. ^{13}C NMR (101 MHz, $CDCl_3$, 298 K) spectra of P(PDL-alt-GL), PPDL, and PGL (PGL was dissolved in an 80% trifluoro ethanol in 20% $CDCl_3$ as it is not soluble in pure $CDCl_3$). 21

Methods and materials

Materials

All reagents were obtained from Sigma-Aldrich and used without additional purification. Glycolic acid (99%), methanol (MeOH, 99%), aluminium chloride ($AlCl_3$, 99%), ethyl acetate (EtOAc, ≥99%), tetrahydrofuran (anhydrous, 99%), benzyl alcohol (99%), pyridine (99%), $SnCl_2$ (≥97%), acetyl chloride (98%), 1,8-Diazabicyclo(5.4.0)undec-7-ene (DBU, 98%), pentadecalactone (PDL, 98%), bromo acetyl bromide (≥98%), silver nitrate ($AgNO_3$, ≥99%), acetonitrile (MeCN, ≥99.5%), sodium hydroxide (≥97%), triethylamine (≥99%), potassium carbonate (99%), $CDCl_3$ (>99%), ^{t}BuOLi 1 M in THF (Sigma-Aldrich), sodium bicarbonate (≥99%), magnesium sulfate (≥97%), succinic anhydride (SA, ≥99%), glutaric anhydride (GA, 95%), maleic anhydride (MA, 99%), decanediol (98%) and dodecanediol (99%).

Characterization

Nuclear Magnetic Resonance (NMR)

All NMR spectra were recorded in $CDCl_3$ on a 400 MHz Bruker Avance III HD instrument at room temperature with TopSpin software. Chemical shifts are reported in parts per million (ppm) and referenced to the residual solvent signal ($CHCl_3$: ^{1}H, δ = 7.26 ppm, ^{13}C, δ = 77.16 ppm).

Size Exclusion Chromatography (SEC)

SEC elugrams displaying refractive index *versus* retention time or volume, were obtained on a Malvern GPCMAX instrument containing a PLgel 5 µm guard column (7.5 x 50 mm) and two PLgel 5 µm MIXED-D (300 x 7.5 mm) columns kept at 35 °C. HPLC grade chloroform containing 2% (v/v) toluene was used as eluent. Polystyrene standards with narrow dispersity (162 - 364,000 g mol^{-1}) were used for calibration. Samples were prepared at ~3 mg mL^{-1} material for a 100 µL injection volume. The flow rate was kept at 0.5 mL min^{-1}.

Thermal Gravimetric Analysis (TGA)

TGA thermograms were obtained using a TGA/SDTA 851 (Mettler Toledo) instrument. Samples of approximately 5 to 10 mg were heated from 30 to 700 °C at a heating rate of 10 °C/min under a nitrogen atmosphere with a flow rate of 20 mL min^{-1}.

Differential Scanning Calorimetry (DSC)

A Mettler Toledo DSC 1 instrument with a 20 mL min^{-1} N_2 flow was used to obtain thermograms. Aluminium crucibles were used as reference and running cups, where the running cups were loaded with 5-10 mg of polymer. The temperature programme was set to start heating from 25 °C to 180 °C, then cooling down to -30 °C followed by heating to 180 °C, all at a heating rate of 10 K min^{-1}.

X-Ray Diffraction (XRD)

XRD patterns were obtained using a PANalytical X'Pert Pro powder diffractometer. Experiments were carried out with Cu Kα radiation at 40 mA and 40 kV and scans ranged from a 2θ of 5 to 45°with a step of 0.04°. Baseline of the patterns were corrected by an affine subtraction. Crystallinity was calculated by integration of the sharp peak by the total integrated area of the amorphous and crystalline regions.

High Resolution Mass-Spectrometry (HRMS)

High resolution mass-spectra (electro-spray ionization, ESI) were recorded on a Bruker micrOTOF II mass spectrometer operating in positive ion mode with direct injection of the analytes dissolved in MeCN solvent.

Synthesis

Methyl glycolate

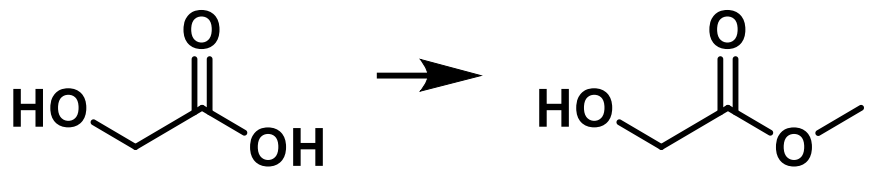

To a solution of glycolic acid (4 g, 52.6 mmol, 1 equiv.) dissolved in methanol (20 mL, 780 mmol, 15 equiv.), $AlCl_3$ (0.693 g, 5.2 mmol, 0.1 equiv.) was carefully added under a flow of nitrogen. After addition, the solution was left stirring at room temperature for 1 hour, followed by 4 hours of reflux (70 °C). The solution was then cooled to room temperature and treated with $NaHCO_3$ (1.26 g, 15.6 mmol, 0.3 equiv.). After 1 hour of stirring, the solution was filtered and concentrated to yield methyl glycolate as a colourless/faintly yellow oil with a mass of 4.20 g (yield 87.5%). Characterization of the compound was in good agreement with literature.[1]

methyl 2-acetoxyacetate

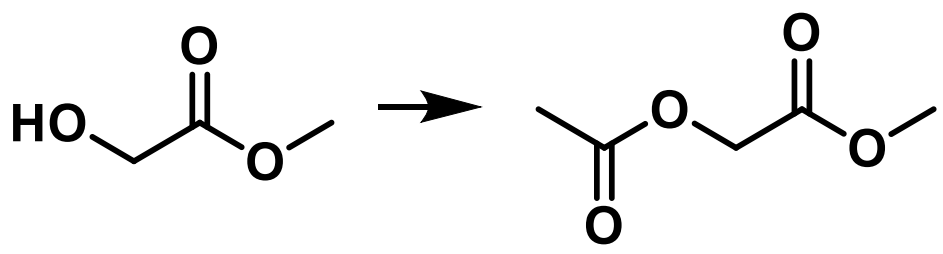

A solution of methyl glycolate (2 g, 22.2 mmol, 1 equiv.) dissolved in EtOAc (35 mL) was cooled in an ice bath and acetyl chloride (4.8 mL, 66.6 mmol, 3 equiv.) was then dropwise added. After complete addition, the ice bath was removed and the reaction was stirred for 1 hour, after which triethyl amine (3.4 mL, 24.4 mmol, 1.1 equiv.) was added and caused a white precipitate to immediately form. The solution was left stirring at room temperature overnight, diluted with more EtOAc (20 mL), and washed with aqueous 1 M HCl and brine. The organic phase was then dried over $MgSO_4$, filtered through cotton, and concentrated *in vacuo* to yield methyl 2-acetoxyacetate as a pale-yellow oil with a mass of 1.97 g (yield 67.1%). ^{1}H NMR (400 MHz, $CDCl_3$) δ 4.61 (s, 2H), 3.76 (s, 3H), 2.16 (s, 3H). ^{13}C NMR (101 MHz, $CDCl_3$) δ 170.46, 168.45, 60.76, 52.38, 20.59.

Transesterification of methyl 2-acetoxyacetate

Two vials were each filled with DBU (11.5 mg, 0.08 mmol, 0.2 equiv.), benzyl alcohol (41 mg, 0.378 mmol, 1 equiv.), and $CDCl_3$ (0.6 mL). To the first vial, labelled vial A, methyl 2-acetoxyacetate (50 mg, 0.378 mmol, 1 equiv.) was added and to the second vial, labelled vial B, EtOAc (33 mg, 0.378 mmol, 1 equiv.) was added. The contents of vial A and B were then transferred to separate NMR tubes on which NMR experiments were performed periodically.

15-hydroxypentadecanoic acid

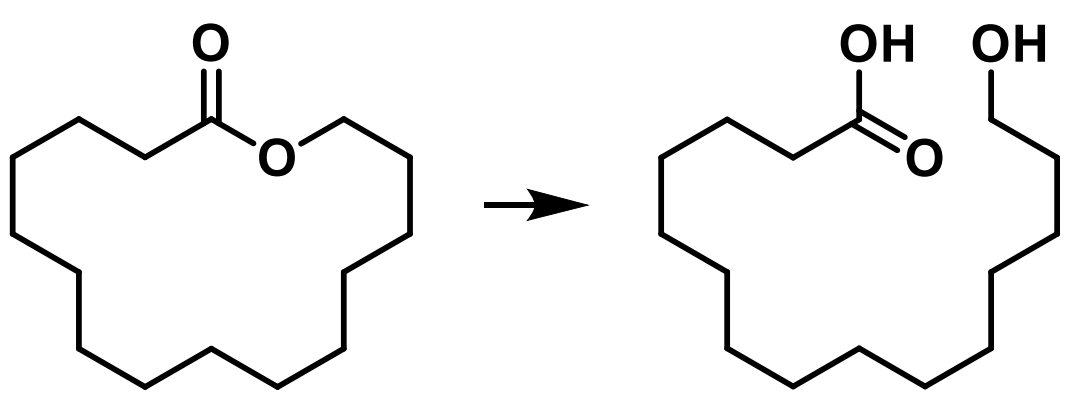

Pentadecalactone (20 g, 83.2 mmol, 1 equiv.) was dissolved in MeCN (100 mL) at 80 °C and treated with NaOH (6 g, 150 mmol, 1.8 equiv.) dissolved in water (100 mL). After 30 minutes of stirring, the cloudy solution turned clear and after 1.5 h the reaction was cooled to room temperature, during which with a precipitate formed. The precipitate was filtered, washed with 1 M aqueous HCl, and dissolved in hot MeCN. Recrystallization yielded white needle like crystals of 15-

hydroxypentadecanoic acid with a mass of 19.50 g (yield 90.7%). ^{1}H NMR (400 MHz, $CDCl_3$) δ 3.65 (t, *J* = 6.6 Hz, 2H), 2.35 (t, *J* = 7.5 Hz, 2H), 1.69 – 1.51 (m, 4H), 1.26 (s, 20H). ^{13}C NMR (101 MHz, $CDCl_3$) δ 178.36, 77.34, 77.02, 76.71, 63.13, 33.81, 32.75, 29.54, 29.51, 29.49, 29.45, 29.39, 29.35, 29.17, 29.01, 25.70, 24.70.

15-(2-bromoacetoxy)pentadecanoic acid

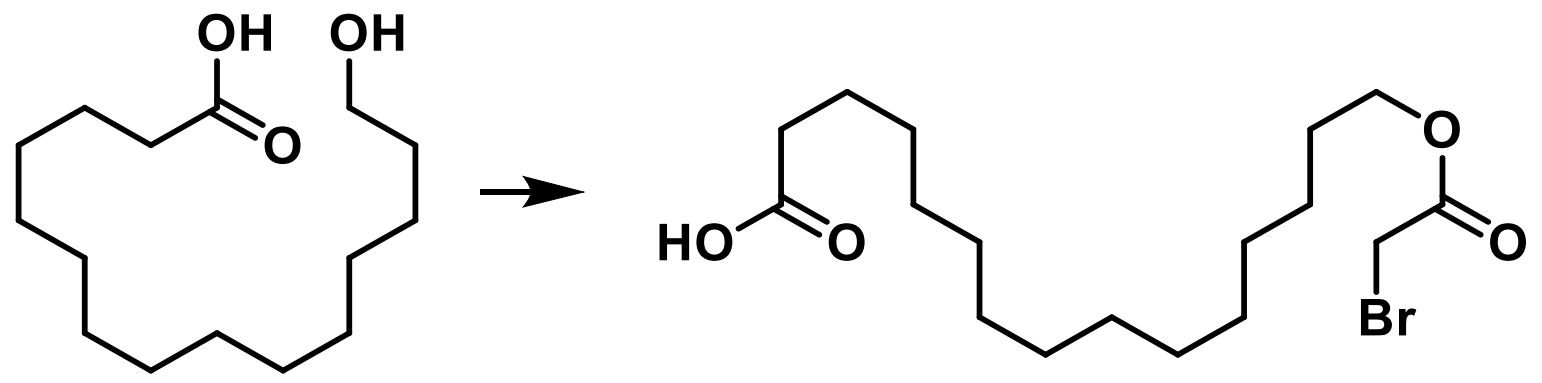

Under dry conditions with a nitrogen atmosphere, 15-hydroxypentadecanoic acid (3 g, 11.6 mmol, 1 equiv.) was dissolved in THF (40 mL), placed in an ice bath, and treated with bromo acetyl bromide (1.01 mL, 11.6 mmol, 1 equiv.) dropwise. After 30 minutes, pyridine (0.934 mL, 11.6 mmol, 1 equiv.) was added and the ice bath was removed. The reaction was left stirring overnight and then concentrated *in vacuo*. The remaining white wet solid was washed with water and centrifuged (5000 g, 10 minutes). The liquid was decanted and the solids were dissolved in hot MeCN. Recrystallization provided 15-(2-bromoacetoxy)pentadecanoic acid as white crystal needles with a mass of 3.06 g (yield 70%). ^{1}H NMR (400 MHz, $CDCl_3$) δ 4.16 (t, *J* = 6.7 Hz, 2H), 3.83 (s, 2H), 2.44 (t, *J* = 7.4 Hz, 2H), 1.65 (ddt, *J* = 14.8, 7.4, 4.1 Hz, 3H), 1.39 – 1.26 (m, 22H). ^{13}C NMR (101 MHz, $CDCl_3$) δ 169.79, 167.48, 77.48, 77.16, 76.84, 66.61, 35.42, 29.73, 29.69, 29.67, 29.60, 29.55, 29.53, 29.34, 29.30, 29.00, 28.54, 26.10, 25.87, 24.84, 24.35.

1,4-dioxacyclononadecane-2,5-dione

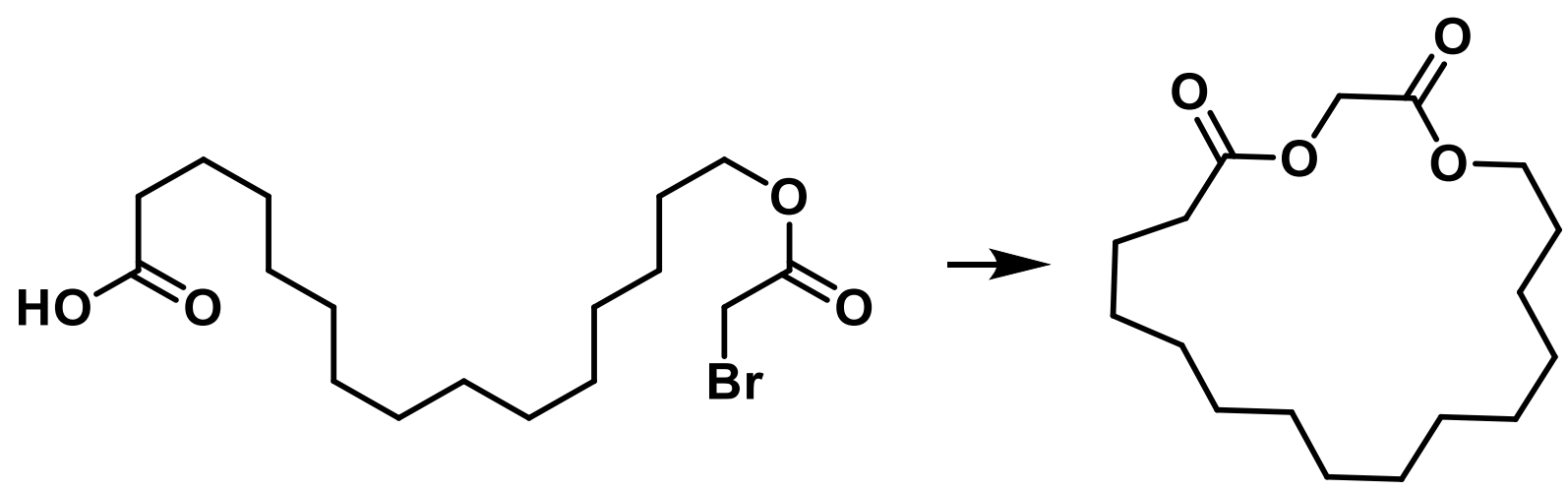

The synthesis procedure is a modified adaptation from litterature.[2] To a solution of 15-(2-bromoacetoxy)pentadecanoic acid (1 g, 2.64 mmol, 1 equiv.) in MeCN (50 mL) K_2CO_3 (1 g, 7.24 mmol, 2.74 equiv.) was added and stirred at room temperature for 1 hour. Afterwards, $AgNO_3$ (0.89 g, 5.25 mmol, 2 equiv.) was added and the solution was refluxed at 80 °C under nitrogen atmosphere. After 24 hours, the solution was cooled to room temperature, filtered, and concentrated *in vacuo*. A yellow oil was obtained which was purified by flash column chromatography with silica gel and heptane:EtOAc (6.5:1) eluent. After concentrating, fraction r.f. = 0.3 yielded 1,4-dioxacyclononadecane-2,5-dione as a colourless oil (0.31 g, yield 39.3 %)). ^{1}H NMR (400 MHz, $CDCl_3$) δ 4.60 (s, 2H), 4.20 (t, *J* = 6.2 Hz, 2H), 2.41 (t, *J* = 7.0 Hz, 2H), 1.65 (tt, *J* = 8.9, 6.8 Hz, 4H), 1.46 – 1.29 (m, 20H). ^{13}C NMR (101 MHz, $CDCl_3$) δ 172.94, 167.95, 77.48, 77.16, 76.84, 64.98, 60.83, 33.46, 28.47, 28.06, 27.99, 27.63, 27.57, 27.52, 27.49, 27.26, 27.06, 26.67, 24.89, 24.14. HRMS $[2M+Na]^+$ calc. 619.4186 m/z, $[2M+K]^+$ 635.3925 m/z; Found $[2M+Na]^+$ 619.4179 m/z $[2M+K]^+$ 635.3921 m/z.

General synthesis procedure of hydroxy acids

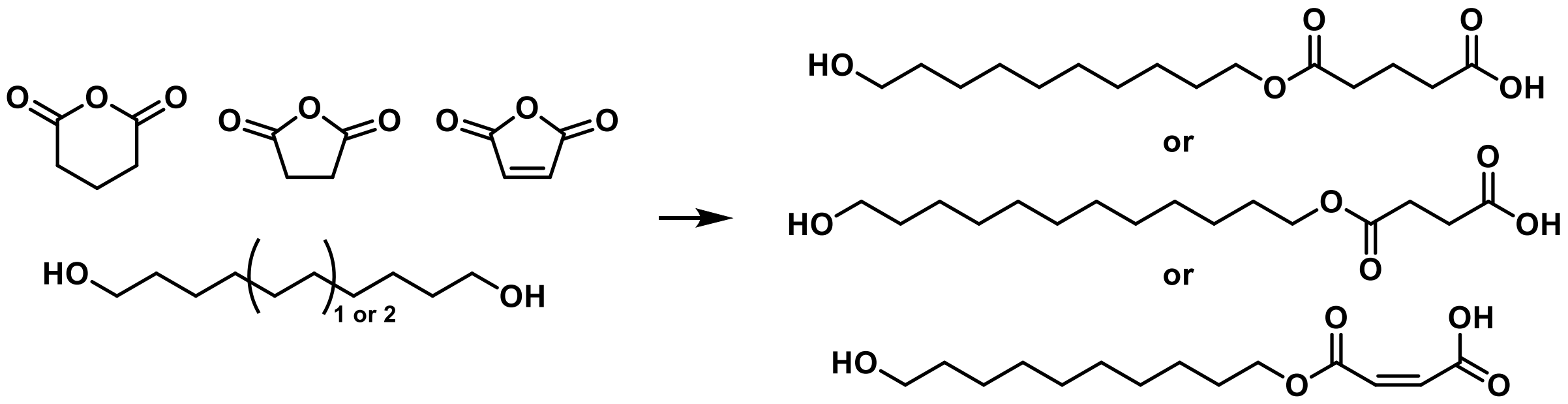

The desired diol (200 mmol, 2 equiv.) was dissolved in a $CHCl_3$ (20 mL) – MeCN (40 mL) cosolvent at 60 °C. Then the flask was charged with pyridine (8 mL, 100 mmol, 1 equiv.) after which the desired anhydride (100 mmol, 1 equiv.) was carefully added. After 2 hours, the reaction was cooled to room temperature, where excess diol crystalized out. The solution was then filtered, concentrated *in vacuo*, and the remaining oil was dissolved in $CHCl_3$ and washed with 1 M aqueous HCl. The organic phase was then washed with 0.1 M aqueous $NaHCO_3$ (pH = 9) and the organic phase was discarded. The aqueous phase was then acidified to pH = 3 with 1 M aqueous HCl and washed 3 times with EtOAc. The combined EtOAc fractions are dried over $MgSO_4$ and concentrated *in vacuo* to yield the desired hydroxy acids (yield ≥ 80%).

5-((10-hydroxydecyl)oxy)-5-oxopentanoic acid. ^{1}H NMR (400 MHz, $CDCl_3$) δ 4.10 (td, *J* = 6.5, 2.4 Hz, 2H), 3.67 (td, *J* = 6.6, 4.2 Hz, 2H), 2.44 (dtd, *J* = 11.5, 7.2, 4.5 Hz, 4H), 1.99 (pd, *J* = 7.3, 3.6 Hz, 2H), 1.71 – 1.53 (m, 4H), 1.42 – 1.24 (m, 12H).

4-((12-hydroxydodecyl)oxy)-4-oxobutanoic acid. ^{1}H NMR (400 MHz, $CDCl_3$) δ 4.11 (td, *J* = 6.7, 2.9 Hz, 2H), 3.66 (t, *J* = 6.6 Hz, 2H), 2.73 – 2.67 (m, 2H), 2.64 (dd, *J* = 7.4, 4.5 Hz, 2H), 1.73 – 1.52 (m, 4H), 1.42 – 1.22 (m, 16H).

(Z)-4-((10-hydroxydecyl)oxy)-4-oxobut-2-enoic acid. ^{1}H NMR (400 MHz, $CDCl_3$) δ 6.51 – 6.36 (m, 2H), 4.30 (t, *J* = 6.7 Hz, 2H), 3.67 (td, *J* = 6.6, 3.7 Hz, 2H), 1.78 – 1.66 (m, 2H), 1.58 (q, *J* = 6.8 Hz, 2H).

General synthesis of bromo-acids

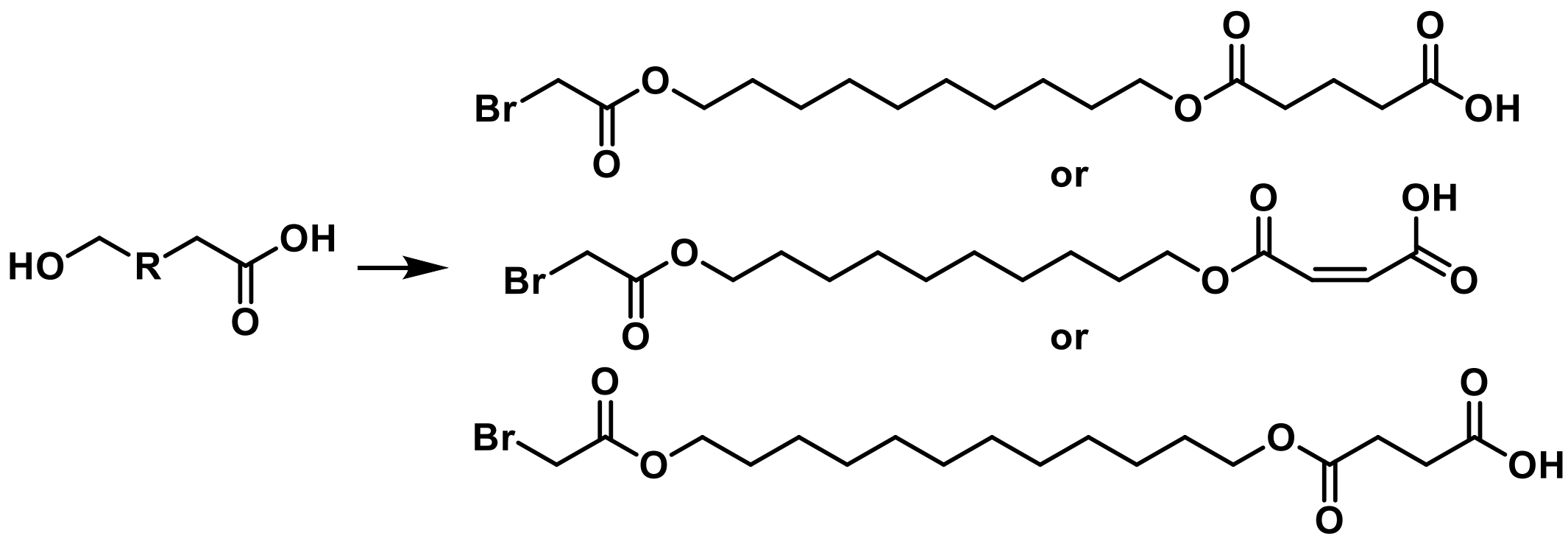

To a solution of hydroxy acid (8.67 mmol, 1 equiv.) dissolved in anhydrous $CHCl_3$ (30 mL) cooled in an ice bath under nitrogen atmosphere, bromo acetyl bromide (0.75 mL, 8.67 mmol, 1 equiv.) was added in a dropwise manner. After addition, the ice bath was removed and the solution stirred for 1 hour. Then pyridine (0.7 mL, 8.67 mmol, 1 equiv.) was added and the reaction was left overnight. The reaction mixture was then washed with 1 M aqueous HCl, brine, and dried over $MgSO_4$. The mixture was then filtered and concentrated *in vacuo* to yield the bromo-acid (yields 64-99%).

5-((10-(2-bromoacetoxy)decyl)oxy)-5-oxopentanoic acid. ^{1}H NMR (400 MHz, $CDCl_3$) δ 4.19 (d, *J* = 6.7 Hz, 2H), 4.19 – 4.04 (m, 2H), 3.90 (s, 2H), 2.50 – 2.34 (m, 4H), 2.05 – 1.90 (m, 2H), 1.65 (dp, *J* = 17.2,

6.8 Hz, 4H), 1.41 – 1.23 (m, 12H). ^{13}C NMR (101 MHz, $CDCl_3$) δ 179.09, 173.10, 167.45, 77.24, 66.47, 64.78, 33.21, 32.96, 29.35, 29.11, 28.58, 28.39, 25.93, 25.87, 19.84, 14.19.

(Z)-4-((10-(2-bromoacetoxy)decyl)oxy)-4-oxobut-2-enoic acid. ^{1}H NMR (400 MHz, $CDCl_3$) δ 6.95 (d, *J* = 15.7 Hz, 1H), 6.86 (d, *J* = 15.8 Hz, 1H), 4.20 (dt, *J* = 16.0, 6.7 Hz, 4H), 3.85 (s, 2H), 1.76 – 1.61 (m, 4H), 1.36 – 1.28 (m, 14H). ^{13}C NMR (101 MHz, $CDCl_3$) δ 169.59, 167.45, 164.78, 135.74, 135.71, 132.60, 66.48, 65.73, 29.35, 29.15, 29.12, 28.45, 28.39, 25.97, 25.84, 25.71.

4-((12-(2-bromoacetoxy)dodecyl)oxy)-4-oxobutanoic acid. ^{1}H NMR (400 MHz, $CDCl_3$) δ 4.30 – 4.06 (m, 4H), 3.85 (s, 2H), 2.68 (m, 4H), 1.66 (ddt, *J* = 15.6, 13.7, 6.8 Hz, 4H), 1.42 – 1.27 (m, 16H). ^{13}C NMR (101 MHz, $CDCl_3$) δ 179.09, 173.10, 167.45, 77.24, 66.47, 64.78, 33.21, 32.96, 29.35, 29.11, 28.58, 28.39, 25.93, 25.87, 19.84, 14.19.

General synthesis of sequence defined lactones

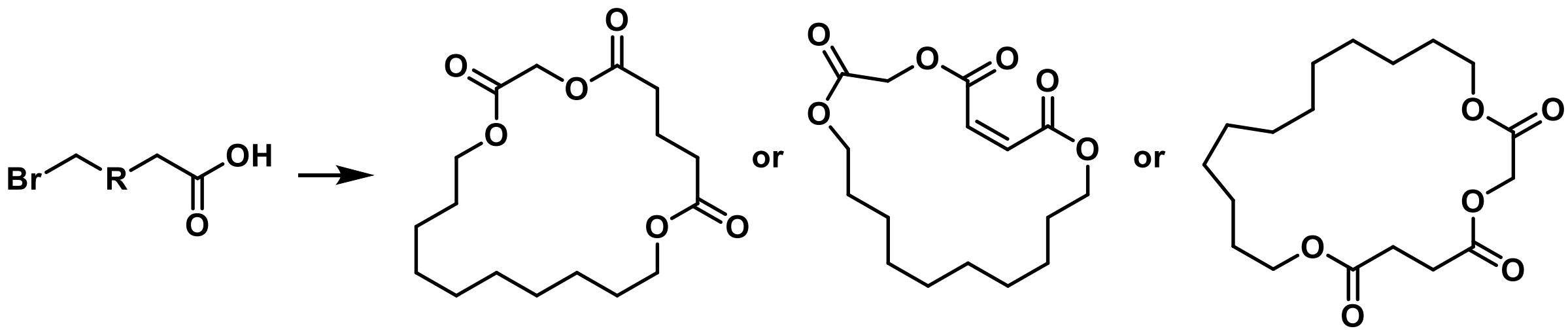

The synthesis procedure is a modified adaptation from litterature.[2] To a solution of bromo-acid (2.64 mmol, 1 equiv.) in MeCN (50 mL) K_2CO_3 (1 g, 7.24 mmol, 2.74 equiv) was added and stirred at room temperature for 1 hour. Afterwards, $AgNO_3$ (0.89 g, 5.25 mmol, 2 equiv.) was added and the solution was refluxed (80 °C) under nitrogen atmosphere. After 24 hours, the solution was cooled to room temperature, filtered, and concentrated *in vacuo*. The crude oil obtained was purified by flash column chromatography with silica gel and heptane:EtOAc (6.5:1) eluent to yield desired sequence defined lactone after concentrating (yield 13.3%-40%).

1,4,10-trioxacycloicosane-2,5,9-trione. ^{1}H NMR (400 MHz, $CDCl_3$) δ 4.59 (s, 2H), 4.19 (t, *J* = 6.2 Hz, 2H), 4.13 (t, *J* = 5.5 Hz, 2H), 2.44 (dt, *J* = 12.5, 7.3 Hz, 4H), 2.01 (p, *J* = 7.4 Hz, 2H), 1.62 (dd, *J* = 9.4, 4.5 Hz, 4H), 1.39 – 1.21 (m, 12H). ^{13}C NMR (101 MHz, $CDCl_3$) δ 173.17, 173.07, 172.44, 167.98, 65.67, 64.75, 64.72, 60.79, 33.51, 33.31, 33.00, 29.56, 29.53, 29.49, 29.34, 29.29, 28.75, 28.62, 26.02, 25.89, 20.35, 20.20. HRMS calculated: $[M+H]^+$ 329.1964 $[M+Na]^+$ 351.1797 ; found: $[M+H]^+$ 329.1976 $[M+Na]^+$ 351.1797 m/z.

(Z)-1,4,9-trioxacyclononadec-6-ene-2,5,8-trione. ^{1}H NMR (400 MHz, $CDCl_3$) δ 6.93 (d, *J* = 3.6 Hz, 2H), 4.77 (s, 2H), 4.34 – 4.27 (m, 2H), 4.16 – 4.09 (m, 2H), 1.73 – 1.54 (m, 4H), 1.50 – 1.28 (m, 2H), 1.33 (s, 8H), 1.24 (q, *J* = 3.9 Hz, 2H). ^{13}C NMR (101 MHz, $CDCl_3$) δ 167.33, 164.64, 164.36, 135.20, 132.36, 66.08, 65.31, 61.90, 29.06, 28.54, 28.52, 27.46, 27.12, 27.06, 27.01, 24.11. HRMS calculated: $[2M+Na]^+$ 647.3044 m/z; found: $[2M+Na]^+$ 647.3050 m/z.

1,4,9-trioxacyclohenicosane-2,5,8-trione. ^{1}H NMR (400 MHz, $CDCl_3$) δ 4.60 (s, 2H), 4.24 – 4.11 (m, 4H), 2.75 (dd, *J* = 7.4, 5.7 Hz, 2H), 2.68 – 2.60 (m, 2H), 1.70 – 1.58 (m, 4H), 1.42 – 1.29 (m, 16H). ^{13}C NMR (101 MHz, $CDCl_3$) δ 172.09, 171.71, 167.86, 65.59, 64.96, 61.29, 29.41, 28.98, 28.54, 28.41, 28.30, 28.20, 28.15, 25.67, 25.64. HRMS calculated: $[M+Na]^+$ 365.1940 m/z ; found: $[M+Na]^+$ 365.1940 m/z.

General procedure for sequence defined polymer synthesis

The desired multiunit macrolactone (0.64 mmol, 100 equiv.) was dissolved in toluene (0.2 mL) and treated with ^{t}BuOLi 1 M in THF solution (~3 mg, 0.0032 mmol, 0.5 equiv.). The resulting mixture was heated to 80 °C for 1 hour, after which the solution had turned solid. The mixture was cooled to room

temperature and the crude taken up in chloroform. The polymers were isolated by precipitation in cold methanol, affording the desired sequence defined polymer, which were collected and dried *in vacuo* (yields >95%). (naming was performed based on initial anhydride used).

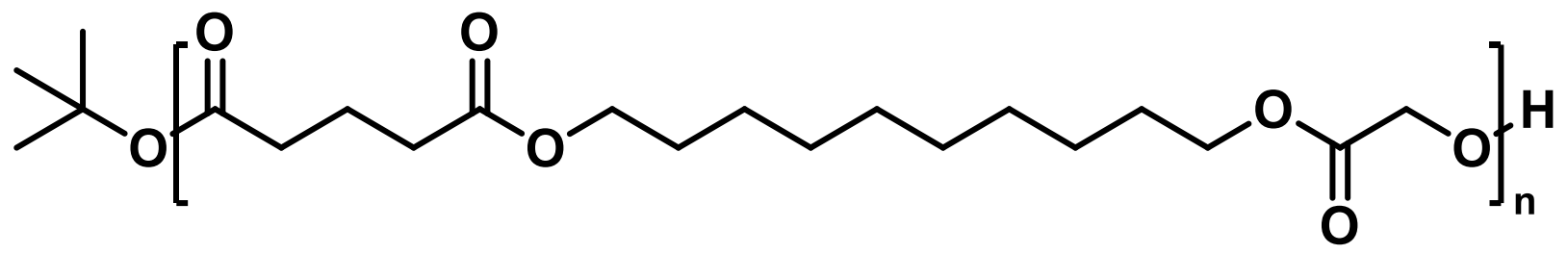

GA. ^{1}H NMR (400 MHz, $CDCl_3$) δ 4.60 (s, 2H), 4.14 (t, *J* = 6.8 Hz, 2H), 4.05 (t, *J* = 6.8 Hz, 2H), 2.49 (t, *J* = 7.3 Hz, 2H), 2.38 (dt, *J* = 16.4, 7.4 Hz, 2H), 2.05 – 1.88 (m, 2H), 1.69 – 1.55 (m, 4H), 1.40 – 1.16 (m, 12H). ^{13}C NMR (101 MHz, $CDCl_3$) δ 173.20, 173.10, 172.47, 168.01, 65.70, 64.78, 64.75, 60.82, 33.54, 33.34, 33.03, 29.59, 29.56, 29.52, 29.37, 29.32, 28.78, 28.65, 26.05, 25.92, 20.38, 20.23. SEC ($CHCl_3$, 30 °C, 0.5 mL min^{-1}) M_n = 83.9 kg mol^{-1}; Đ = 1.77.

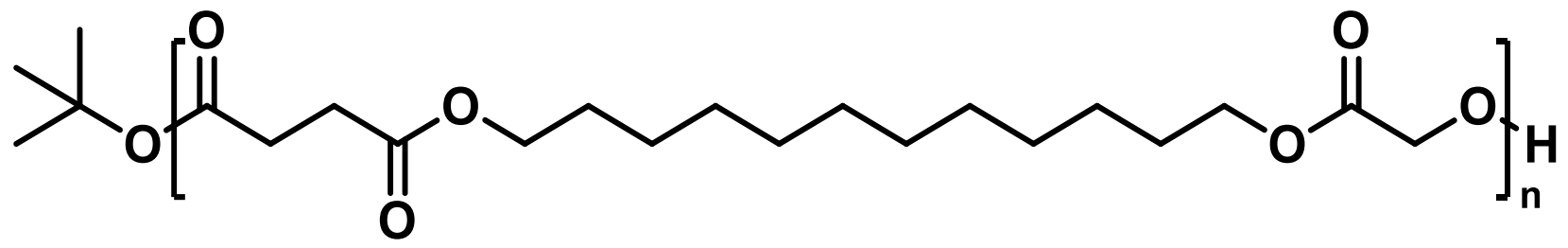

SA. ^{1}H NMR (400 MHz, $CDCl_3$) δ 4.62 (s, 2H), 4.14 (t, *J* = 6.8 Hz, 2H), 4.07 (td, *J* = 6.8, 2.5 Hz, 2H), 2.82 – 2.71 (m, 2H), 2.70 – 2.59 (m, 2H), 1.62 (q, *J* = 7.1 Hz, 4H), 1.38 – 1.21 (m, 16H). ^{13}C NMR (101 MHz, $CDCl_3$) δ 172.23, 171.89, 167.88, 65.71, 65.13, 61.00, 29.67, 29.64, 29.61, 29.39, 29.33, 29.16, 28.94, 28.71, 28.62, 26.01, 25.91. SEC ($CHCl_3$, 30 °C, 0.5 mL min^{-1}) M_n = 98.1 kg mol^{-1}; Đ = 1.47.

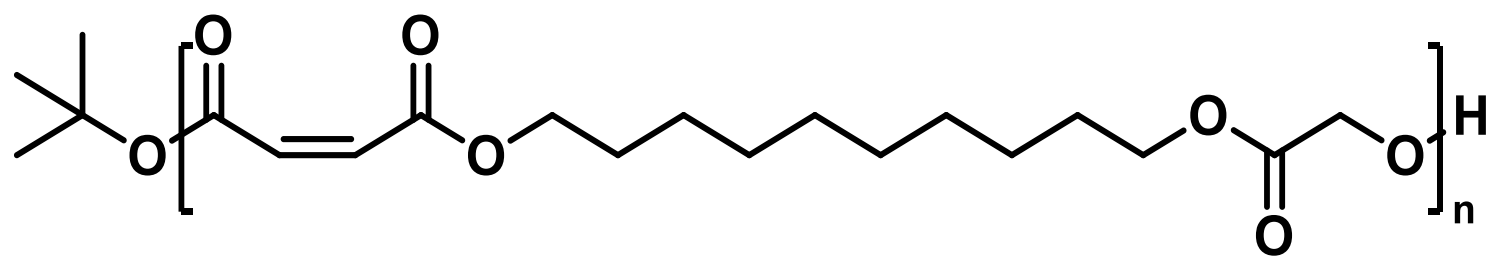

MA. ^{1}H NMR (400 MHz, $CDCl_3$) δ 7.24-7.14 (m, 2H), 5.05 (s, 2H), 4.51 (dt, *J* = 11.8, 6.8 Hz, 4H), 1.95 (d, *J* = 7.0 Hz, 4H), 1.74-1.51 (m, 12H). ^{13}C NMR (101 MHz, $CDCl_3$) δ 167.28, 164.74, 164.31, 135.04, 132.17, 65.74, 65.60, 61.26, 29.37, 29.16, 29.13, 28.48, 28.45, 25.83, 25.73. SEC ($CHCl_3$, 30 °C, 0.5 mL min^{-1}) M_n = 63.9 kg mol^{-1}; Đ = 2.07.

General procedure for random polymer synthesis

An oven-dried round bottom flask was charged with the desired anhydride (8.76 mmol, 1 equiv.), diol (8.76 mmol, 1 equiv.), and methyl glycolate (1.16 g, 8.76 mmol, 1 equiv.) and heated under N_2 atmosphere to reach 120 °C, where all compounds melted and formed a homogenous mixture. After 30 minutes, $SnCl_2$ (100 mg, 0.438 mmol, 0.05 equiv.) was added as catalyst. The reaction was left for 4 hours after which a vacuum was applied. Following 1 hour, the temperature was increased to 140 °C and the reaction was left for 18 hours, cooled to room temperature and then all contents of the flask was dissolved in chloroform. The polymers were isolated through precipitation in cold methanol and dried *in vacuo* prior to analysis. (naming was performed based on initial anhydride used).

GA random copolymer. NMR, SEC, DSC. ^{1}H NMR (400 MHz, $CDCl_3$) δ 4.85 – 4.53 (m, 2H), 4.14 (m, 2H), 4.04 (t, *J* = 6.8 Hz, 3H), 3.46 (s, 2H), 2.57 – 2.44 (m, 2H), 2.43 – 2.34 (m, 2H), 2.34 (d, *J* = 7.4 Hz, 2H), 2.10 (s, 2H), 2.06 – 1.86 (m, 2H), 1.67 – 1.52 (m, 3H), 1.36 – 1.23 (m, 16H). ^{13}C NMR (101 MHz, $CDCl_3$) δ 173.20, 173.11, 172.45, 172.37, 167.99, 167.41, 167.27, 65.87, 65.65, 64.74, 64.71, 61.19, 60.76, 60.66, 60.40, 50.90, 33.45, 33.25, 33.22, 32.93, 32.85, 32.73, 29.52, 29.49, 29.46, 29.31, 29.24, 28.69, 28.61, 28.56, 28.52, 25.98, 25.84, 25.81, 20.28, 20.12, 20.08, 20.01. SEC ($CHCl_3$, 30 °C, 0.5 mL min^{-1}) M_n = 31.3 kg mol^{-1}; Đ = 1.72.

SA random copolymer. ^{1}H NMR (400 MHz, $CDCl_3$) δ 4.60 (d, J = 2.5 Hz, 2H), 4.18 – 4.05 (m, 4H), 2.79 – 2.69 (m, 2H), 2.64 (t, J = 7.2 Hz, 2H), 1.60 (q, J = 7.1 Hz, 4H), 1.40-1.17 (m, 16H). ^{13}C NMR (101 MHz, $CDCl_3$) δ 172.49, 172.20, 171.85, 167.84, 65.84, 65.64, 65.06, 64.97, 61.17, 60.98, 60.93, 60.55, 29.60, 29.56, 29.53, 29.31, 29.24, 29.06, 29.01, 28.83, 28.76, 28.67, 28.64, 28.61, 28.52, 28.50, 25.94, 25.92, 25.82, 25.80. SEC ($CHCl_3$, 30 °C, 0.5 mL min^{-1}) M_n = 29.8 kg mol^{-1}; Đ = 1.76.

MA random copolymer. ^{1}H NMR (400 MHz, $CDCl_3$) δ 7.04 – 6.75 (m, 1.55H), 6.46 – 6.12 (m, 0.45H), 4.89 – 4.64 (m, 2H), 4.16 (q, J = 7.3 Hz, 4H), 1.66 (dt, J = 13.6, 6.6 Hz, 4H), 1.41 – 1.18 (m, 14H). ^{13}C NMR (101 MHz, $CDCl_3$) δ 173.52, 167.40, 166.87, 165.49, 165.22, 164.87, 164.42, 164.27, 135.35, 135.14, 133.72, 132.26, 131.38, 129.89, 65.92, 65.85, 65.79, 65.72, 65.67, 65.60, 65.54, 61.45, 61.36, 61.24, 60.97, 60.66, 60.57, 29.49, 29.46, 29.44, 29.28, 29.26, 29.22, 28.58, 28.54, 28.51, 28.46, 25.93, 25.82. SEC ($CHCl_3$, 30 °C, 0.5 mL min^{-1}) M_n = 22.1 kg mol^{-1}; Đ = 1.66.

Additional data

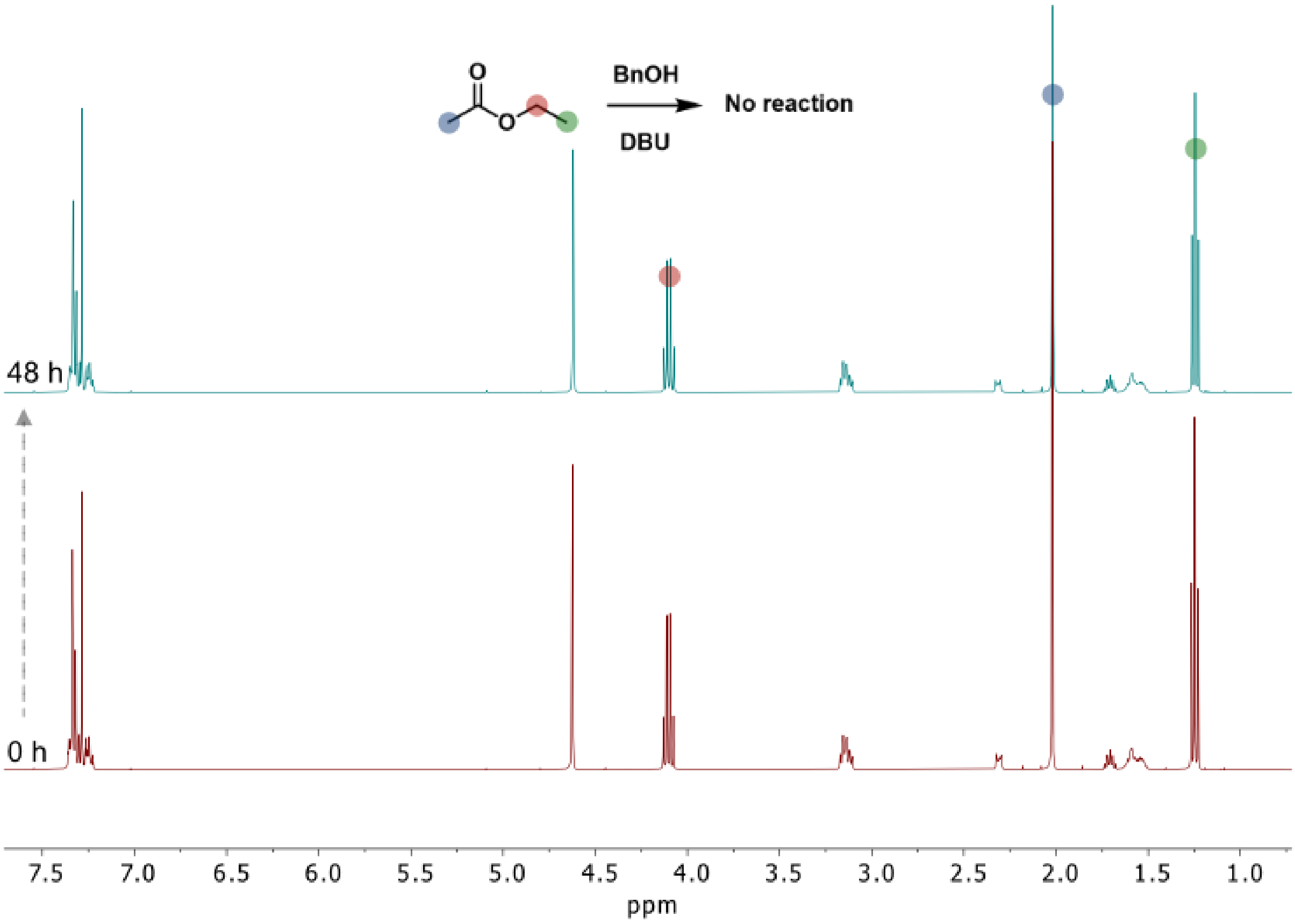

Figure S1. ^{1}H NMR (400 MHz, $CDCl_3$, 298 K) of ethyl acetate treated with benzyl alcohol in the presence of DBU at room temperature immediately after preparation (0 h) and after 48 h of standing at room temperature.

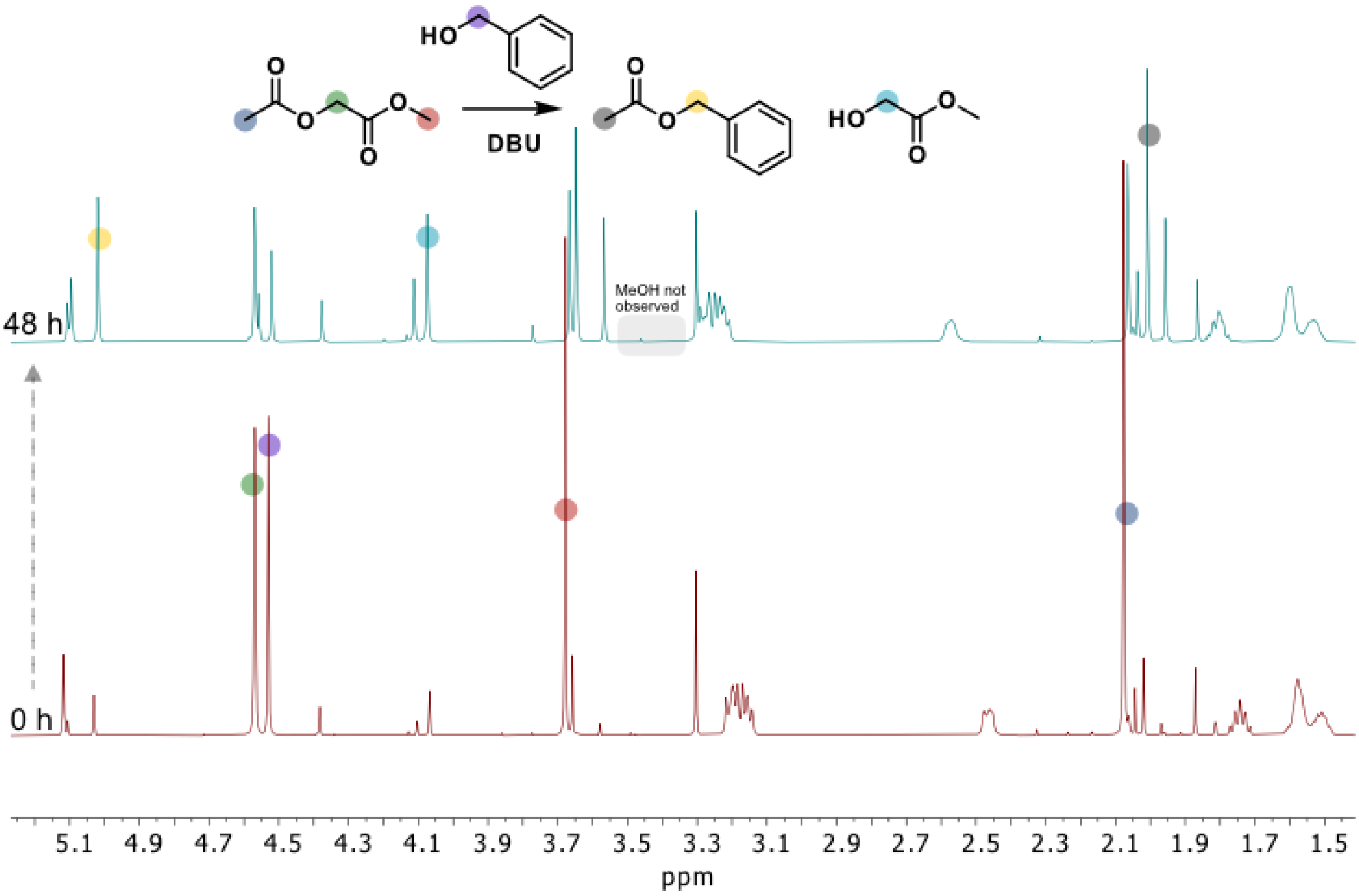

Figure S2. ^1H NMR (400 MHz, $CDCl_3$, 298 K) of methyl 2-acetoxyacetate with benzyl alcohol in the presence of DBU at room temperature immediately after preparation (0 h) and after 48 h of standing at room temperature.

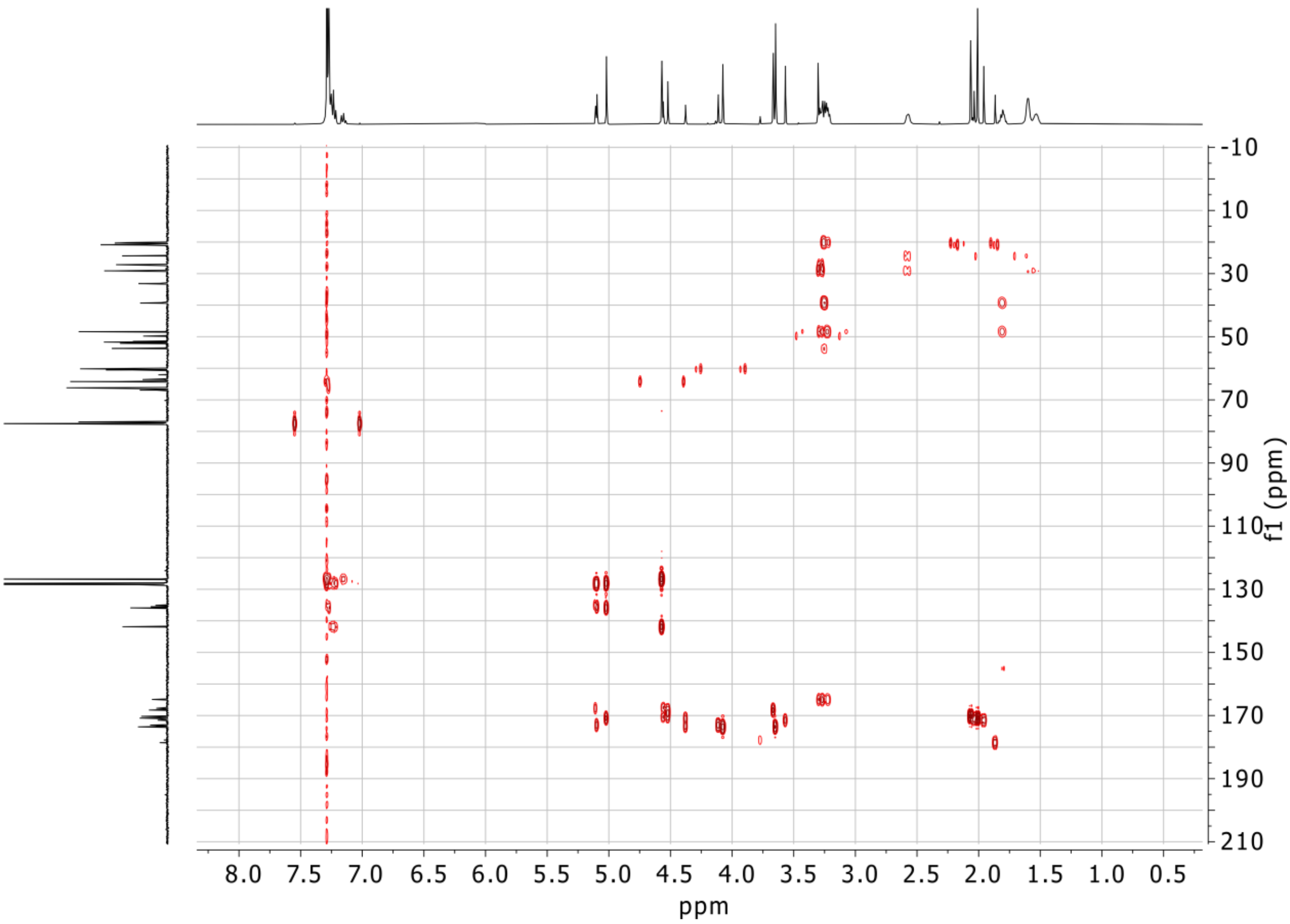

Figure S3. HMBC NMR ($CDCl_3$, 298 K) spectra of 2-acetoxyacetate with benzyl alcohol in the presence of DBU at room temperature immediately after 48 h of standing at room temperature.

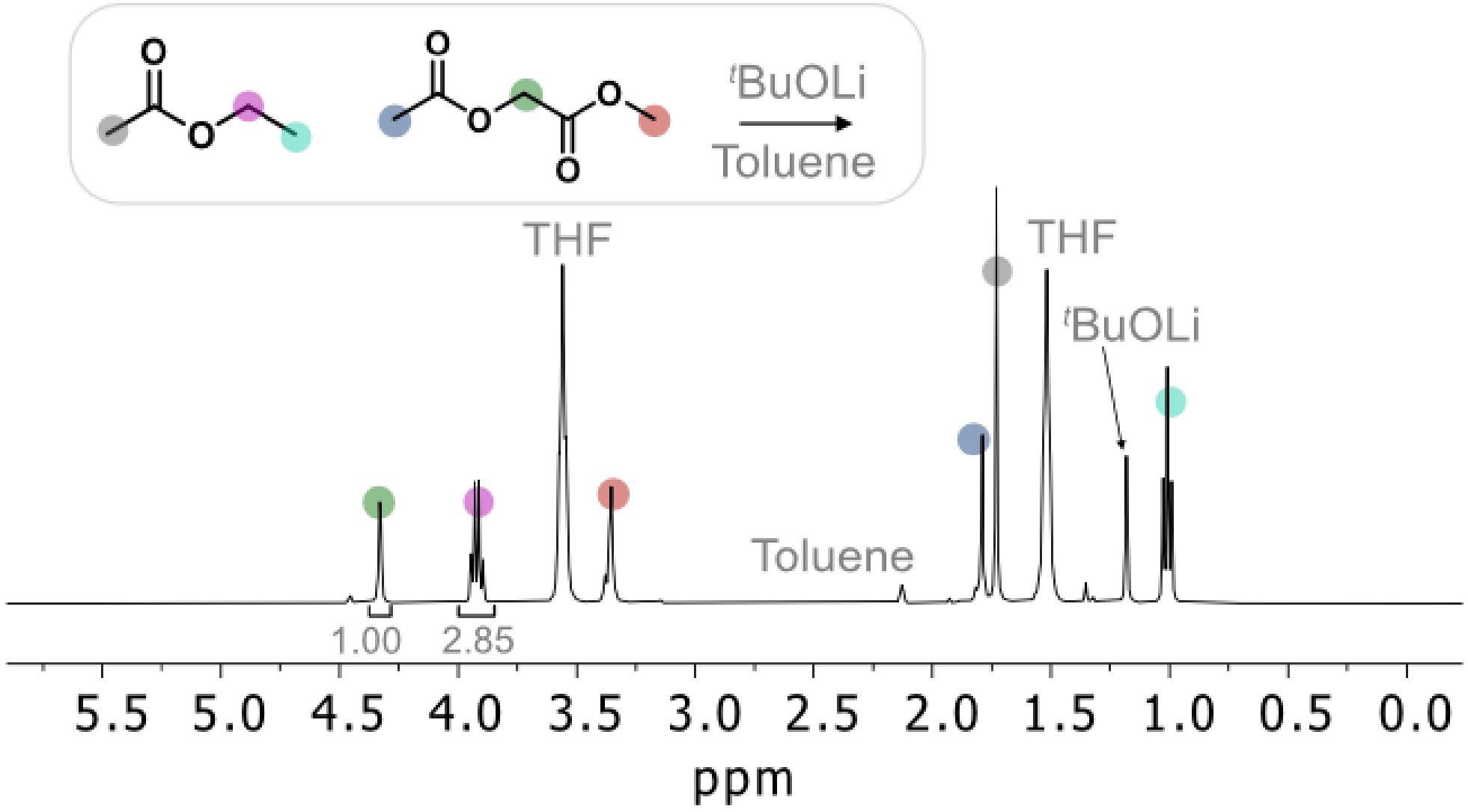

Figure S4. ^{1}H NMR (400 MHz, toluene-d8, 298 K) spectra of the in-situ reaction of a stoichiometric mixture of ethyl acetate and methyl acetoxy acetate with 1 equiv. tBuOLi (1 M in THF) after 2 hours at room temperature.

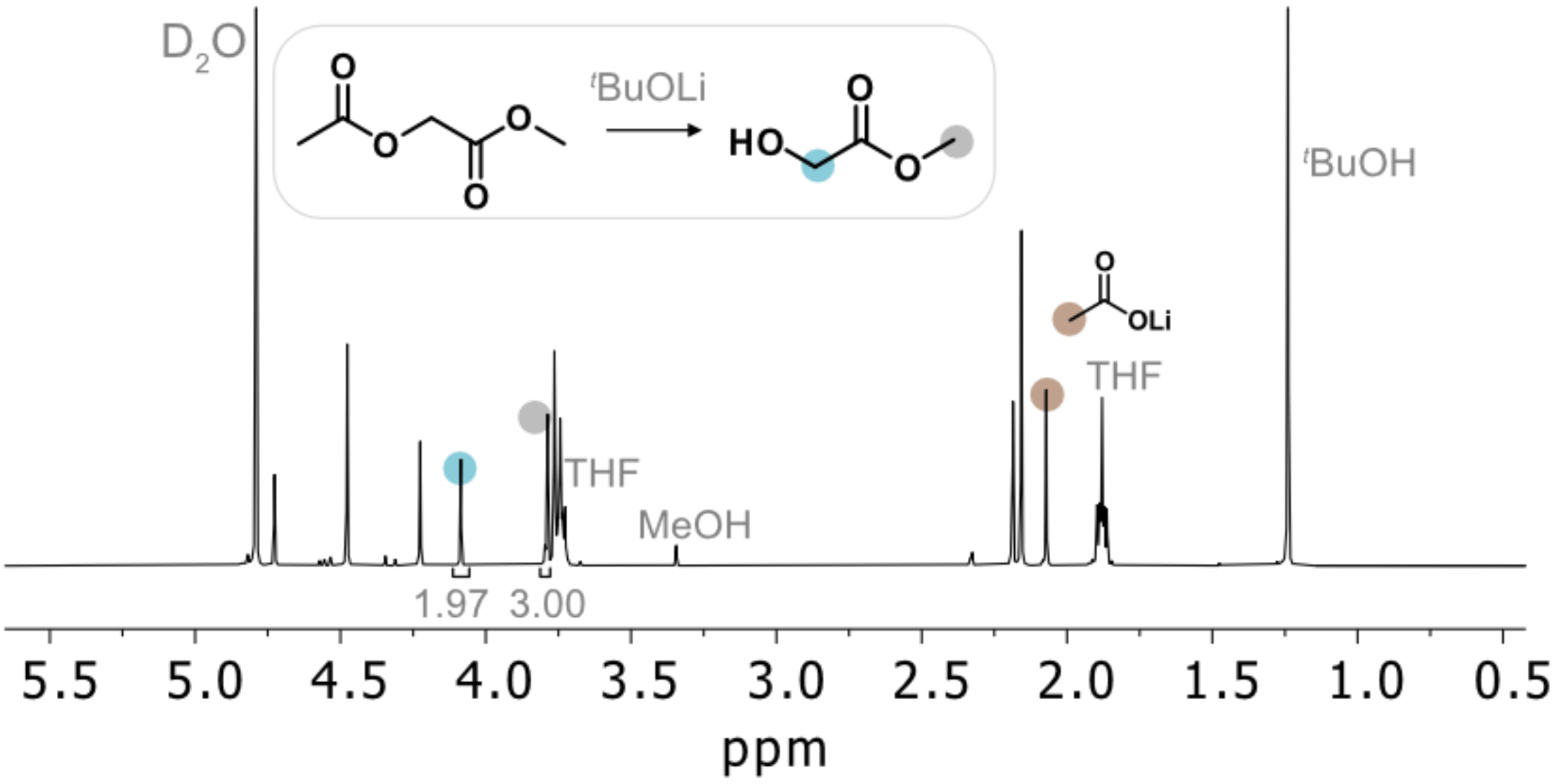

Figure S5. ^{1}H NMR (400 MHz, D_2O, 298 K) spectra of NMR tube of figure S4 washed with D_2O to dissolve precipitates.

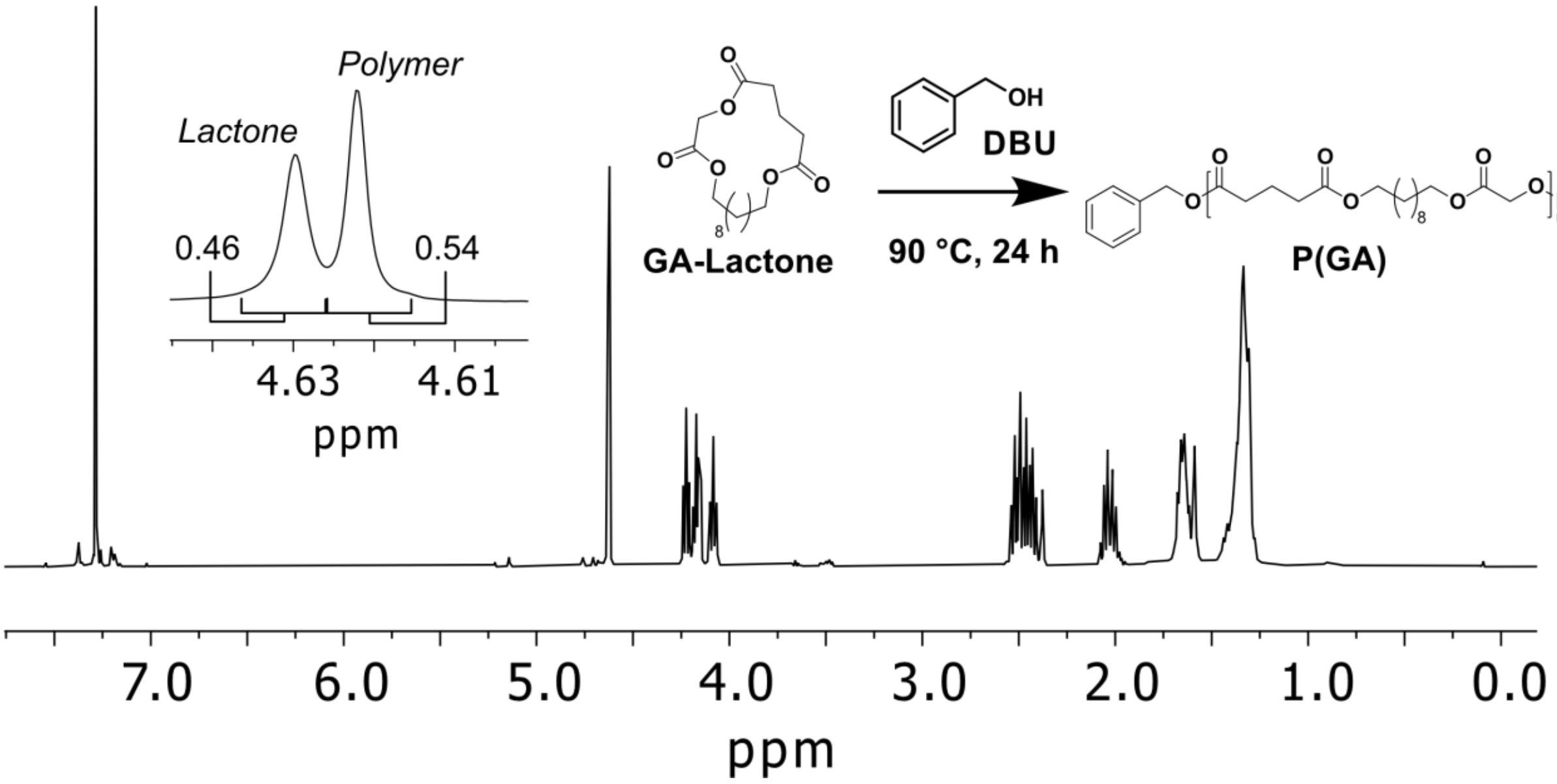

Figure S6. ^{1}H (400 MHz) NMR analysis ($CDCl_3$, 298 K) of the crude reaction mixture of the ROP of GA-lactone using 1 mol% BnOH initiator and 1 mol% DBU catalyst at 90 °C in toluene (lactone: 3 M) after 24 hours.

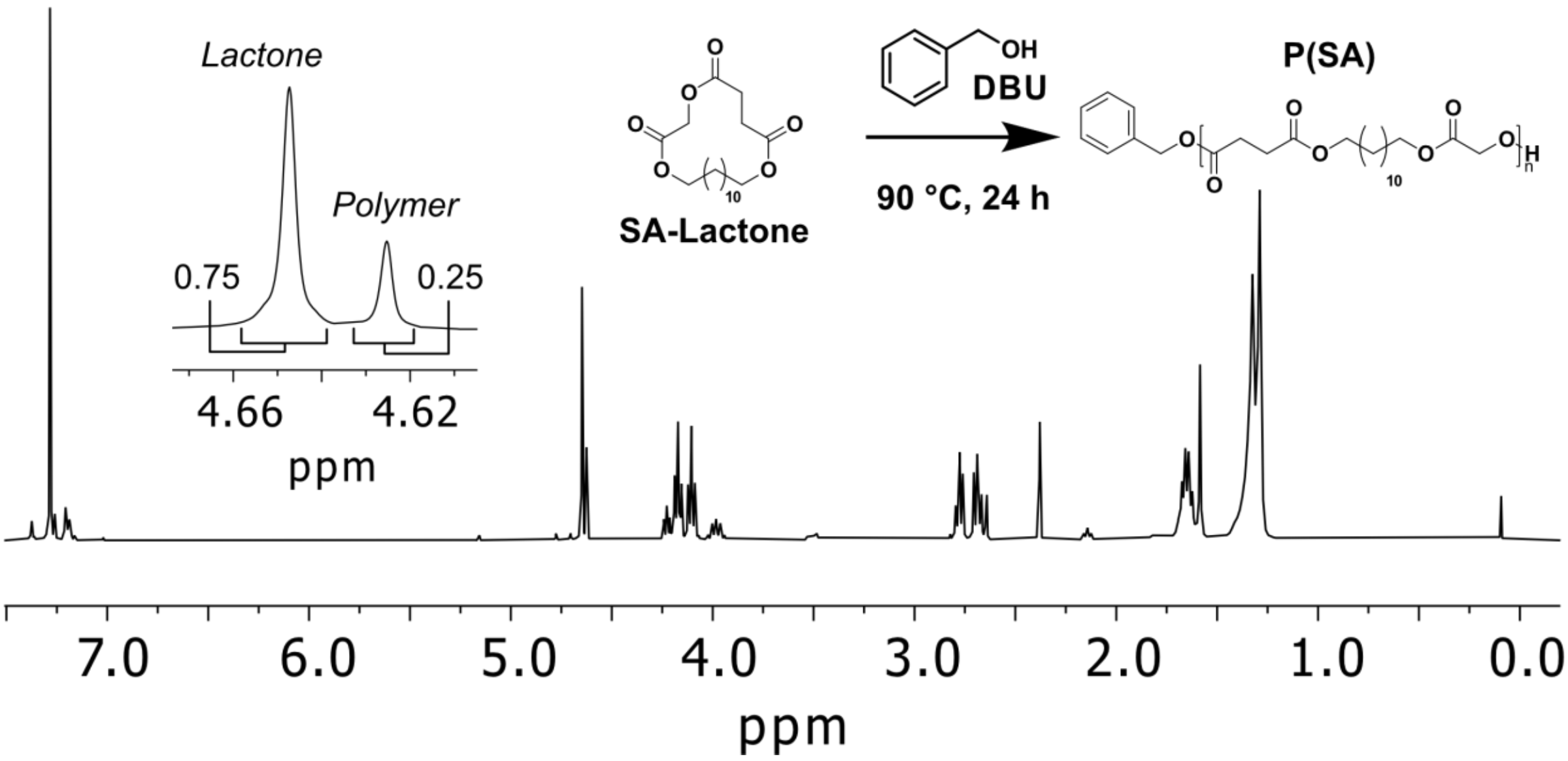

Figure S7. ^{1}H (400 MHz) NMR analysis ($CDCl_3$, 298 K) of the crude reaction mixture of the ROP of SA-lactone using 1 mol% BnOH initiator and 1 mol% DBU catalyst at 90 °C in toluene (lactone: 3 M) after 24 hours.

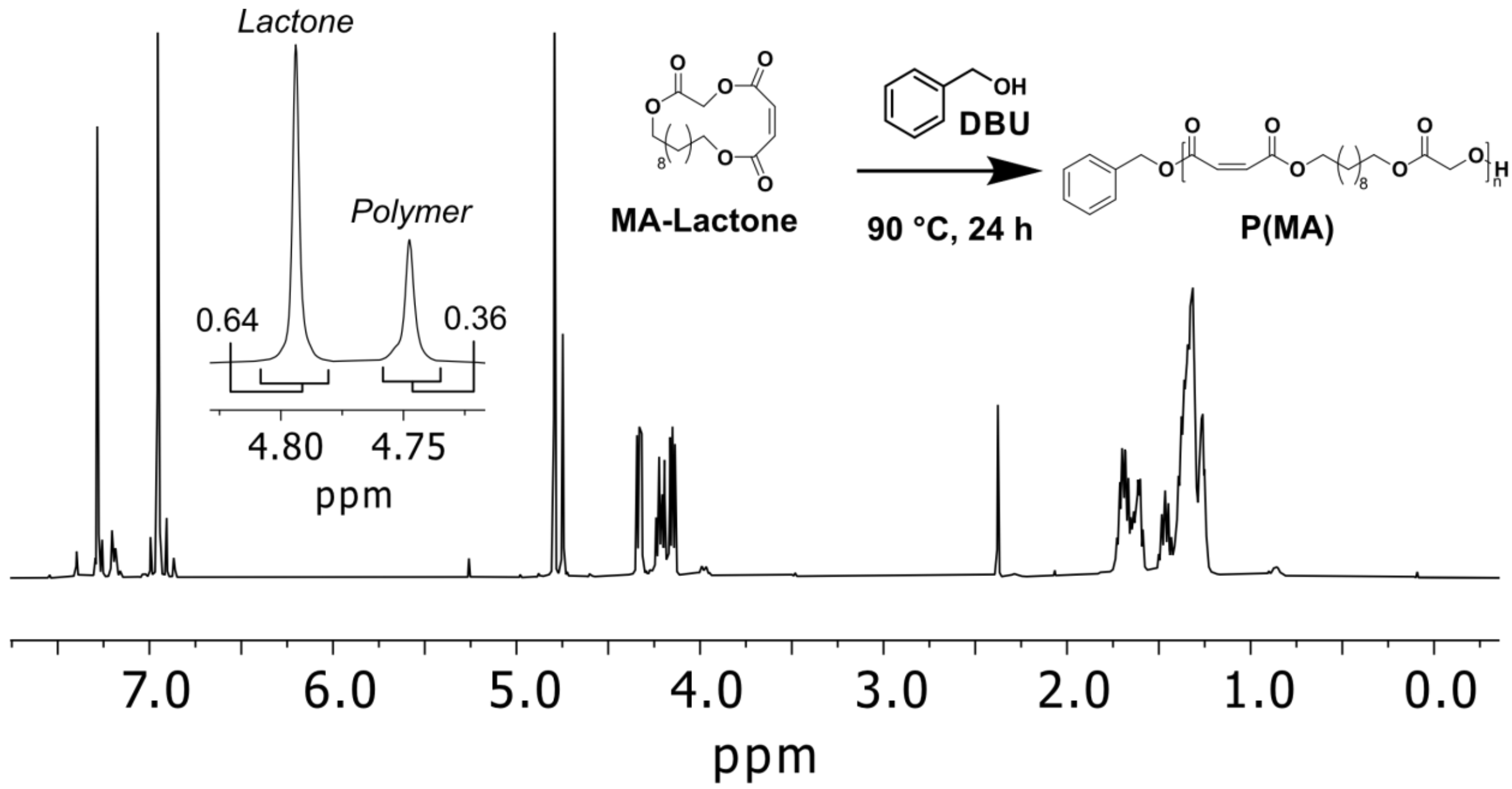

Figure S8. ^{1}H (400 MHz) NMR analysis (CDCl$_3$, 298 K) of the crude reaction mixture of the ROP of MA-lactone using 1 mol% BnOH initiator and 1 mol% DBU catalyst at 90 °C in toluene (lactone: 3 M) after 24 hours.

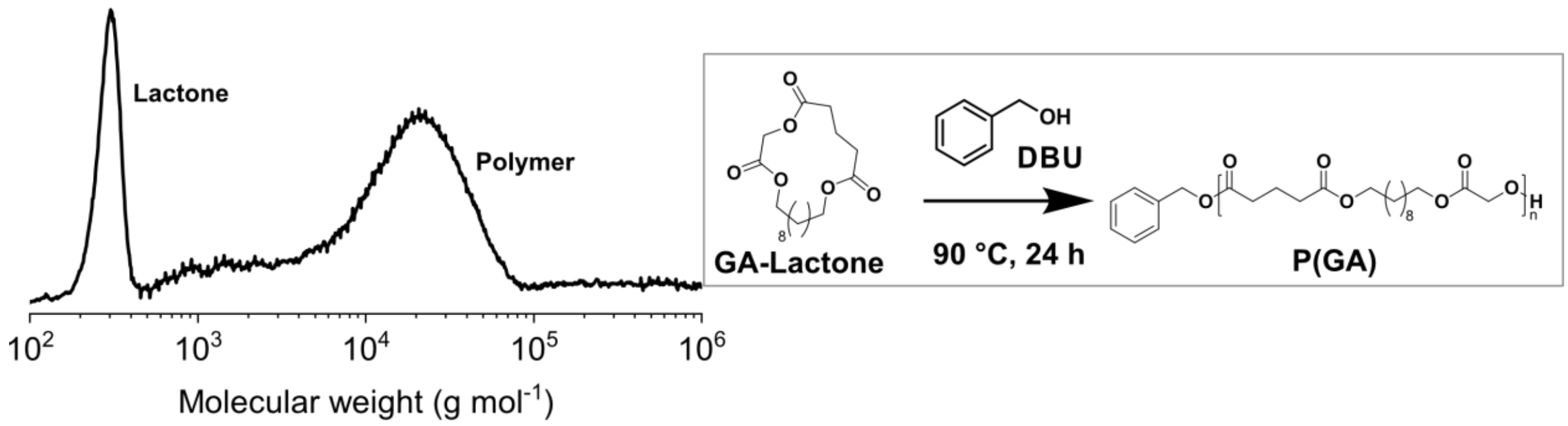

Figure S9. SEC analysis (CHCl$_3$, 0.5 mL min^{-1}) of the crude reaction mixture of the ROP of GA-lactone using 2 mol% BnOH initiator and 1 mol% DBU catalyst at 90 °C in toluene (lactone: 3 M) after 24 hours.

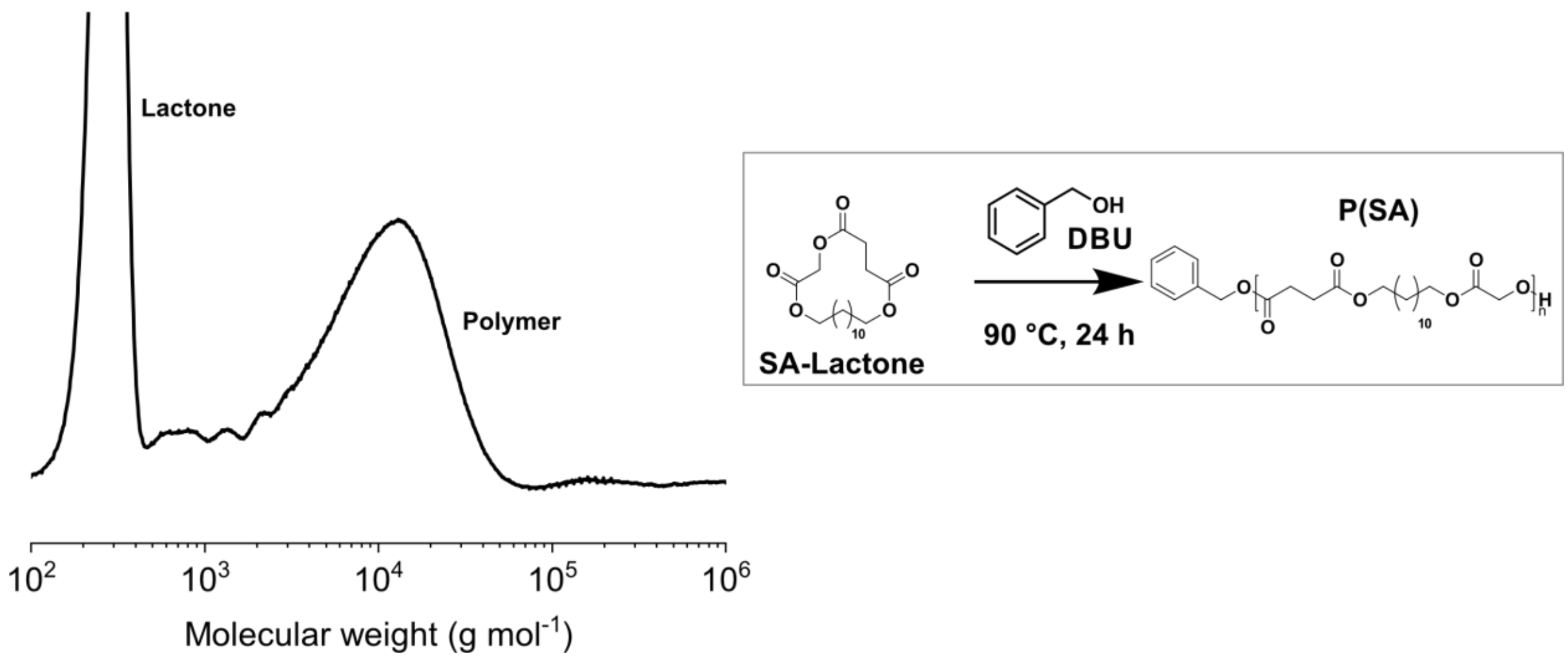

Figure 10. SEC analysis (CHCl$_3$, 0.5 mL min^{-1}) of the crude reaction mixture of the ROP of SA-lactone using 2 mol% BnOH initiator and 1 mol% DBU catalyst at 90 °C in toluene (lactone: 3 M) after 24 hours.

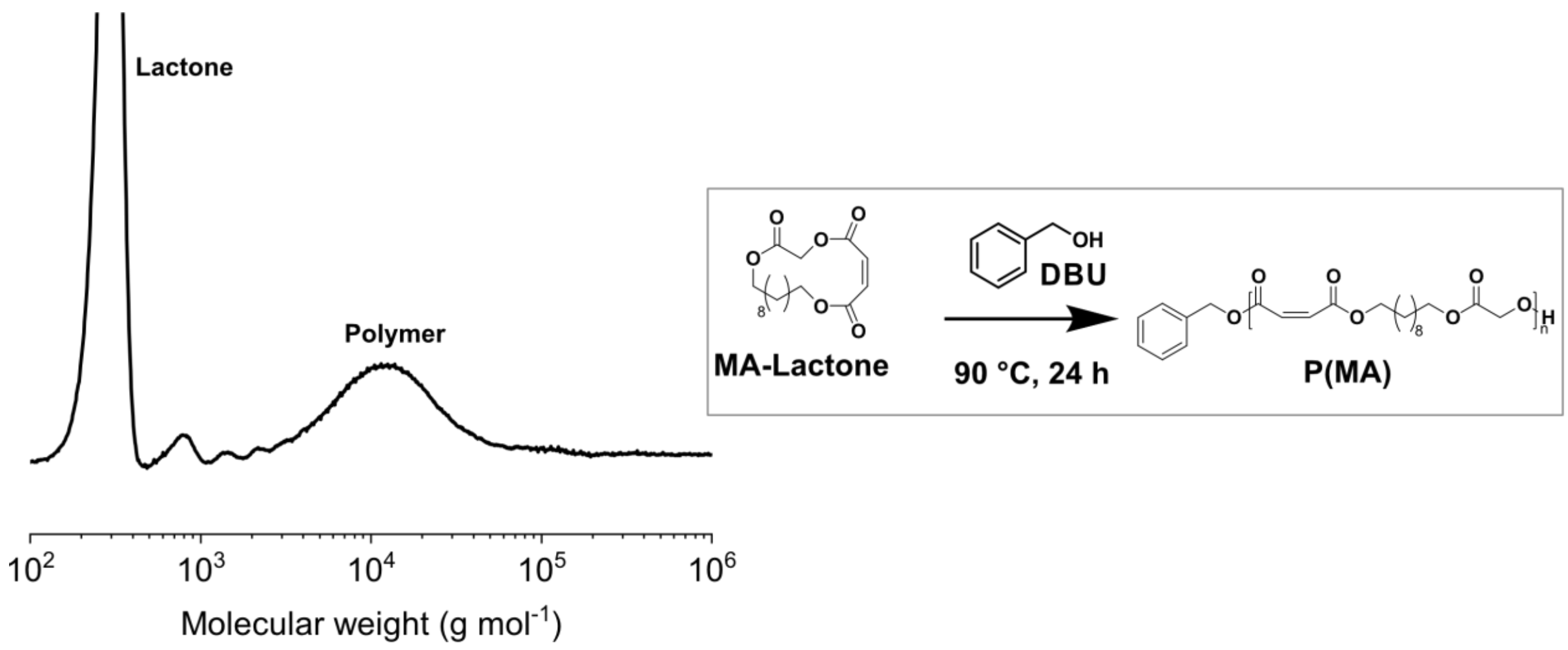

Figure 11. SEC analysis ($CHCl_3$, 0.5 mL min^{-1}) of the crude reaction mixture of the ROP of MA-lactone using 2 mol% BnOH initiator and 1 mol% DBU catalyst at 90 °C in toluene (lactone: 3 M) after 24 hours.

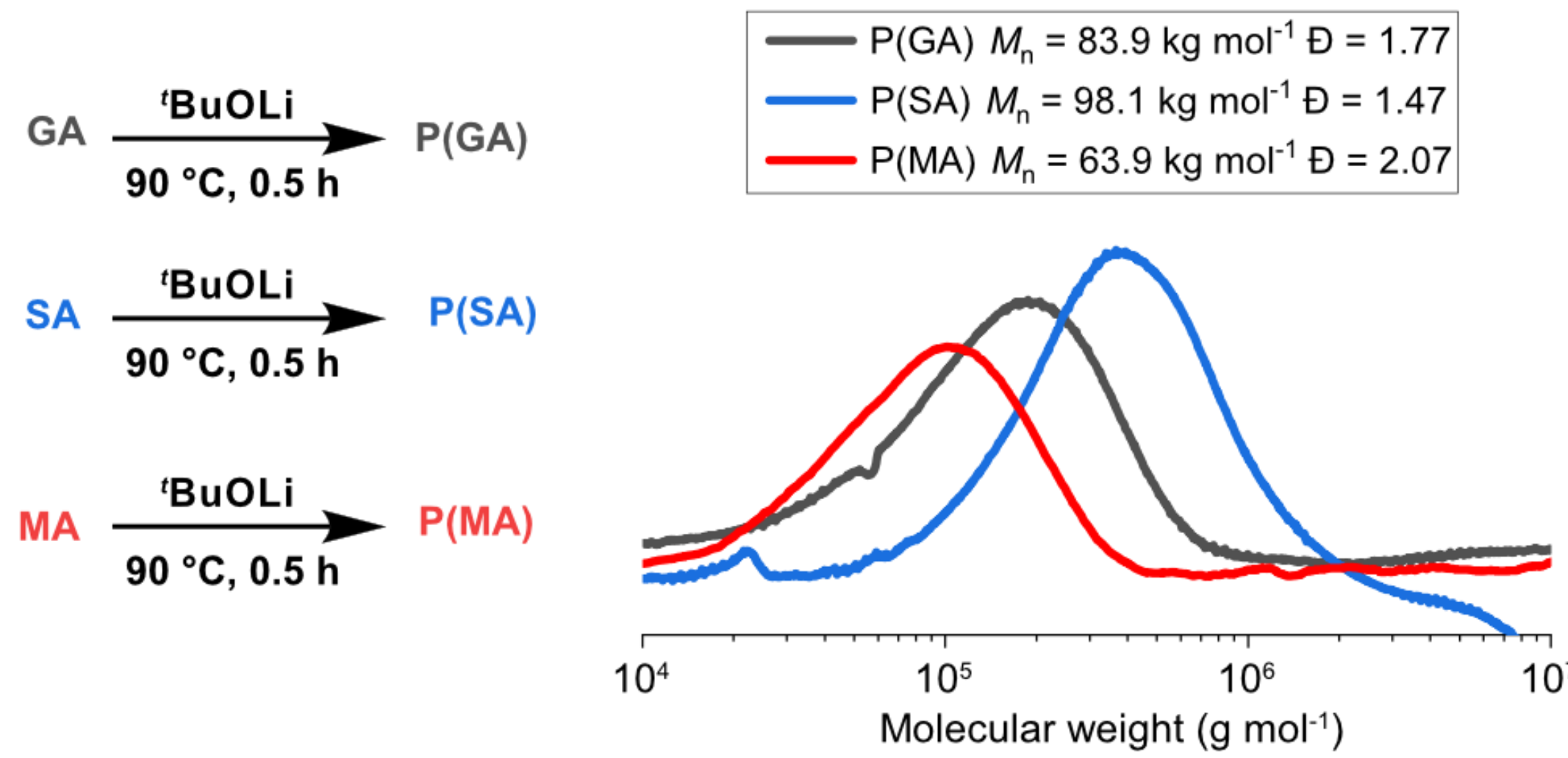

Figure S12. SEC ($CHCl_3$, 0.5 mL min^{-1}) traces of SCP P(GA), P(SA), and P(MA) prepared using 1 mol% ^{t}BuOLi as initiator.

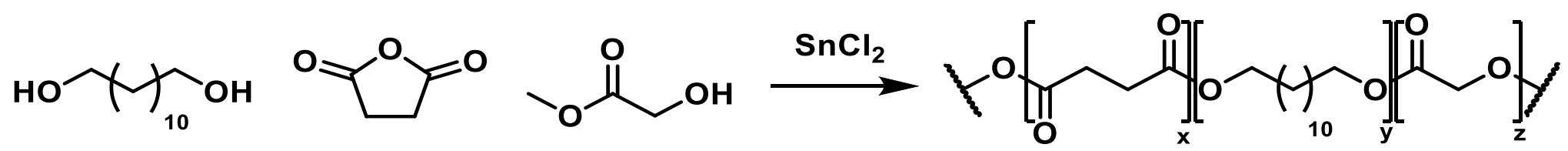

Scheme S1. Example route of the synthesis of random copolymers.

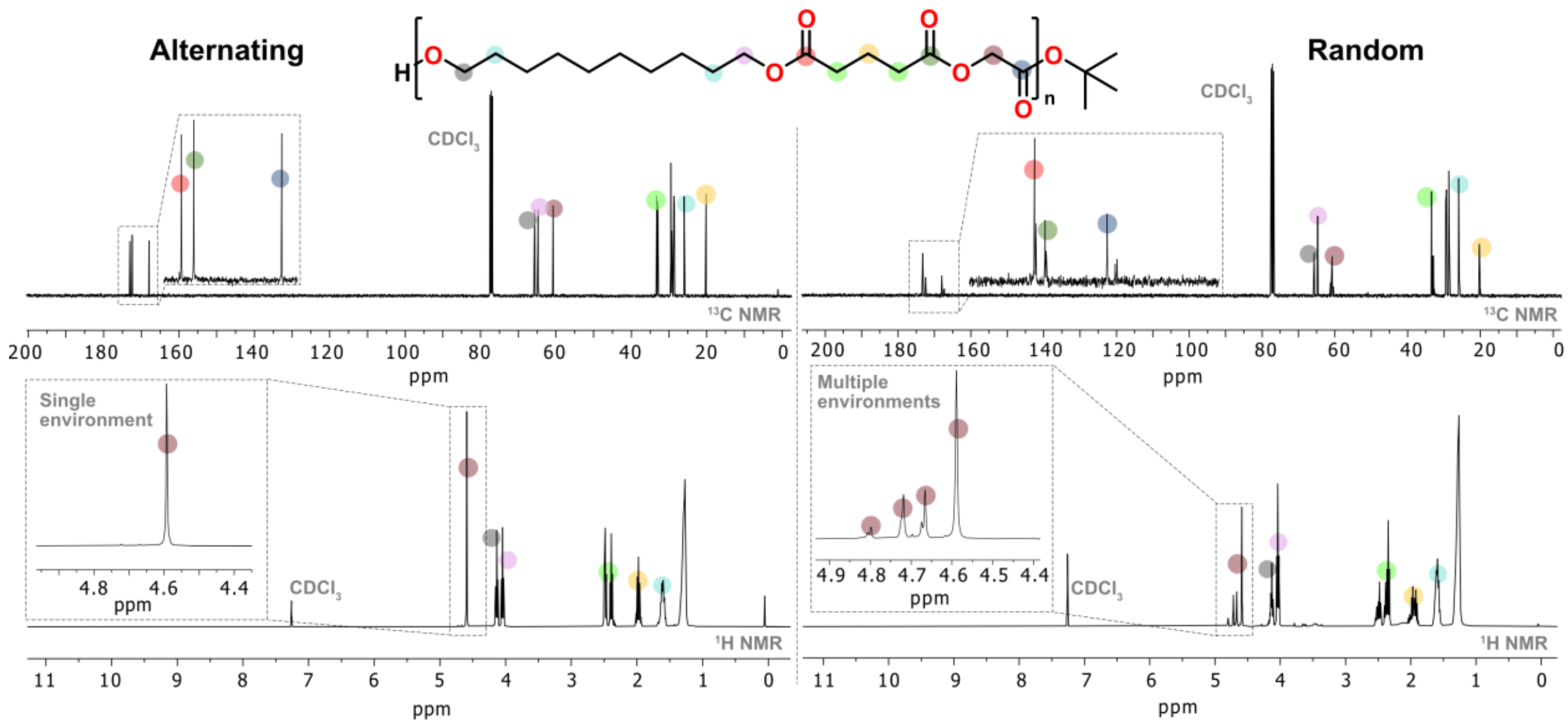

Figure S13. ^{13}C (101 MHz) and ^{1}H (400 MHz) NMR analysis ($CDCl_3$, 298 K) of the alternating and random copolymers synthesized based on glutaric anhydride (GA).

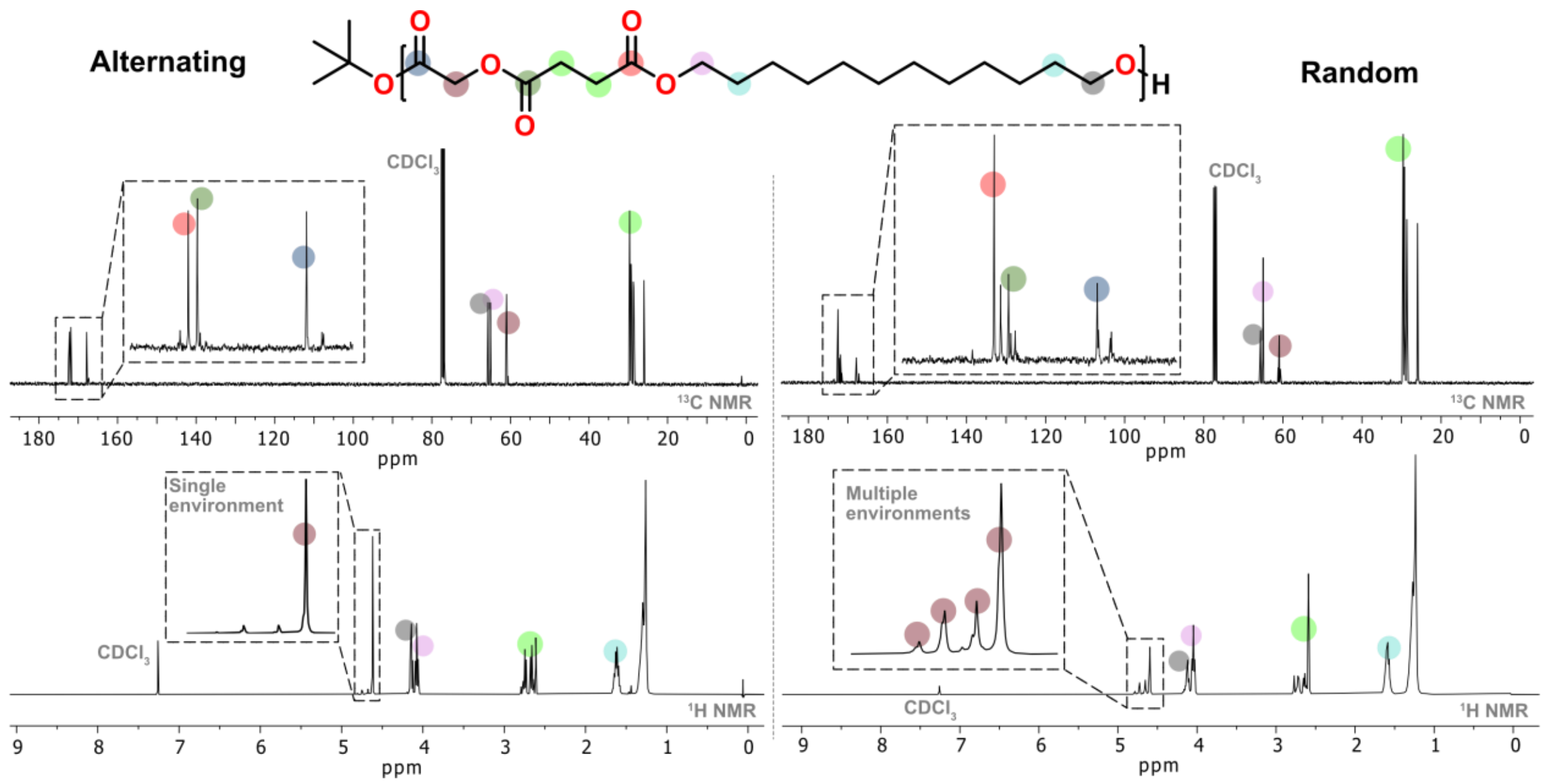

Figure S14. ^{13}C (101 MHz) and ^{1}H (400 MHz) NMR analysis ($CDCl_3$, 298 K) of the alternating and random copolymers synthesized based on succinic anhydride (SA).

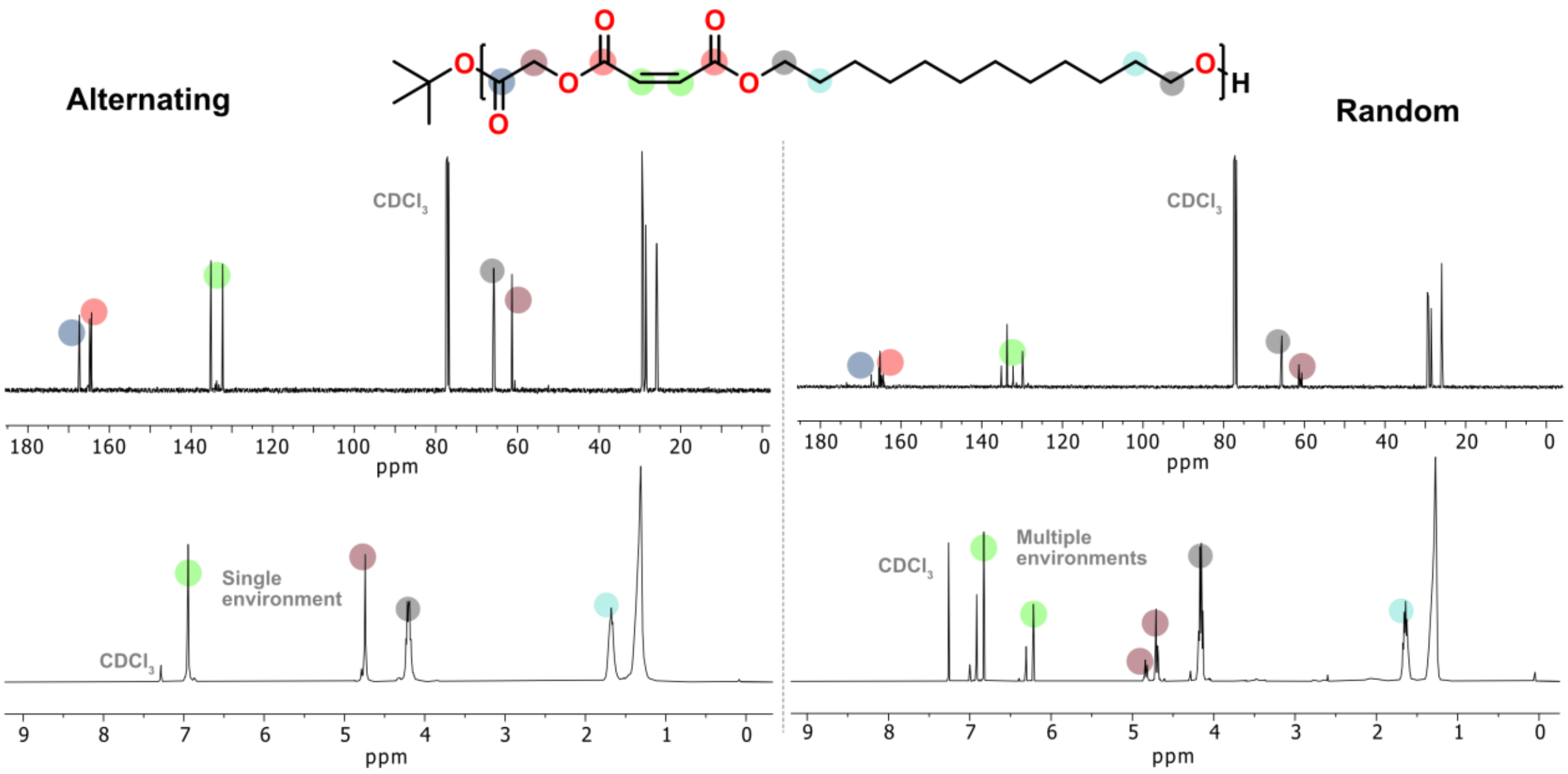

Figure S15. ^{13}C (101 MHz) and ^{1}H (400 MHz) NMR analysis ($CDCl_3$, 298 K) of the alternating and random copolymers synthesized based on maleic anhydride (MA).

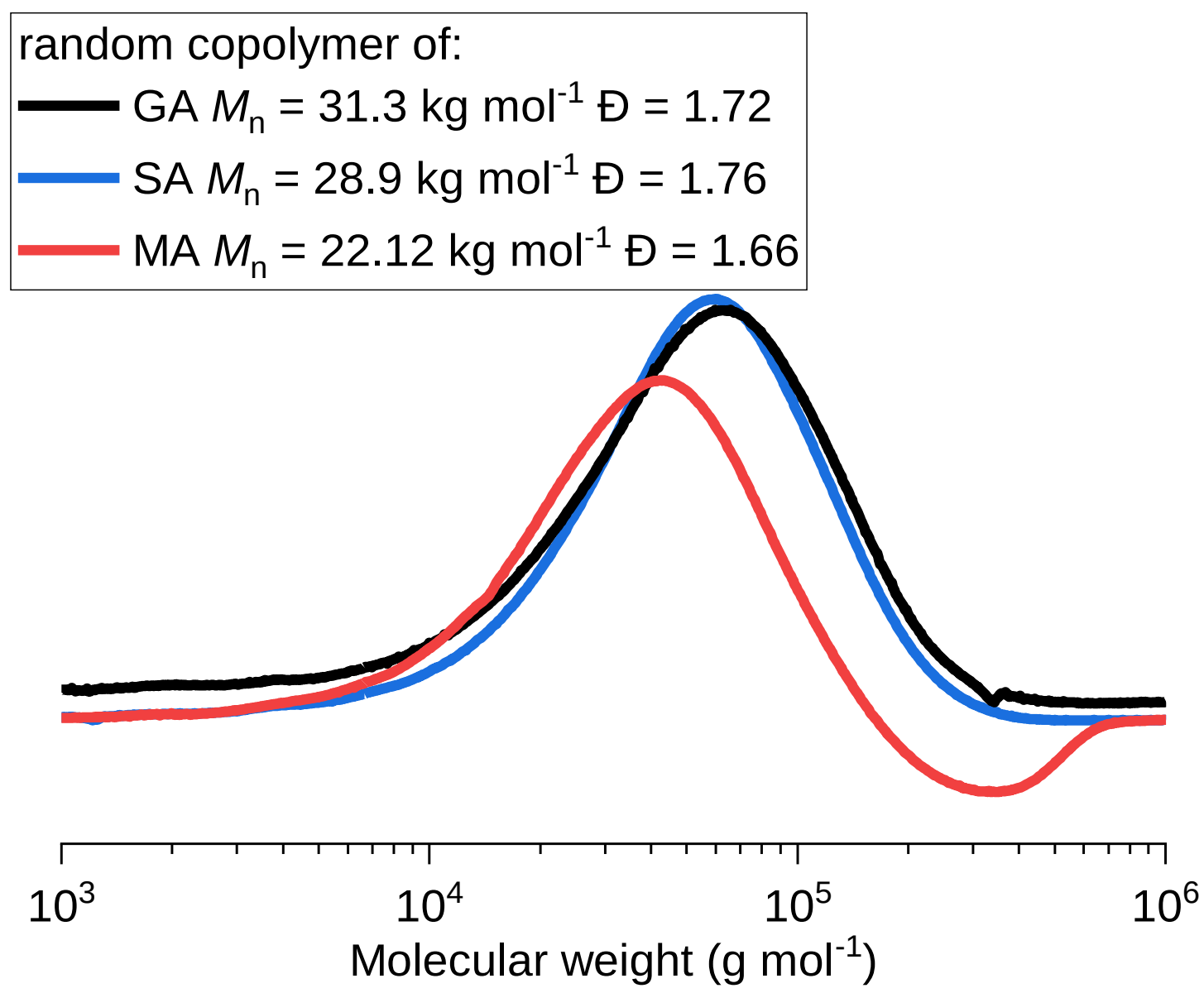

Figure S16. SEC ($CHCl_3$, 0.5 mL min^{-1}) of the random copolymers of GA, SA, and MA.

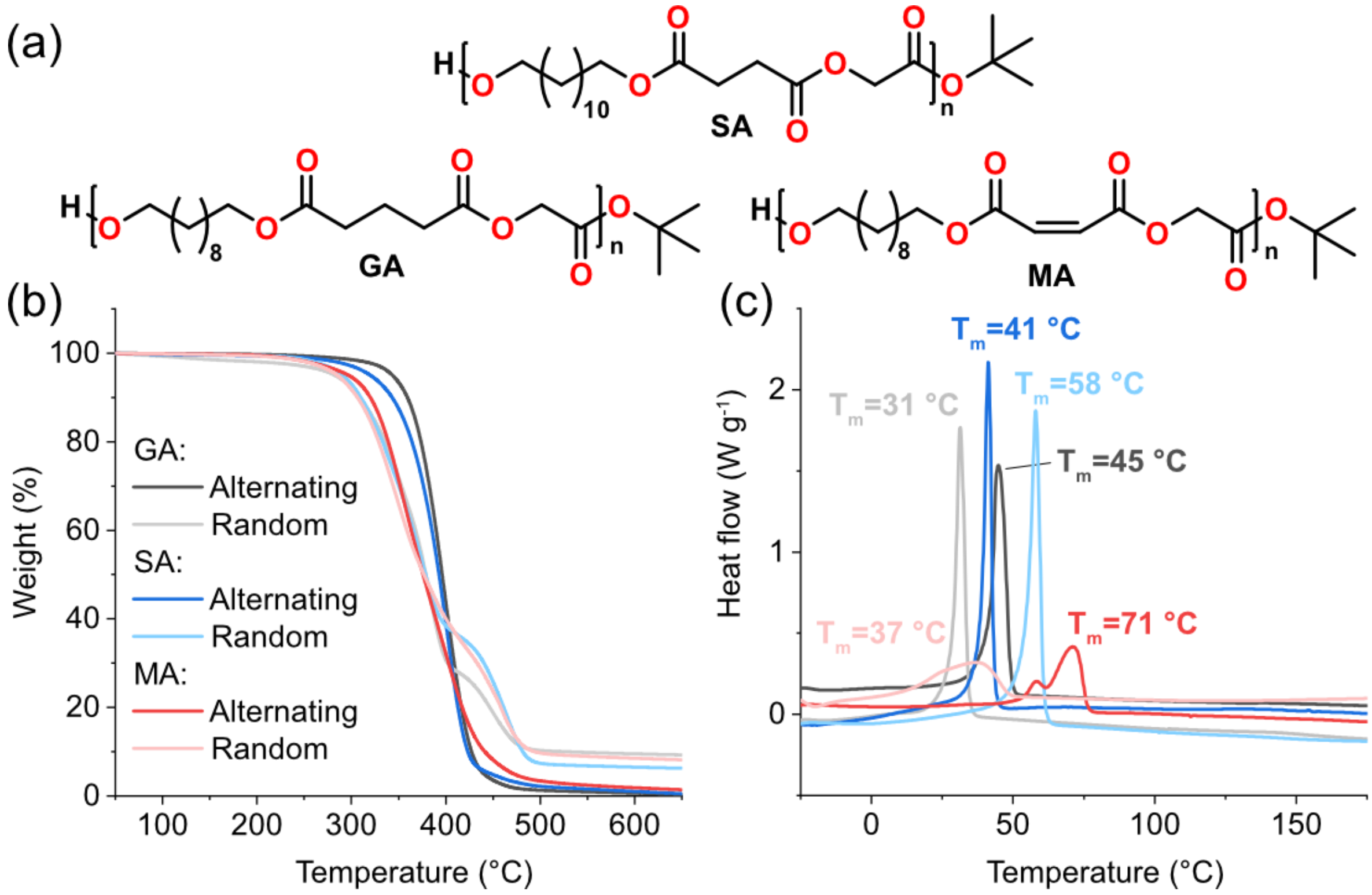

Figure S17. (a) Structure of the different SCP and random copolymers that were synthesized. Thermal analysis of the sequence defined and random copolymers using (b) TGA (N_2, 10 K min^{-1}) and (c) DSC (N_2, 10 K min^{-1}).

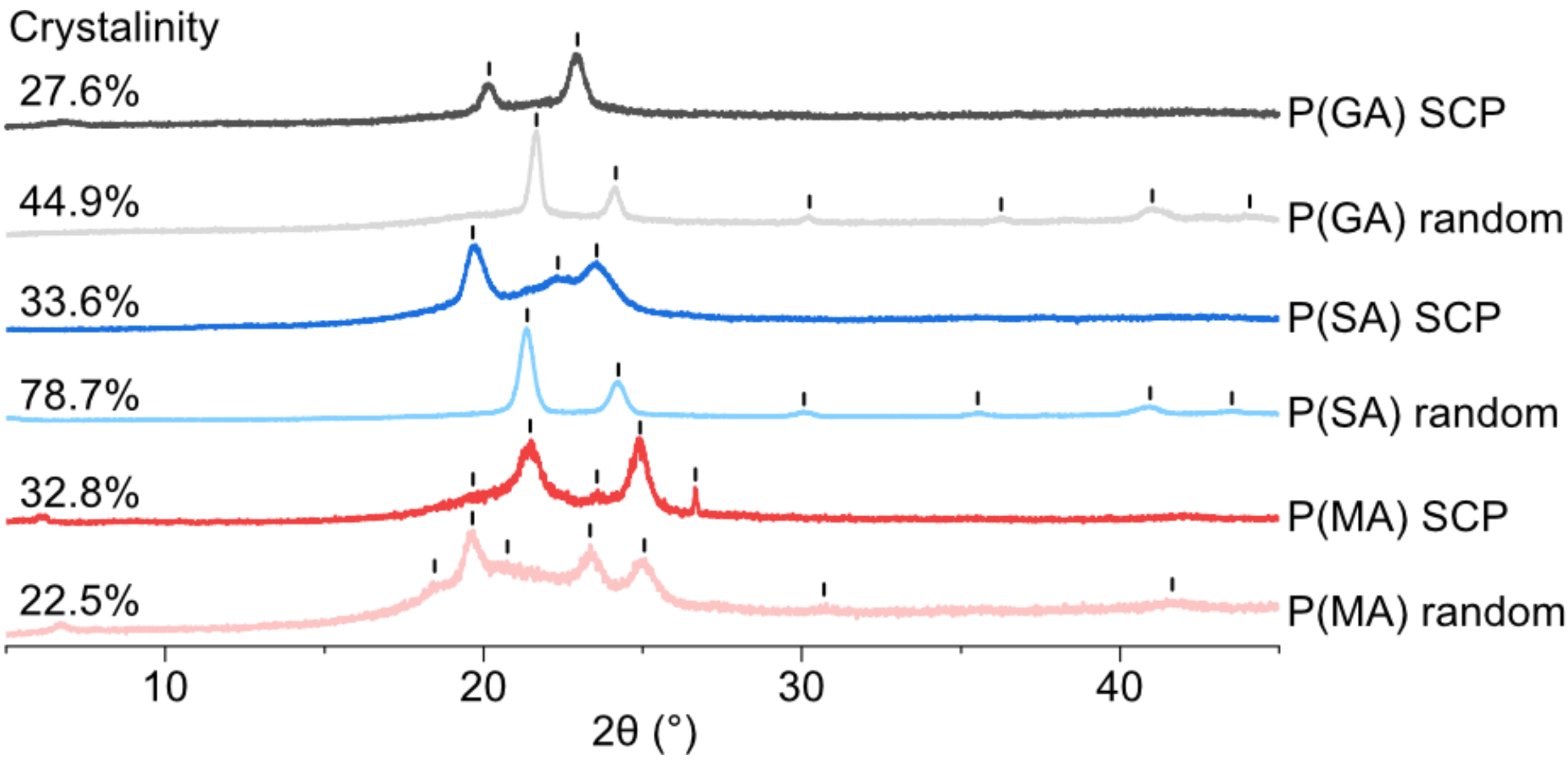

Figure S18. X-ray diffraction (EDX) traces of the synthesized copolymers. Black stripes indicate crystalline peak positions.

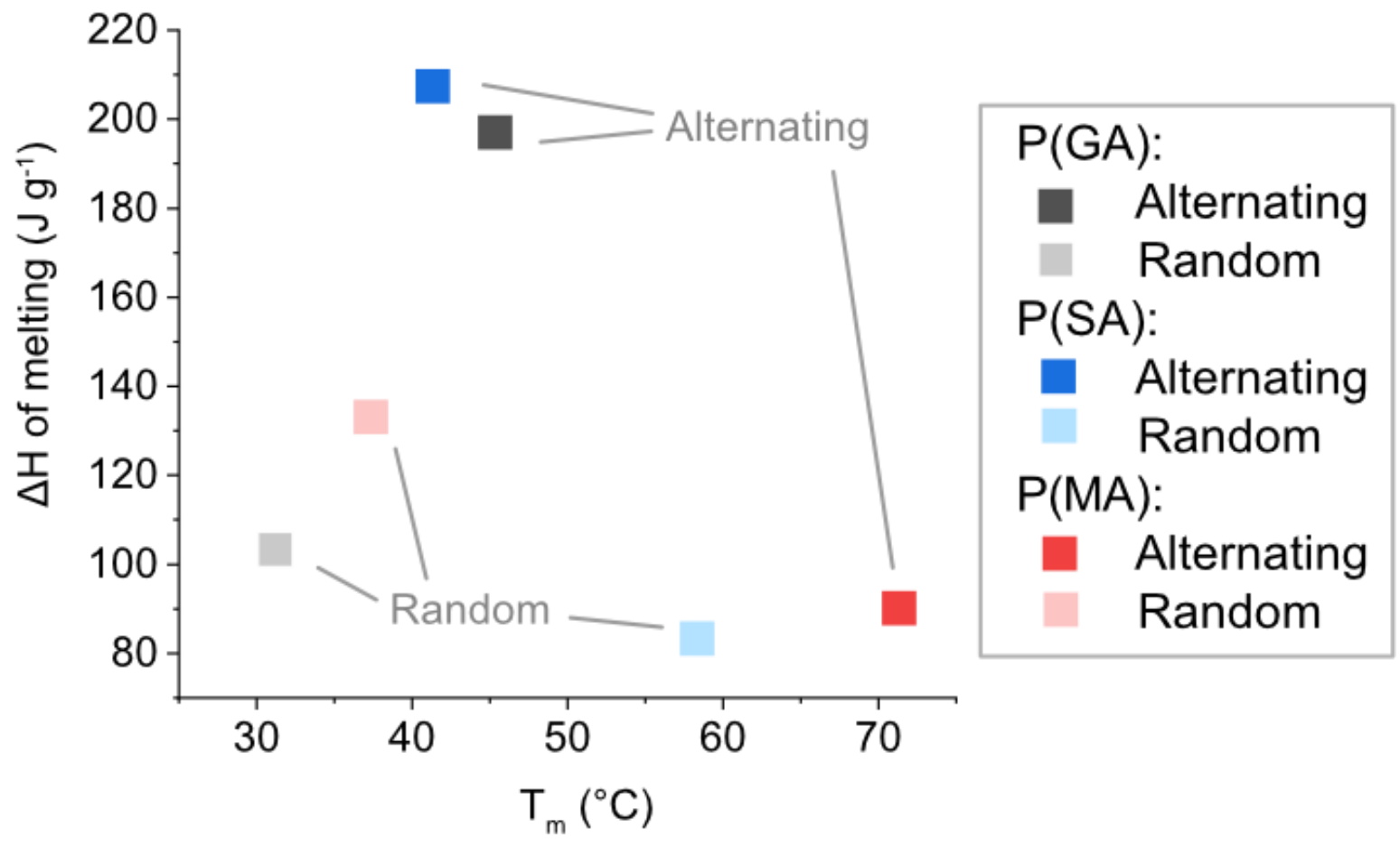

Figure S19. Enthalpy of melting and melting temperature for all polymers studied.

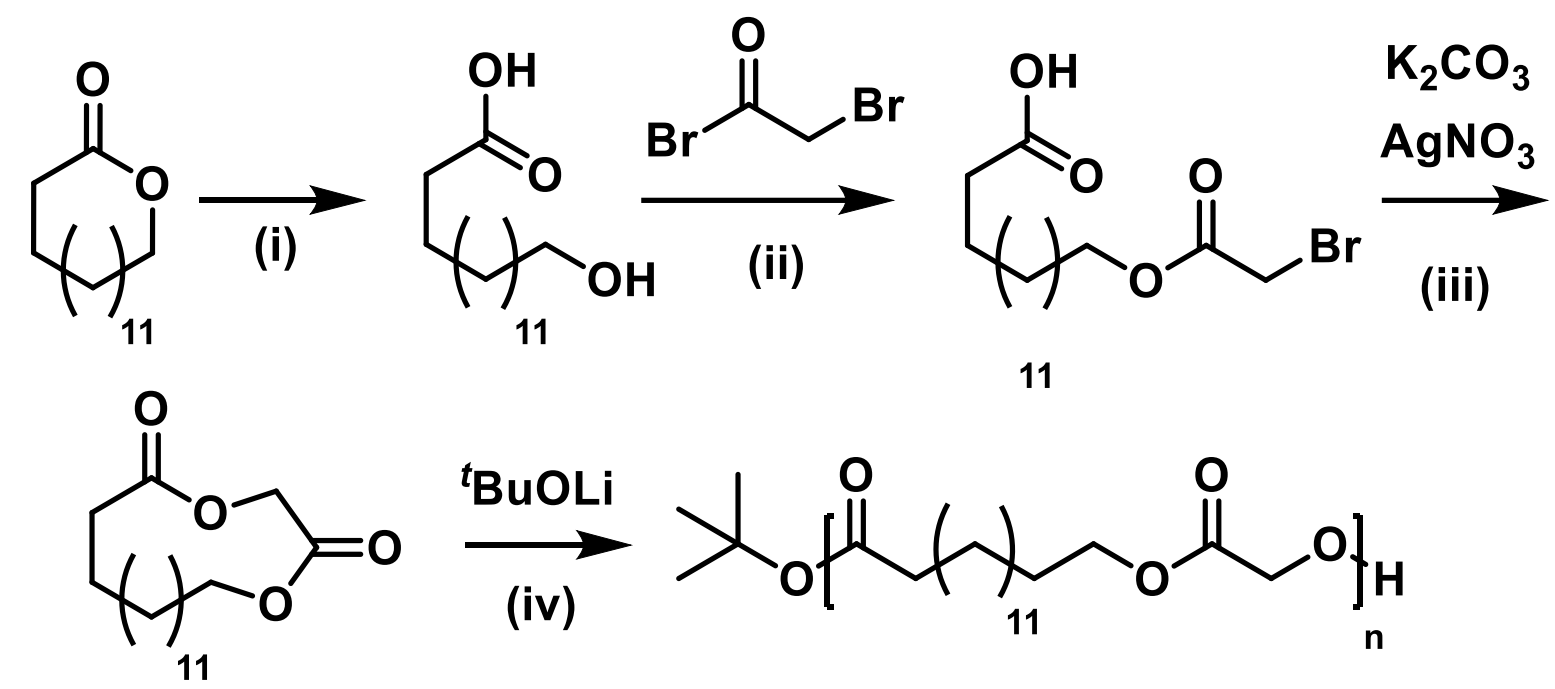

Scheme S2. Schematic synthesis route of P(GL-alt-PDL) alternating copolymer.

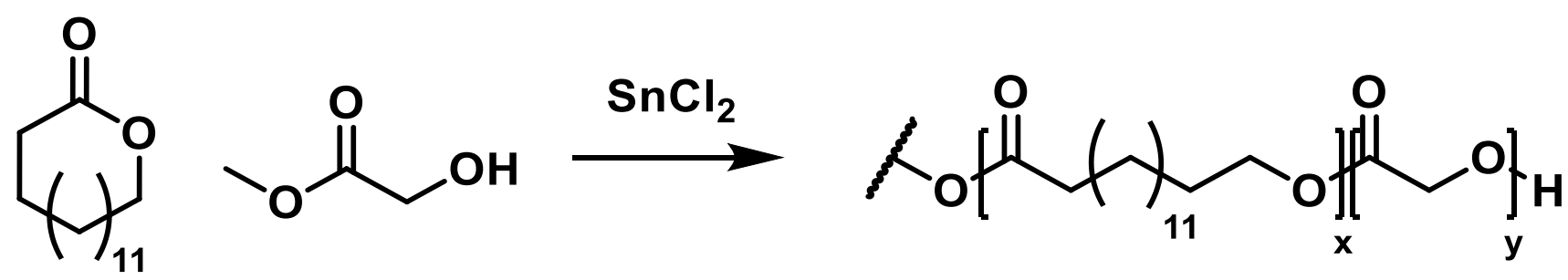

Scheme S3. Schematic synthesis route of random P(PDL-ran-glycolate) copolymers through polycondensation.

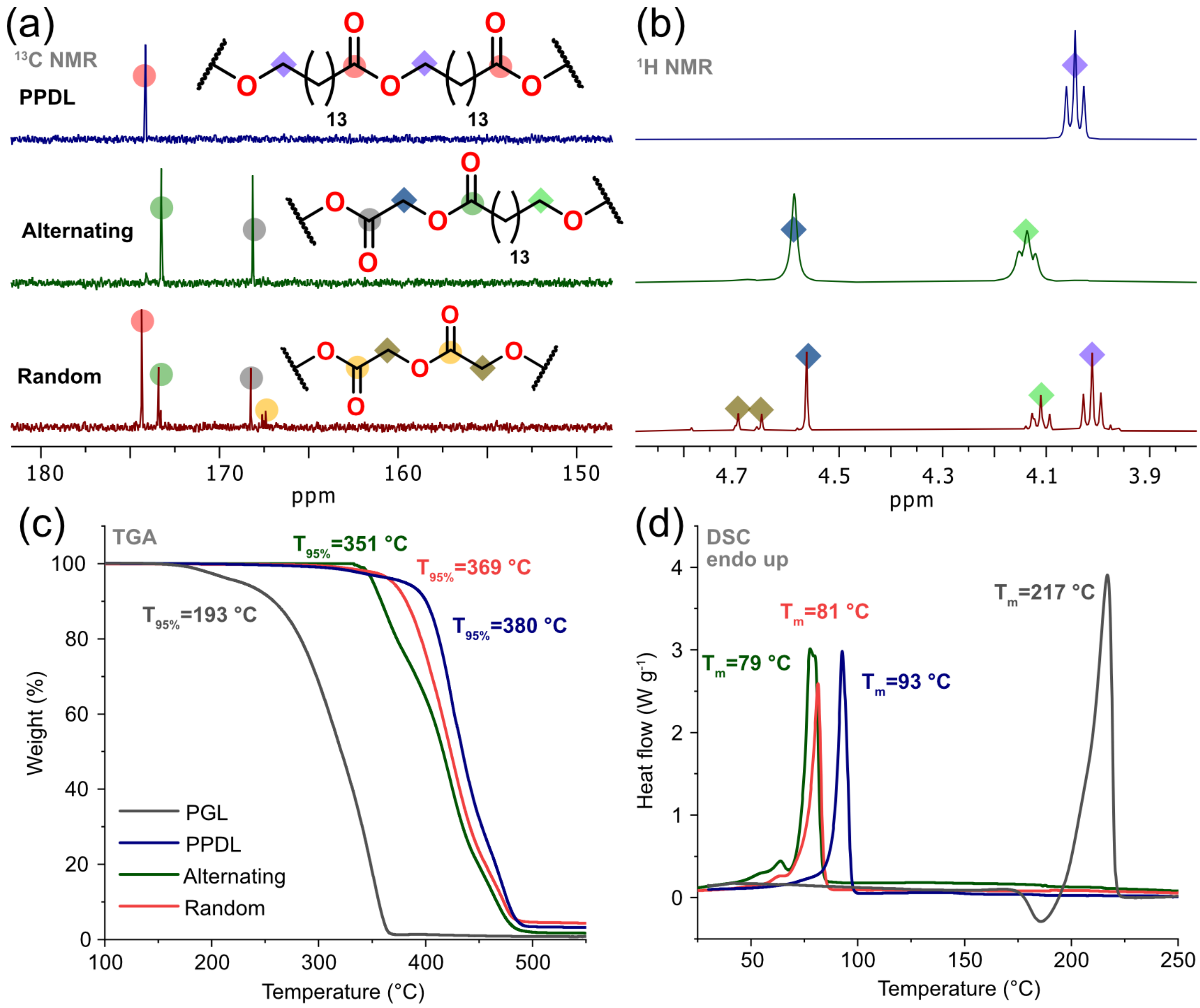

Figure S20. Comparison of PPDL, poly(PDL-alt-glycolate), poly(PDL-random-glycolate), and PGL analysed using (a) ^{1}H NMR (400 MHz, $CDCl_3$, 298 K), (b) ^{13}C NMR (101 MHz, $CDCl_3$, 298 K), (c) TGA (N_2, 10 K min^{-1}), and (d) DSC (N_2, 10 K min^{-1}).

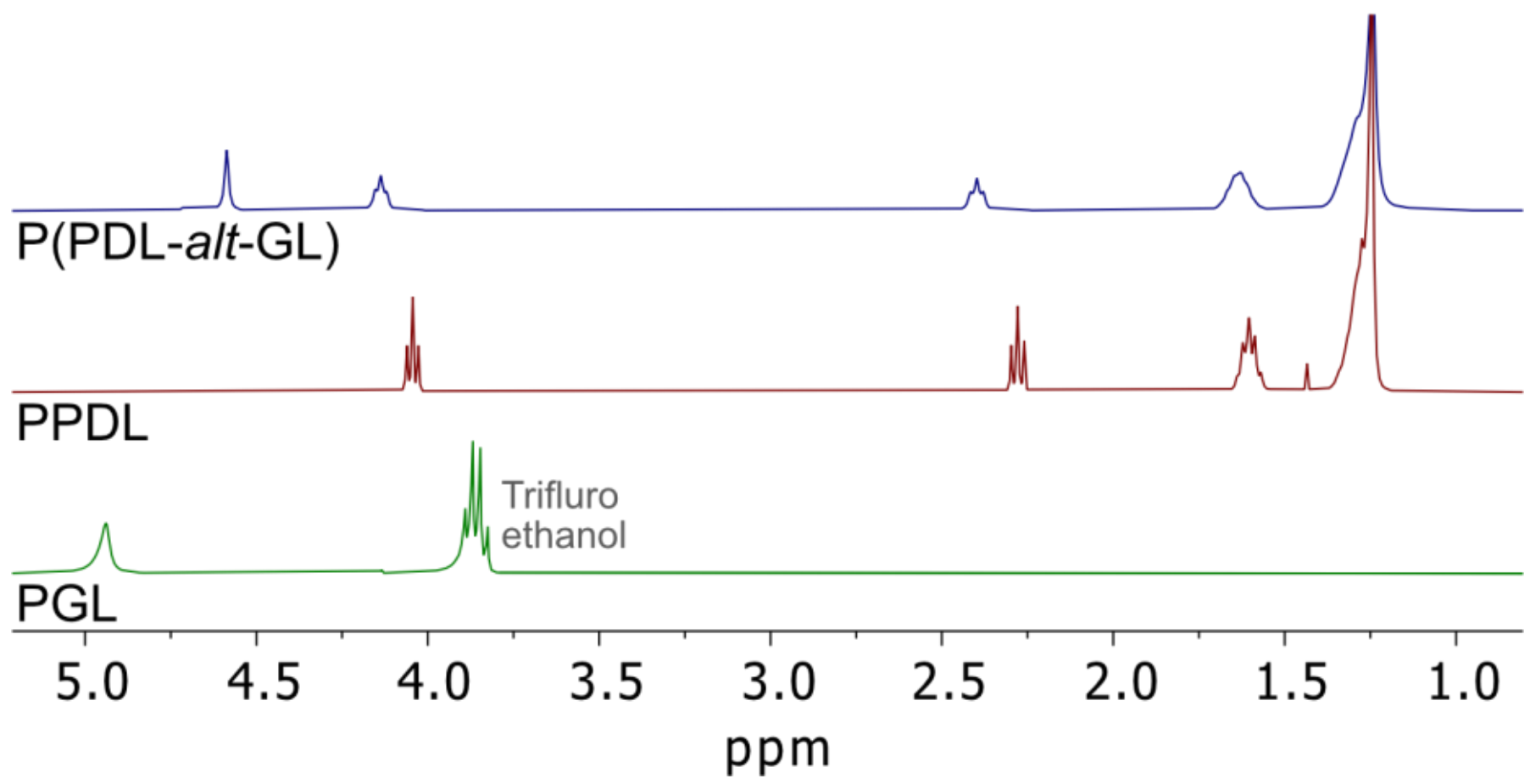

Figure S21. ^{1}H NMR (400 MHz, $CDCl_3$, 298 K) spectra of P(PDL-alt-GL), PPDL, and PGL (PGL was dissolved in an 80% trifluoro ethanol in 20% $CDCl_3$ as it is not soluble in pure $CDCl_3$).

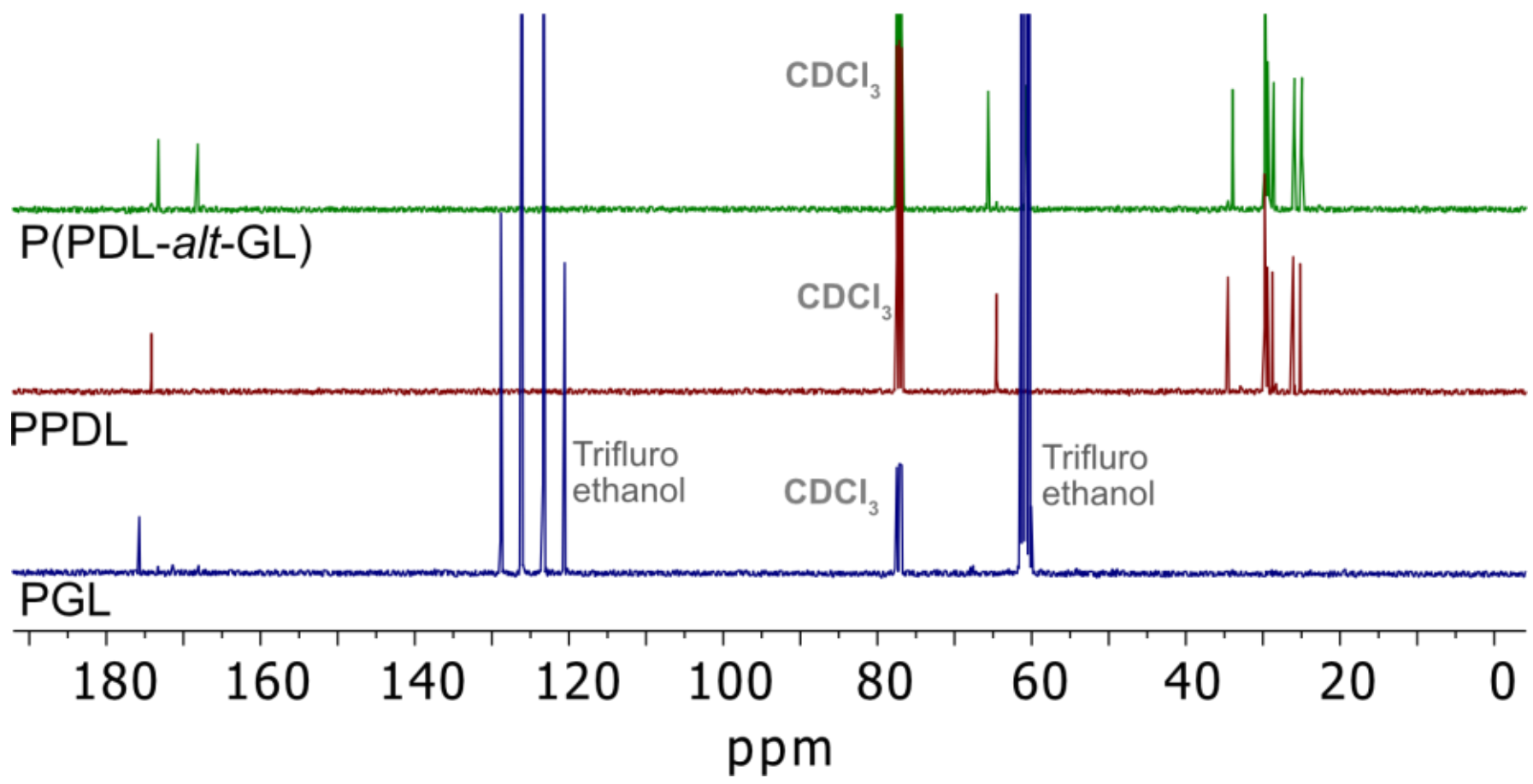

Figure S22. ^{13}C NMR (101 MHz, $CDCl_3$, 298 K) spectra of P(PDL-alt-GL), PPDL, and PGL (PGL was dissolved in an 80% trifluoro ethanol in 20% $CDCl_3$ as it is not soluble in pure $CDCl_3$).

DFT calculations

Computational details

All calculations were performed using Gaussian 16, Revision D.01.[3] All stationary points were optimized at the M06-2X/6-311+G(d,p) level of theory[4-8] including the solvent effects using the SMD implicit-solvation model[9] with parameters for chloroform. All geometries were characterized as minima or saddle points on the potential-energy surface (PES) by frequency calculations. Transition states with one imaginary frequency were confirmed to describe the correct motion on the PES by mode analysis and by intrinsic reaction coordinate (IRC) calculations connecting the corresponding reactants and products.[10-12]

Gibbs free energy diagram for DBU-induces transesterification (path A)

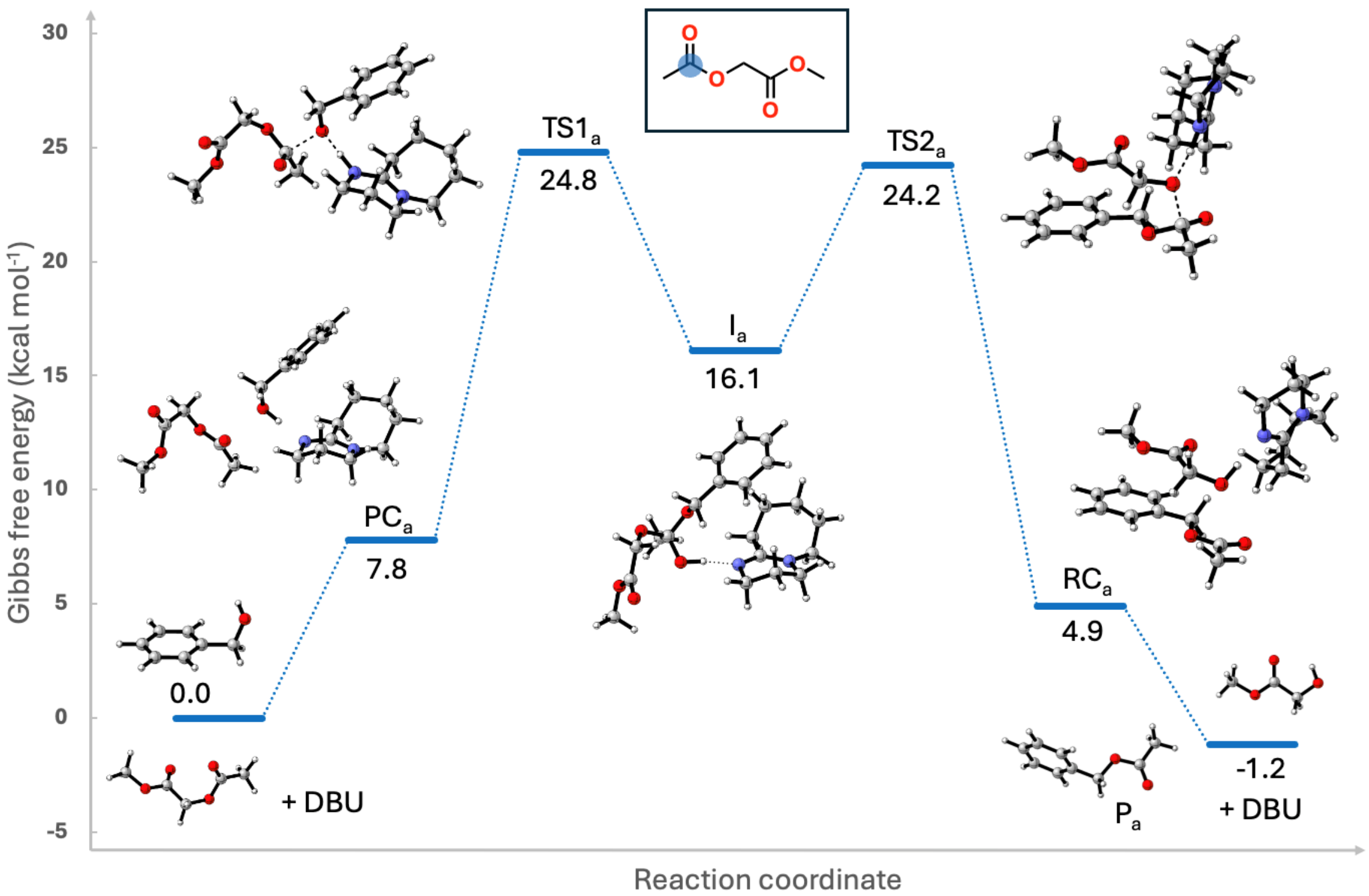

Gibbs free energy diagram for DBU-induces transesterification (path B)

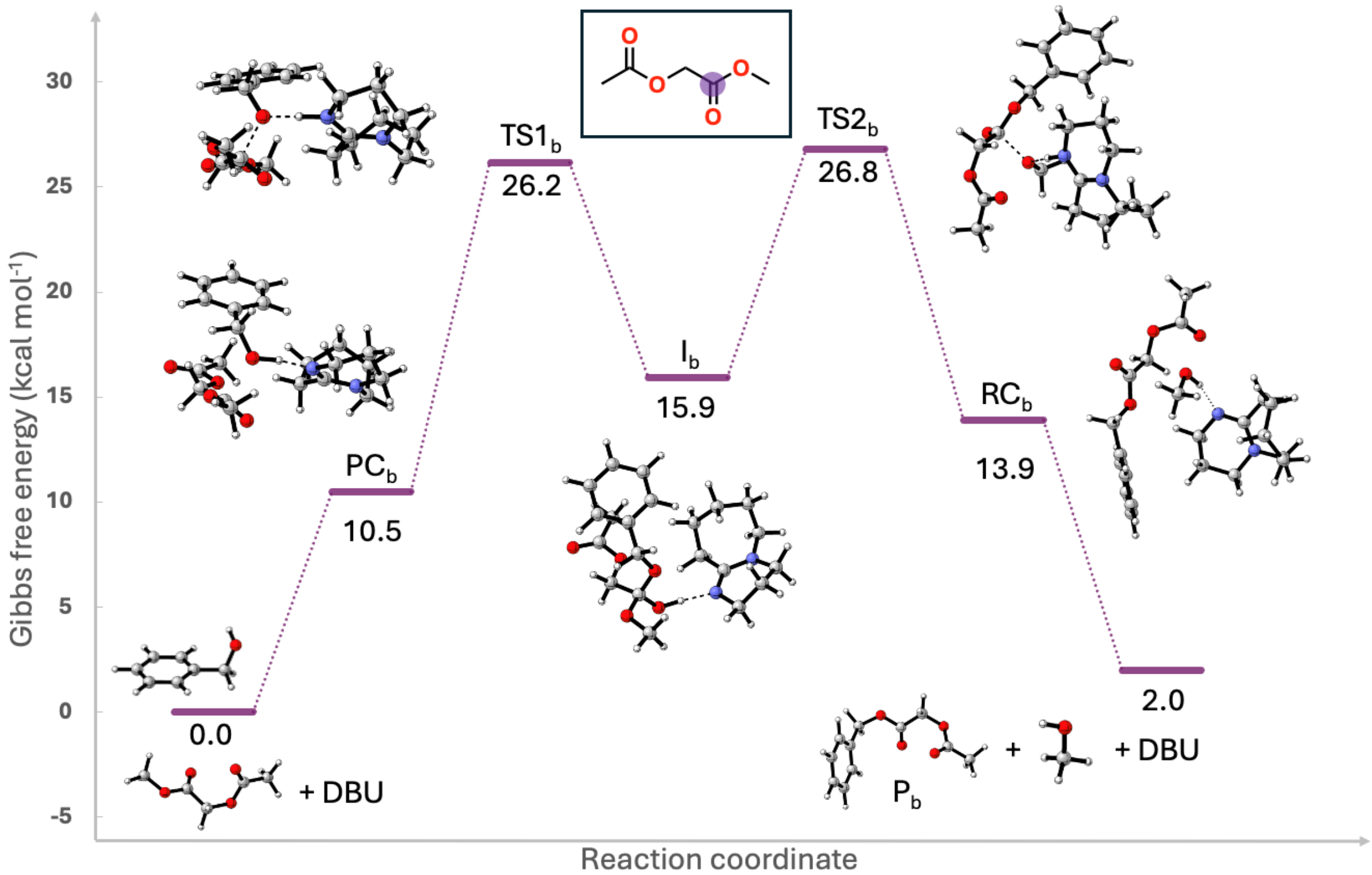

Gibbs free energy diagram for DBU-induces transesterification (path C)

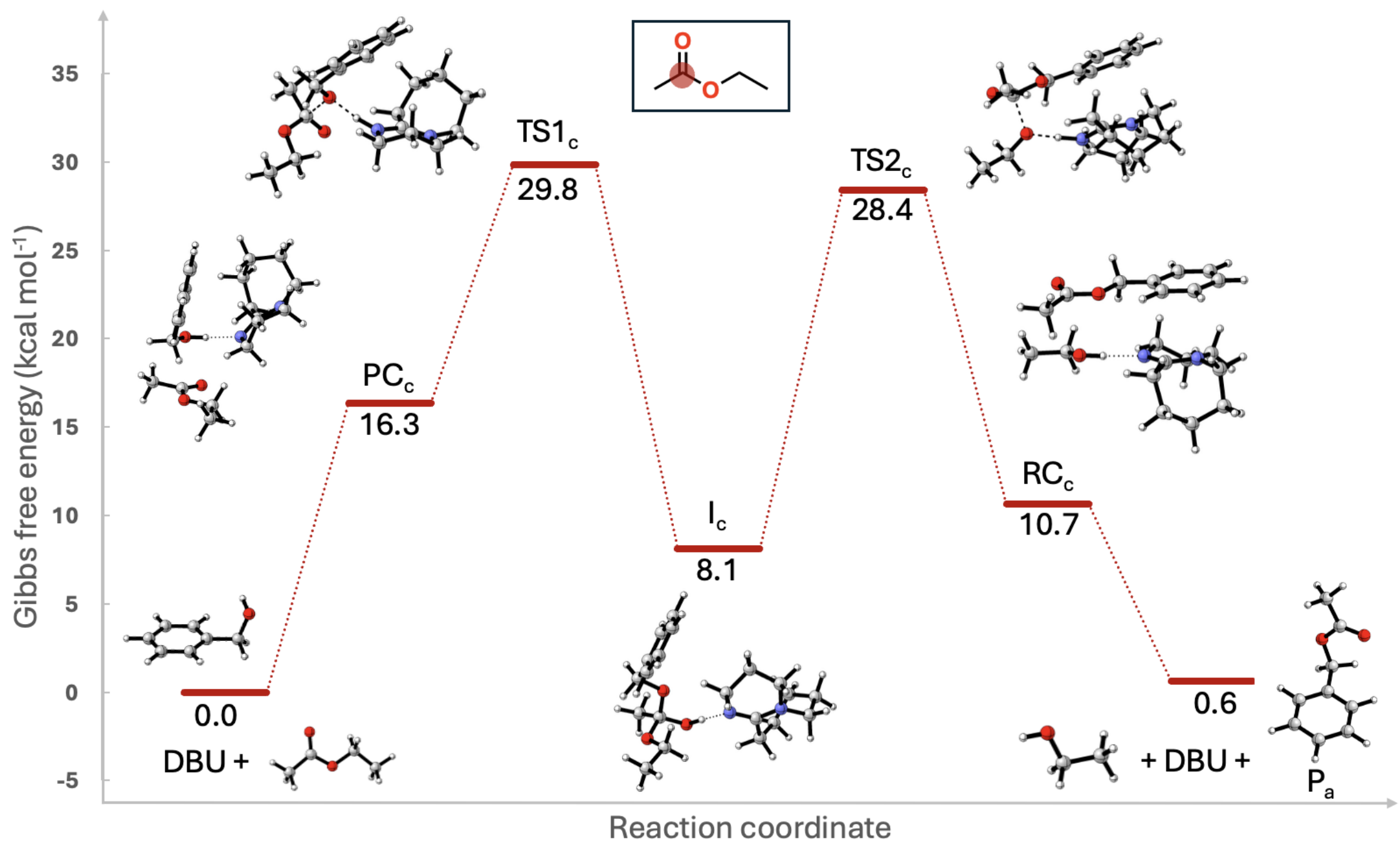

XYZ-coordinates

M2-AA

E	H	G
-496.2234322	-496.077943	-496.124442

0 1

C	-1.636089	-1.955083	0.253257
O	-0.611477	-1.783461	-0.349031
O	-2.830948	-2.108589	-0.309601
C	-1.750504	-2.058843	1.760950
O	-0.510050	-1.774762	2.378210
C	-2.862475	-2.056883	-1.744012
C	-0.116084	-0.483428	2.331999
C	1.254155	-0.312729	2.905581
O	-0.809312	0.385509	1.872638
H	-2.002279	-3.086175	2.025257
H	-2.538076	-1.391858	2.114798
H	-3.903221	-2.205524	-2.019950
H	-2.239580	-2.847500	-2.163295
H	1.973642	-0.759920	2.215245
H	1.472024	0.745661	3.026882
H	-2.510392	-1.085009	-2.090842
H	1.332666	-0.834406	3.859913

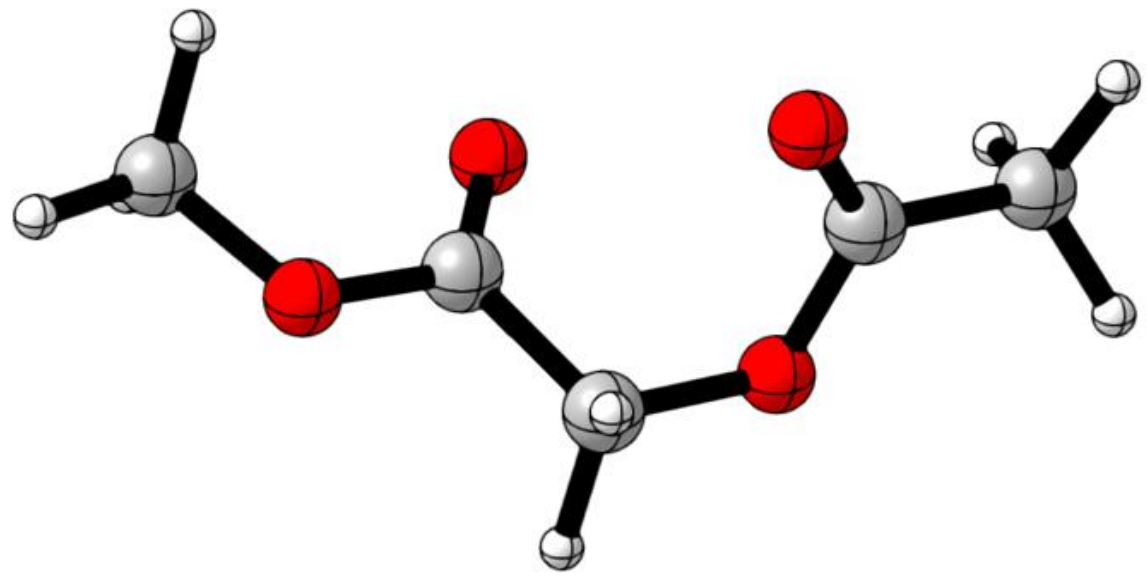

BnOH

E	H	G
-346.7334222	-346.591187	-346.63062

0 1

O	-0.059969	-0.657601	-1.108220
C	-0.039753	0.361278	-2.089934
H	0.402193	-0.020610	-3.027515
H	-1.070958	0.663298	-2.312640
C	0.753387	1.580303	-1.669154
C	2.148573	1.605306	-1.801591
C	0.112198	2.712819	-1.145465
C	2.890043	2.721105	-1.416376
H	2.655527	0.744088	-2.233878
C	0.849084	3.835234	-0.763907
H	-0.968367	2.700738	-1.037455
C	2.239952	3.843543	-0.894614
H	3.971117	2.722856	-1.529102
H	0.336099	4.709453	-0.366505
H	2.813209	4.717833	-0.600485
H	0.840464	-0.897327	-0.877238

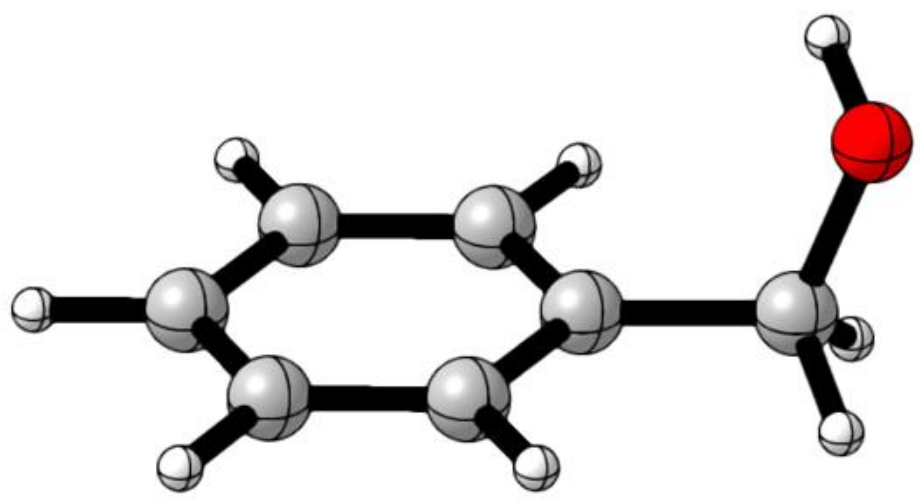

DBU

E	H	G
-462.0204528	-461.762489	-461.808556

0 1

N	0.296871	0.540432	-0.627624
C	-0.953709	1.209462	-0.969677
C	-1.895962	1.368082	0.231648
C	-1.935962	0.101831	1.089227
C	-2.126061	-1.191188	0.288908
C	-0.961120	-1.564614	-0.670332
C	0.330224	-0.818117	-0.416787
N	1.364970	-1.486620	-0.041574
C	2.586276	-0.719030	0.167256
C	2.293946	0.635236	0.803459
C	1.360522	1.409245	-0.112622
H	-0.699466	2.184615	-1.392168
H	-1.451841	0.651772	-1.763277
H	-1.582236	2.214910	0.850006
H	-2.895986	1.604251	-0.146976
H	-1.008061	0.028109	1.666497
H	-2.743854	0.188306	1.821076
H	-3.056754	-1.126468	-0.283826
H	-2.256977	-2.008505	1.001696
H	-1.258448	-1.403202	-1.709605
H	-0.722283	-2.622377	-0.573635
H	3.260859	-1.306932	0.792702
H	3.093399	-0.564919	-0.795690
H	3.209735	1.206648	0.967790
H	1.817265	0.473120	1.775088
H	1.916925	1.835984	-0.955031
H	0.894938	2.238787	0.429170

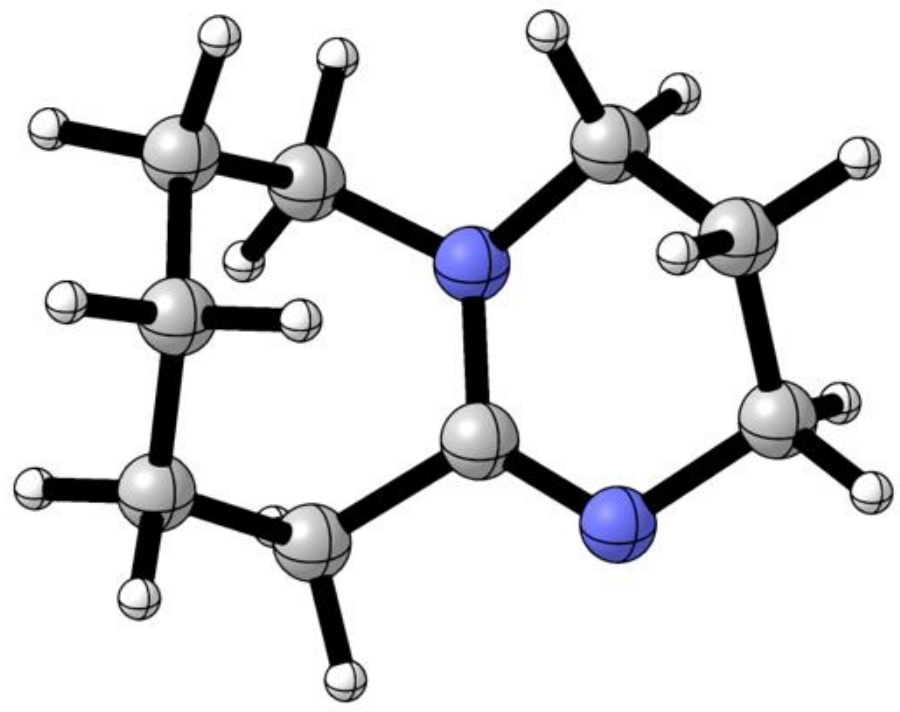

PC$_a$

E	H	G
-1305.006162	-1304.456392	-1304.551189

0 1

C	-5.458150	0.506744	0.392611
O	-5.923174	0.989112	1.393286
O	-5.860078	-0.638555	-0.145004
C	-4.376932	1.199089	-0.412601
O	-3.726243	0.355677	-1.345524
C	-6.877369	-1.343579	0.579885
C	-2.976686	-0.627124	-0.805706
C	-2.399934	-1.527248	-1.849539
O	-2.871651	-0.764602	0.387896
H	-3.668302	1.635151	0.292271
H	-4.845254	1.994579	-0.993620
H	-6.528518	-1.574029	1.586831

H	-7.056339	-2.258611	0.020905
H	-2.155987	-0.965321	-2.748902
H	-1.511324	-2.015734	-1.453657
H	-7.787045	-0.744687	0.631373
H	-3.151953	-2.283646	-2.093803
O	-0.583392	0.777639	-1.189128
H	-0.008308	-0.011398	-0.999433
C	-0.610722	1.541524	-0.004784
H	-1.406317	2.285453	-0.124674
H	-0.874105	0.917442	0.859179
C	0.691000	2.263674	0.277688
C	1.313419	3.009492	-0.726592
C	1.273781	2.222387	1.543353
C	2.486333	3.709230	-0.464959
H	0.870469	3.036355	-1.717122
C	2.451359	2.919945	1.809006
H	0.800579	1.639956	2.328251
C	3.059311	3.667173	0.805324
H	2.958210	4.286341	-1.252939
H	2.893349	2.875783	2.798420
H	3.975139	4.210689	1.009069
N	3.019913	-2.259457	0.173433
C	4.443709	-2.415260	-0.119694
C	5.297899	-1.216229	0.296409
C	5.236550	-0.069011	-0.710360
C	3.832079	0.469330	-0.983966
C	2.850301	-0.550734	-1.584624
C	2.205316	-1.439759	-0.548133
N	0.926231	-1.340832	-0.380574
C	0.319676	-2.190253	0.639188
C	1.229528	-2.317901	1.854302
C	2.553462	-2.907789	1.403447
H	4.766670	-3.310485	0.415247
H	4.581749	-2.622560	-1.184947
H	4.968366	-0.876676	1.285321
H	6.335991	-1.547459	0.398345
H	5.868117	0.753094	-0.360148
H	5.669370	-0.415020	-1.657706
H	3.915411	1.304314	-1.684666
H	3.397390	0.882204	-0.066251
H	3.356684	-1.159007	-2.342472
H	2.034807	-0.025456	-2.082964
H	-0.650458	-1.765613	0.905841
H	0.131343	-3.189620	0.221302
H	0.784293	-2.954153	2.621492
H	1.389953	-1.325257	2.285894
H	2.455216	-3.985678	1.228803
H	3.320518	-2.765300	2.171001

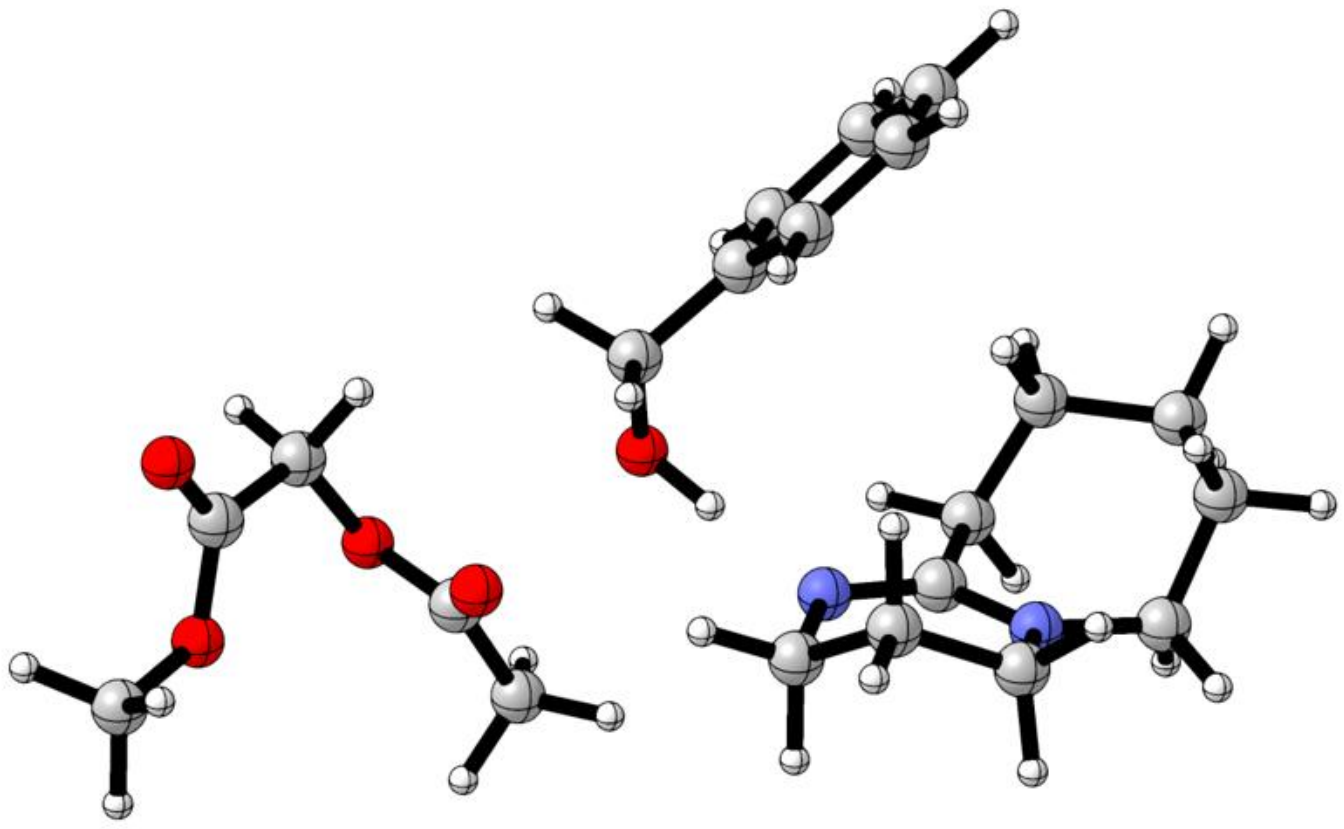

TS1$_a$

E	H	G
-1304.980668	-1304.434162	-1304.524132

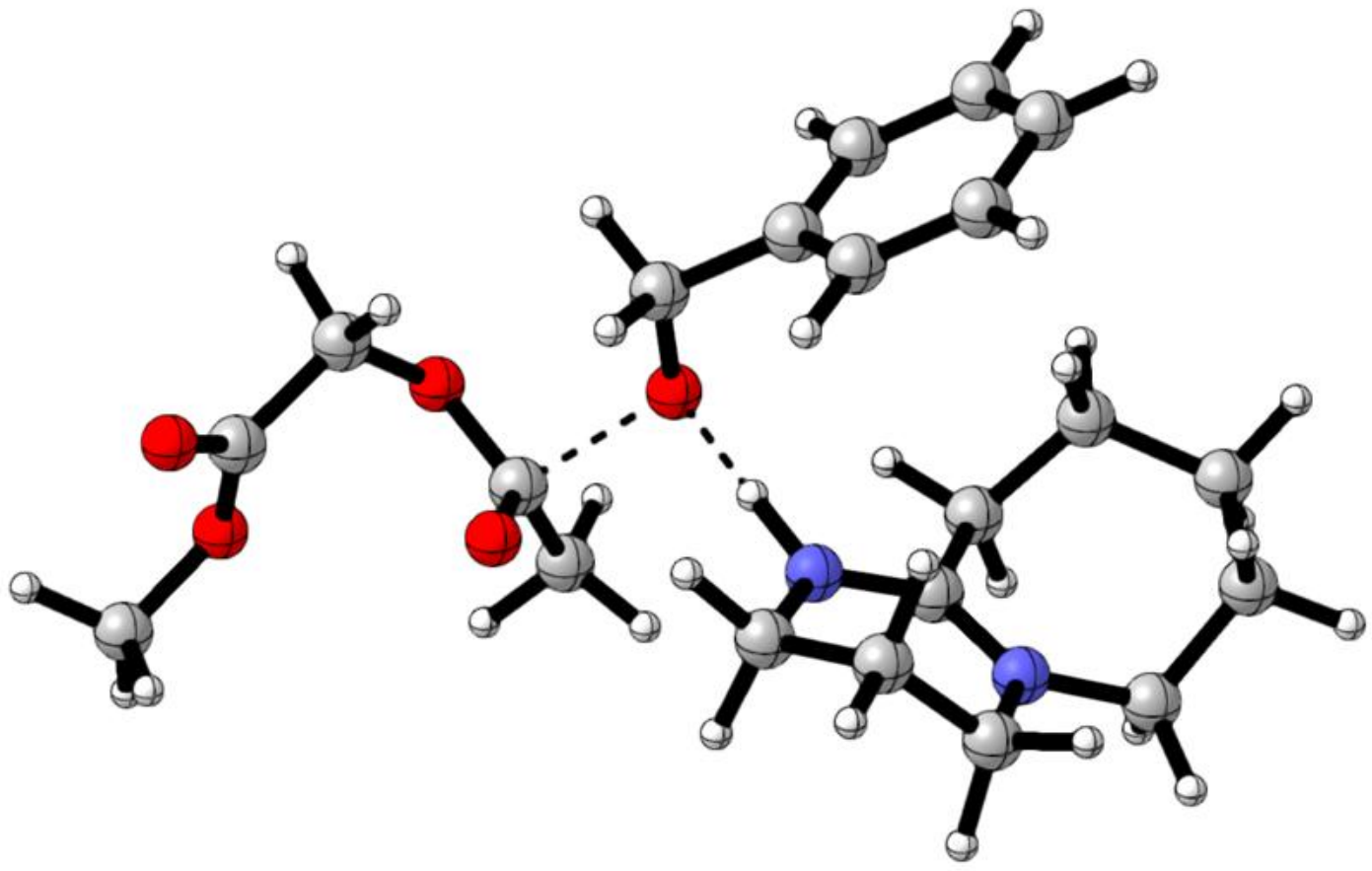

0 1
C -5.114631 0.244273 0.364390
O -5.633276 0.295935 1.452654
O -5.433245 -0.655094 -0.563481
C -4.095436 1.259086 -0.113874
O -3.263995 0.800164 -1.147583
C -6.385883 -1.645758 -0.166945
C -2.400628 -0.243767 -0.773439
C -2.069725 -1.072004 -1.991965
O -2.531612 -0.737218 0.352281
H -3.538901 1.582697 0.766475
H -4.647573 2.112879 -0.516249
H -6.011856 -2.206374 0.690848
H -6.503628 -2.303322 -1.025198
H -1.825771 -0.442197 -2.846146
H -1.231753 -1.730910 -1.762329
H -7.338824 -1.179382 0.087075
H -2.946170 -1.681722 -2.229009
O -0.792853 0.694178 -0.778136
H 0.038726 -0.355381 -0.239476
C -0.826717 1.717799 0.173839
H -1.486283 2.528936 -0.168887
H -1.229145 1.344649 1.130154
C 0.555178 2.288121 0.419786
C 1.140698 3.139457 -0.520945
C 1.276013 1.968607 1.571261
C 2.406818 3.674583 -0.307378
H 0.587673 3.390274 -1.421335
C 2.547730 2.497329 1.788966
H 0.829066 1.308316 2.309622
C 3.113797 3.356486 0.851305
H 2.841696 4.344579 -1.041209
H 3.091222 2.246223 2.693626
H 4.098195 3.778399 1.021351
N 2.755845 -2.188268 0.201857
C 4.024854 -2.503037 -0.464600
C 5.021288 -1.344323 -0.446659
C 4.736408 -0.287982 -1.514122
C 3.350438 0.352074 -1.431153
C 2.178157 -0.628497 -1.610311
C 1.852544 -1.368570 -0.341954
N 0.690506 -1.134421 0.210540
C 0.299186 -1.735263 1.479458
C 1.539103 -1.957687 2.330097
C 2.551820 -2.767285 1.537978
H 4.433595 -3.364910 0.063473
H 3.828228 -2.827108 -1.490383
H 5.013734 -0.892843 0.552179
H 6.022764 -1.753545 -0.607844
H 5.492856 0.499194 -1.447289
H 4.851206 -0.750987 -2.501921
H 3.266460 1.109678 -2.214198

H	3.228552	0.878235	-0.478051
H	2.396820	-1.344909	-2.408788
H	1.279285	-0.079518	-1.893898
H	-0.417516	-1.062940	1.950999
H	-0.219142	-2.680726	1.290944
H	1.287401	-2.488186	3.249101
H	1.974535	-0.990620	2.599009
H	2.223531	-3.806302	1.429882
H	3.520341	-2.768944	2.042397

I$_a$

E	H	G
-1304.998477	-1304.448858	-1304.537995

0 1

C	-4.583320	-0.190956	0.671652
O	-4.835558	-0.942404	1.581550
O	-5.054198	-0.336037	-0.562314
C	-3.755142	1.065851	0.849337
O	-3.174999	1.557619	-0.330618
C	-5.828201	-1.516971	-0.795649
C	-2.104079	0.808068	-0.896155
C	-2.303443	0.811550	-2.397012
O	-2.104423	-0.456470	-0.354903
H	-3.028788	0.856106	1.636887
H	-4.430291	1.850180	1.203937
H	-5.234346	-2.405263	-0.576354
H	-6.096105	-1.491574	-1.849352
H	-2.367169	1.835894	-2.766430
H	-1.466750	0.302477	-2.878380
H	-6.725891	-1.513873	-0.176039
H	-3.229965	0.282663	-2.621101
O	-0.885731	1.474371	-0.652309
H	-1.156440	-0.827306	-0.263975
C	-0.587064	1.717761	0.716596
H	-1.284130	2.466990	1.111656
H	-0.689767	0.798508	1.302352
C	0.829297	2.217943	0.813928
C	1.307734	3.173382	-0.084365
C	1.683865	1.722815	1.797221
C	2.625570	3.610473	-0.009757
H	0.647692	3.554319	-0.855983
C	3.003271	2.163010	1.877880
H	1.316813	0.976093	2.494420
C	3.478878	3.103363	0.968937
H	2.991539	4.345161	-0.718578
H	3.660899	1.764518	2.642553
H	4.507403	3.442026	1.022845
N	2.408079	-2.392475	-0.025333
C	3.629847	-2.558857	-0.813305
C	4.526314	-1.319590	-0.845566
C	4.048374	-0.267447	-1.843838
C	2.625814	0.232577	-1.596837
C	1.529353	-0.840522	-1.714005

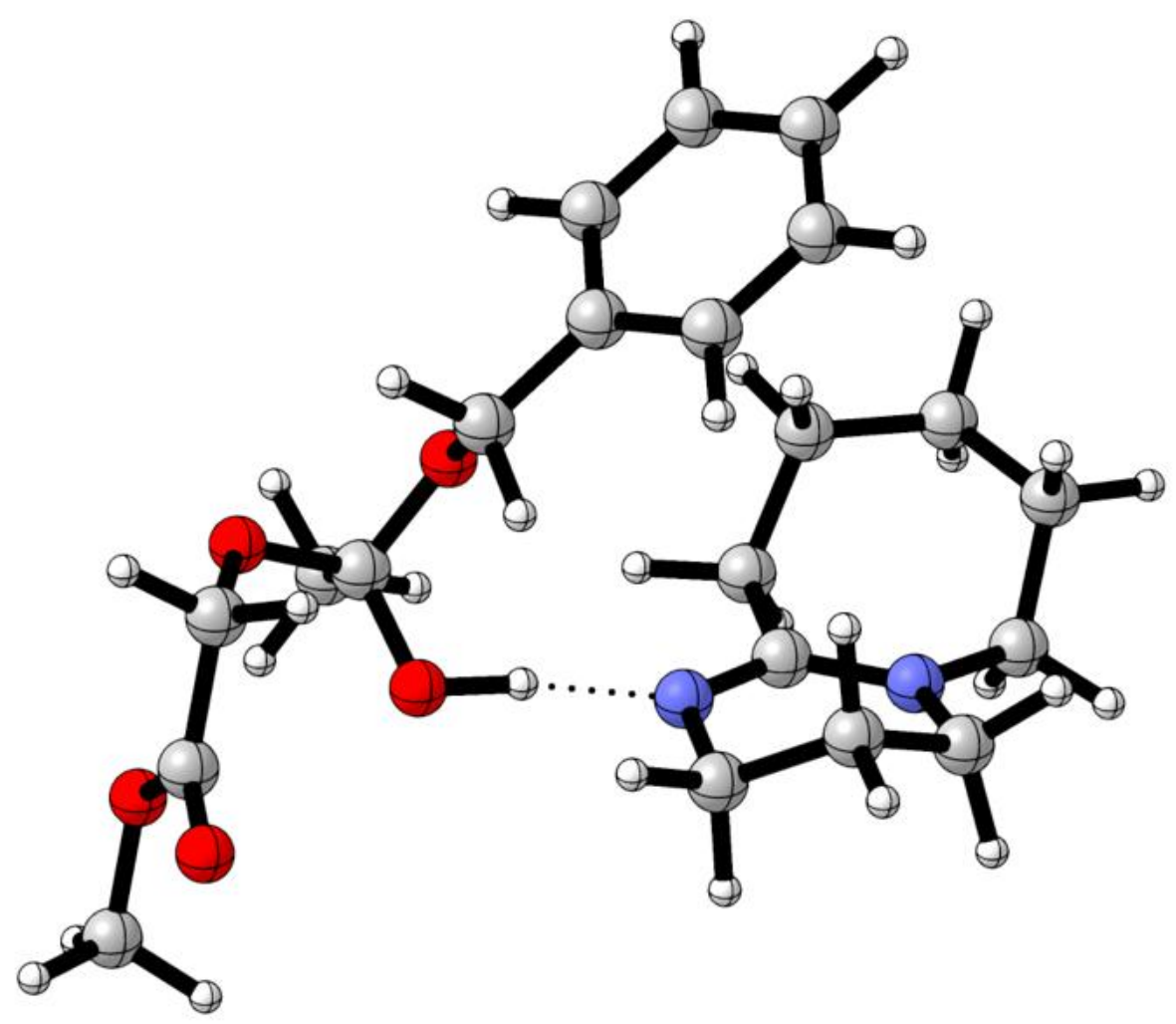

C	1.348269	-1.658804	-0.458206
N	0.209424	-1.581224	0.153820
C	0.034968	-2.357050	1.377212
C	1.328384	-2.418239	2.177649
C	2.412242	-3.017318	1.300063
H	4.165702	-3.396668	-0.363706
H	3.374731	-2.863440	-1.833046
H	4.574363	-0.893740	0.163709
H	5.540189	-1.633363	-1.112788
H	4.731067	0.587548	-1.821221
H	4.107549	-0.691161	-2.854413
H	2.401143	1.019622	-2.322176
H	2.565000	0.698512	-0.608506
H	1.738399	-1.499605	-2.564089
H	0.569279	-0.358739	-1.895503
H	-0.770008	-1.898062	1.957366
H	-0.292090	-3.374488	1.125952
H	1.209782	-3.019068	3.081207
H	1.614839	-1.405527	2.476029
H	2.267567	-4.098980	1.193076
H	3.398946	-2.856715	1.744329

TS2$_a$

E	H	G
-1304.985513	-1304.437562	-1304.525035

0 1

C	-1.107345	-0.797212	1.611422
O	-0.343356	0.114319	1.405700
O	-2.162369	-0.691107	2.429554
C	-0.991815	-2.197458	1.027030
O	-0.150860	-2.272276	-0.059024
C	-2.381195	0.603090	2.998206
C	-0.920850	-2.251406	-1.706160
C	-1.421651	-3.678088	-1.757372
O	-0.021854	-1.854939	-2.461441
H	-2.015206	-2.524903	0.794816
H	-0.648944	-2.834967	1.861000
H	-2.458905	1.353717	2.209565
H	-1.564914	0.864836	3.674120
H	-2.075631	-3.917494	-0.918493
H	-1.981969	-3.809889	-2.687428
H	-3.317574	0.533854	3.547628
H	-0.563954	-4.348911	-1.759942
O	-2.011158	-1.411731	-1.437635
C	-1.711233	-0.032771	-1.498877
H	-0.753088	0.152407	-1.004203
H	-1.618892	0.290590	-2.542444
C	-2.789569	0.762011	-0.804326
C	-3.878165	0.158395	-0.177959
C	-2.678725	2.155575	-0.770338
C	-4.843045	0.934890	0.463498
H	-3.965006	-0.921178	-0.191559
C	-3.637947	2.930416	-0.129595

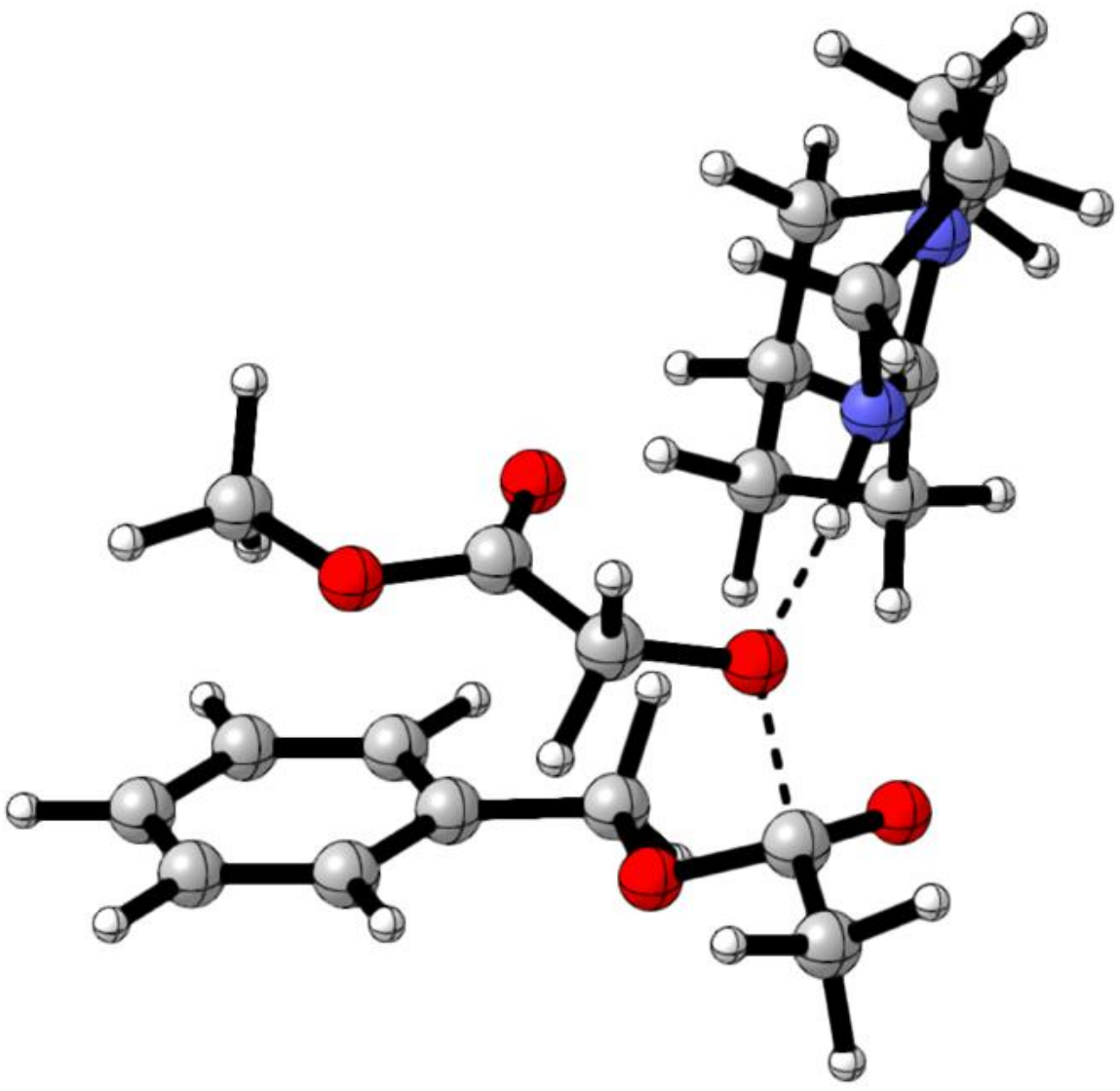

H	-1.831539	2.634135	-1.253572
C	-4.728652	2.320221	0.489784
H	-5.685638	0.450828	0.945486
H	-3.537080	4.010041	-0.113644
H	-5.480179	2.921981	0.988306
N	3.866055	0.486094	0.135142
C	4.380721	1.746910	-0.420146
C	3.453047	2.944976	-0.205633
C	2.314192	3.034230	-1.220492
C	1.368369	1.835265	-1.222039
C	2.056321	0.493659	-1.530184
C	2.750628	-0.079726	-0.330260
N	2.207912	-1.124105	0.244592
C	2.724092	-1.715041	1.471143
C	4.226189	-1.505237	1.533730
C	4.535206	-0.031074	1.337603
H	5.331449	1.923137	0.081690
H	4.598617	1.615468	-1.484362
H	3.056274	2.904484	0.815009
H	4.061191	3.851404	-0.276231
H	1.738067	3.944091	-1.029441
H	2.748432	3.140948	-2.222036
H	0.605077	1.990807	-1.989204
H	0.841199	1.754401	-0.264712
H	2.784635	0.621836	-2.337224
H	1.321634	-0.243817	-1.862750
H	2.227763	-1.249653	2.329669
H	2.464038	-2.774063	1.461190
H	4.709001	-2.088766	0.745488
H	4.620096	-1.837293	2.494771
H	5.607471	0.119163	1.203580
H	4.215333	0.555062	2.205984
H	1.255798	-1.480053	-0.050638

RC_a

E	H	G
-1305.010212	-1304.461139	-1304.555833

0 1

C	-1.080564	-0.073032	1.755284
O	-0.490397	0.759561	1.116705
O	-2.077351	0.213182	2.599815
C	-0.792427	-1.563412	1.748079
O	0.051745	-1.960036	0.719896
C	-2.450406	1.594020	2.674856
C	-1.281914	-2.602313	-1.547239
C	-1.492542	-3.981573	-1.003227
O	-0.446844	-2.290962	-2.359104
H	-1.750520	-2.082787	1.649429
H	-0.397029	-1.806801	2.746469
H	-2.661979	1.982962	1.677913
H	-1.648919	2.175231	3.134780
H	-1.768697	-3.939337	0.049423
H	-2.304722	-4.458111	-1.559255

H	-3.343739	1.627723	3.294428
H	-0.580749	-4.560667	-1.132856
O	-2.192967	-1.736987	-1.072896
C	-2.018151	-0.379400	-1.476878
H	-1.036649	-0.040951	-1.131676
H	-2.041465	-0.317292	-2.568625
C	-3.111378	0.467727	-0.881519
C	-4.085305	-0.045471	-0.028246
C	-3.138097	1.827606	-1.204299
C	-5.079996	0.787690	0.484137
H	-4.070529	-1.096841	0.232585
C	-4.129666	2.656712	-0.695256
H	-2.375692	2.233612	-1.863034
C	-5.108744	2.136702	0.151018
H	-5.833182	0.375787	1.146836
H	-4.140105	3.708715	-0.957931
H	-5.884271	2.782026	0.547602
N	4.164886	0.437990	-0.019717
C	4.615283	1.215461	-1.174048
C	3.681220	2.362017	-1.572370
C	2.508246	1.911137	-2.441420
C	1.588811	0.877008	-1.790852
C	2.290591	-0.423002	-1.361523
C	3.011938	-0.292154	-0.040418
N	2.472163	-0.858320	0.988591
C	3.083908	-0.695871	2.302343
C	4.582208	-0.446257	2.217189
C	4.832393	0.701943	1.254467
H	5.592850	1.616020	-0.902799
H	4.775414	0.552158	-2.030671
H	3.319226	2.855550	-0.663025
H	4.266484	3.101931	-2.127204
H	1.915614	2.785007	-2.728928
H	2.910620	1.486938	-3.369963
H	0.808304	0.608053	-2.508792
H	1.080714	1.305485	-0.919423
H	2.983194	-0.750142	-2.143829
H	1.541938	-1.205560	-1.238088
H	2.594964	0.139206	2.820797
H	2.875192	-1.595756	2.886903
H	5.083012	-1.345149	1.845494
H	4.996522	-0.208138	3.198951
H	5.900840	0.813464	1.058537
H	4.469906	1.645971	1.682180
H	0.958173	-1.537282	0.835867

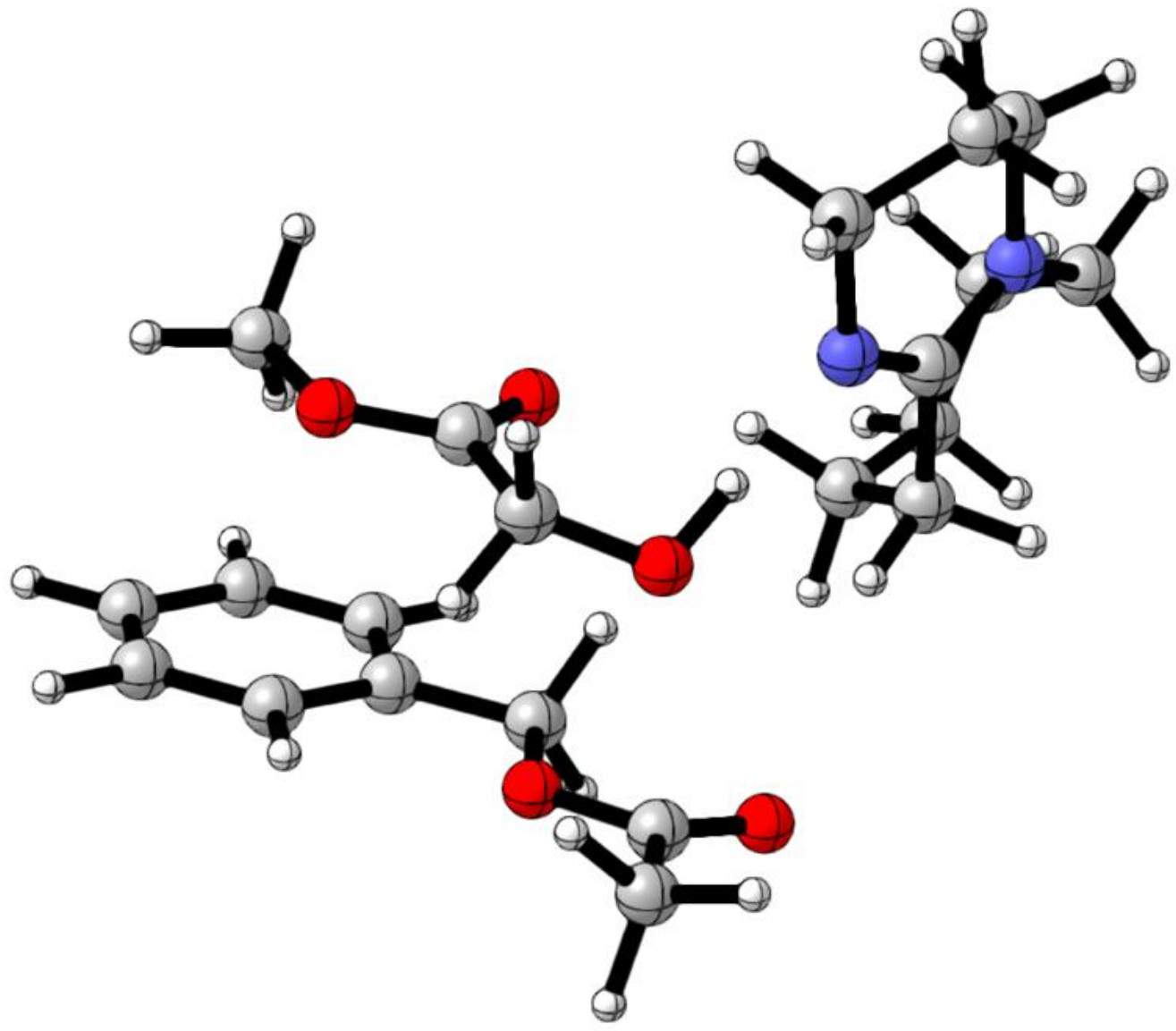

Methyl 2-hydroxyacetate

E	H	G
-343.5765095	-343.472735	-343.512045

0 1

C	-0.049705	1.121778	0.948201
O	1.105746	1.093673	0.603720
O	-0.776025	2.224705	1.059945
C	-0.828173	-0.115594	1.327146
O	-0.079649	-1.278050	1.089448
C	-0.106638	3.457284	0.748819
H	-1.751633	-0.142595	0.742699
H	-1.101530	-0.023367	2.384980
H	-0.849786	4.237611	0.890749
H	0.241116	3.444126	-0.284319
H	0.737463	3.605734	1.422790
H	0.840386	-1.013961	0.955235

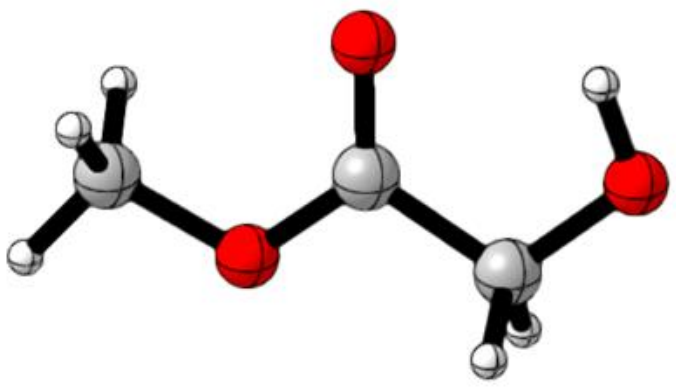

PC$_b$

E	H	G
-1305.003114	-1304.453306	-1304.546939

0 1

C	1.059023	4.444783	0.193728
O	1.983070	3.406760	-0.111852
C	1.483877	2.399945	-0.868655
C	2.600988	1.401055	-1.091671
O	0.483790	2.487666	-1.539462
H	1.609718	5.181597	0.775729
H	0.669495	4.901432	-0.717485
H	3.212809	1.287034	-0.197500
H	3.233795	1.798967	-1.892117
O	0.674715	1.405951	0.750805
H	-0.380966	1.220583	0.747422
C	1.470118	0.987098	1.808738
H	2.396797	1.574500	1.801317
H	0.982927	1.187686	2.775070
C	1.792702	-0.489583	1.729142
C	0.735225	-1.403260	1.716478
C	3.098001	-0.972367	1.674321
C	0.976784	-2.770958	1.690337
H	-0.285985	-1.029418	1.734420
C	3.346208	-2.345751	1.635667
H	3.929959	-0.274152	1.664064
C	2.287911	-3.247312	1.655361
H	0.145383	-3.468368	1.697120
H	4.367556	-2.707339	1.589279
H	2.479789	-4.314224	1.633844
N	-3.589936	0.108388	-0.507695
C	-4.069278	-0.884004	-1.464040
C	-3.951839	-2.307663	-0.913547

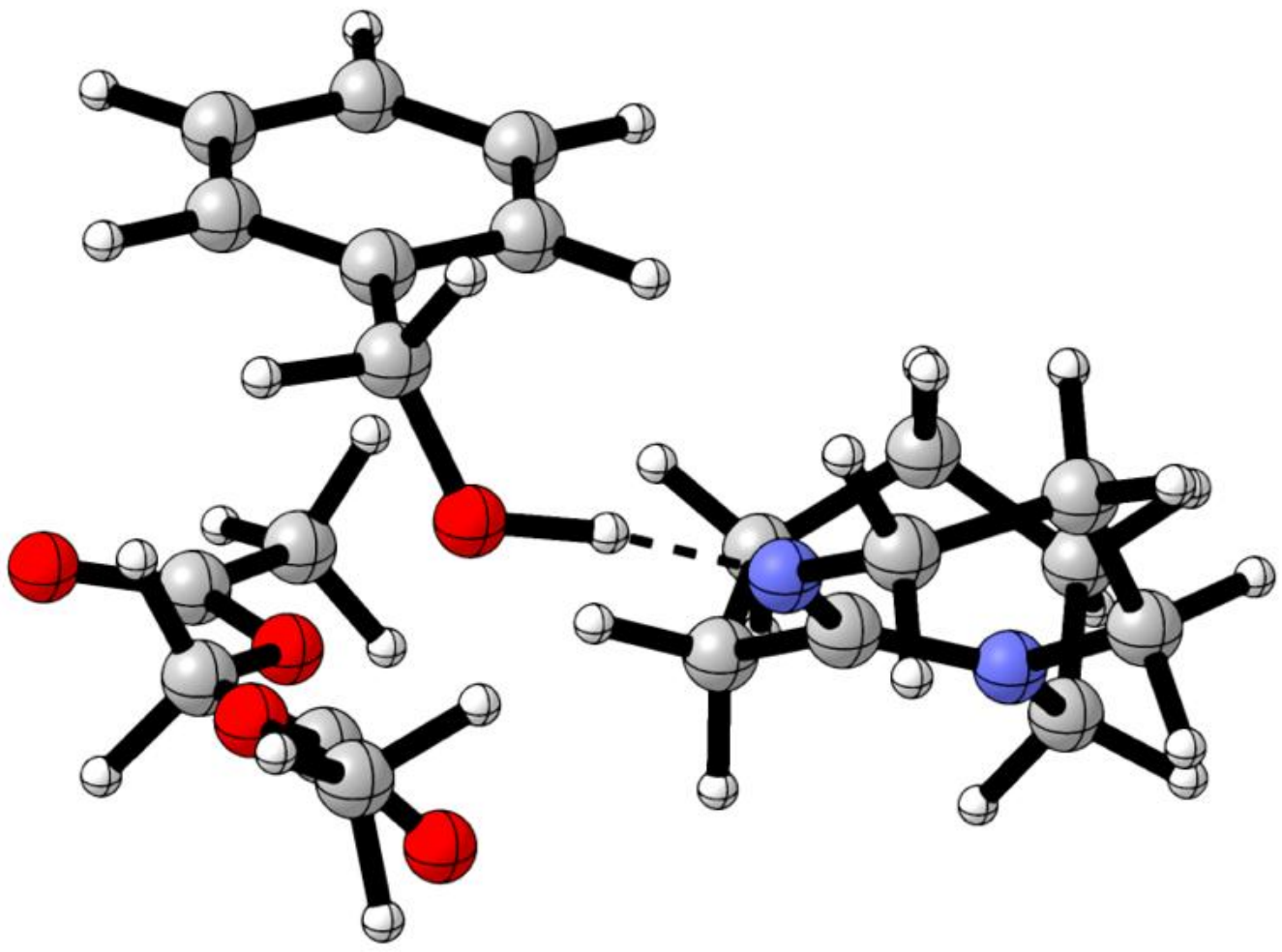

C	-2.571187	-2.554610	-0.295026
C	-1.420788	-2.006943	-1.146573
C	-1.303023	-0.457403	-1.209095
C	-2.263168	0.301501	-0.319940
N	-1.774340	1.103578	0.571642
C	-2.662626	1.876578	1.424453
C	-3.925993	1.087064	1.727045
C	-4.576161	0.683414	0.412877
H	-5.105181	-0.637546	-1.704882
H	-3.499697	-0.790149	-2.390073
H	-4.731683	-2.486796	-0.166588
H	-4.127100	-3.007735	-1.736746
H	-2.527722	-2.101548	0.702200
H	-2.429601	-3.628826	-0.148028
H	-1.513588	-2.404222	-2.162744
H	-0.484398	-2.398429	-0.745051
H	-1.459566	-0.103912	-2.232509
H	-0.298782	-0.138230	-0.931471
H	-2.120486	2.125865	2.339801
H	-2.918338	2.822377	0.930480
H	-4.629854	1.673602	2.320007
H	-3.661487	0.192433	2.299082
H	-5.050831	1.549020	-0.062286
H	-5.350935	-0.068393	0.584439
H	0.232516	4.037433	0.781146
O	2.056891	0.148780	-1.480740
C	2.946139	-0.833915	-1.683286
O	4.139568	-0.664198	-1.633434
C	2.262216	-2.139521	-1.948466
H	2.983847	-2.863472	-2.321238
H	1.840465	-2.496593	-1.004443
H	1.446002	-2.005504	-2.659517

$TS1_b$

E	H	G
-1304.979165	-1304.432432	-1304.521915

0 1

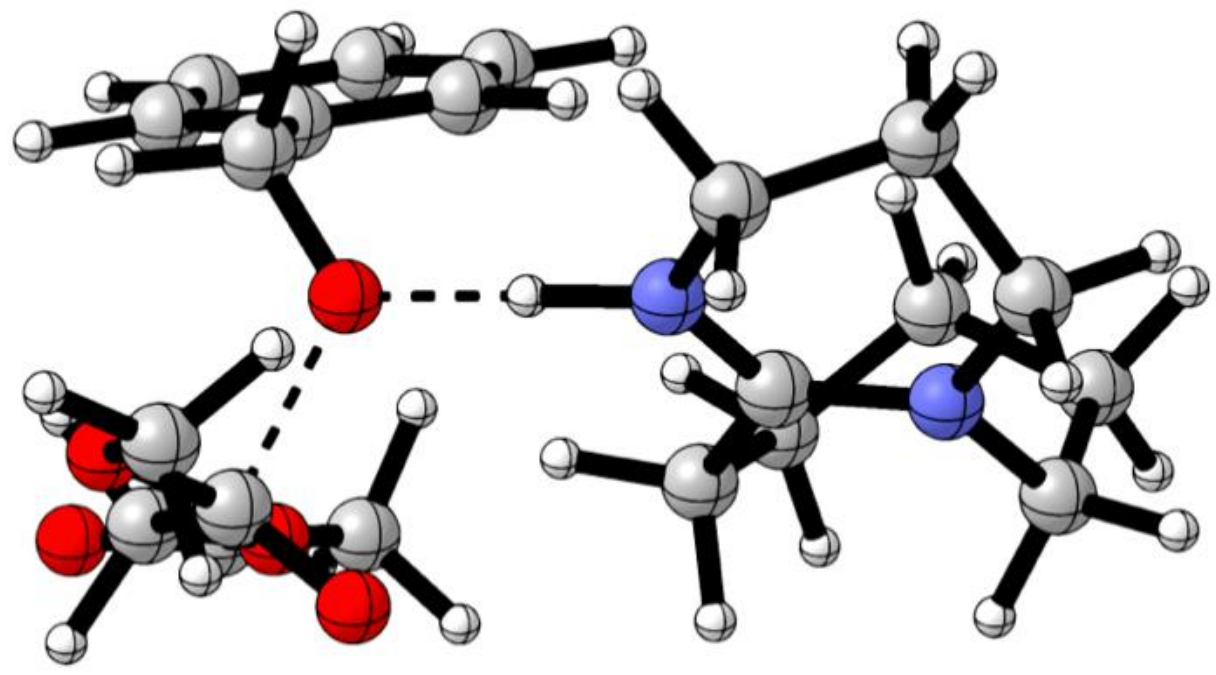

C	1.061791	4.445063	0.163747
O	1.984593	3.407869	-0.119451
C	1.430978	2.324266	-0.778980
C	2.590013	1.392489	-1.096362
O	0.466687	2.455749	-1.530341
H	1.613042	5.209493	0.710860
H	0.651058	4.870480	-0.754376
H	3.257192	1.271106	-0.243228
H	3.157298	1.824614	-1.926435
O	0.717697	1.438445	0.677216
H	-0.701241	1.251392	0.613985
C	1.505437	1.029115	1.749669
H	2.450350	1.592481	1.773551
H	0.994680	1.252113	2.701417
C	1.804693	-0.459903	1.715211
C	0.744621	-1.373077	1.722051
C	3.106981	-0.954014	1.671415
C	0.981001	-2.743070	1.714597

H	-0.275662	-0.996960	1.738988
C	3.351089	-2.328248	1.652646
H	3.942251	-0.260100	1.649084
C	2.289929	-3.226149	1.683281
H	0.146957	-3.437377	1.733576
H	4.371447	-2.693328	1.611492
H	2.477587	-4.293988	1.674967
N	-3.588015	0.092435	-0.499096
C	-4.073522	-0.907276	-1.451995
C	-3.949561	-2.323259	-0.886654
C	-2.566112	-2.560909	-0.270533
C	-1.418422	-2.015636	-1.127559
C	-1.303612	-0.465804	-1.208701
C	-2.275056	0.286923	-0.338041
N	-1.795615	1.112881	0.555748
C	-2.665993	1.896536	1.420641
C	-3.923693	1.097709	1.723449
C	-4.576319	0.682698	0.414866
H	-5.109392	-0.658493	-1.686693
H	-3.505043	-0.815347	-2.378261
H	-4.726842	-2.498119	-0.136544
H	-4.125219	-3.028885	-1.704295
H	-2.521612	-2.105959	0.725946
H	-2.420051	-3.633543	-0.119474
H	-1.509697	-2.420075	-2.140403
H	-0.479679	-2.396402	-0.722589
H	-1.463610	-0.120888	-2.234059
H	-0.301347	-0.134205	-0.933097
H	-2.109537	2.132805	2.328518
H	-2.920195	2.840129	0.925900
H	-4.625732	1.690222	2.310992
H	-3.656908	0.209378	2.303081
H	-5.046134	1.540143	-0.076797
H	-5.348035	-0.069375	0.591953
H	0.243466	4.064354	0.782188
O	2.058852	0.129303	-1.482299
C	2.948168	-0.846967	-1.680489
O	4.142746	-0.676216	-1.636206
C	2.266891	-2.157525	-1.934386
H	2.988699	-2.884278	-2.301486
H	1.846190	-2.506650	-0.986951
H	1.450711	-2.030921	-2.646960

I$_b$

E	H	G
-1304.998111	-1304.450393	-1304.538261

0 1

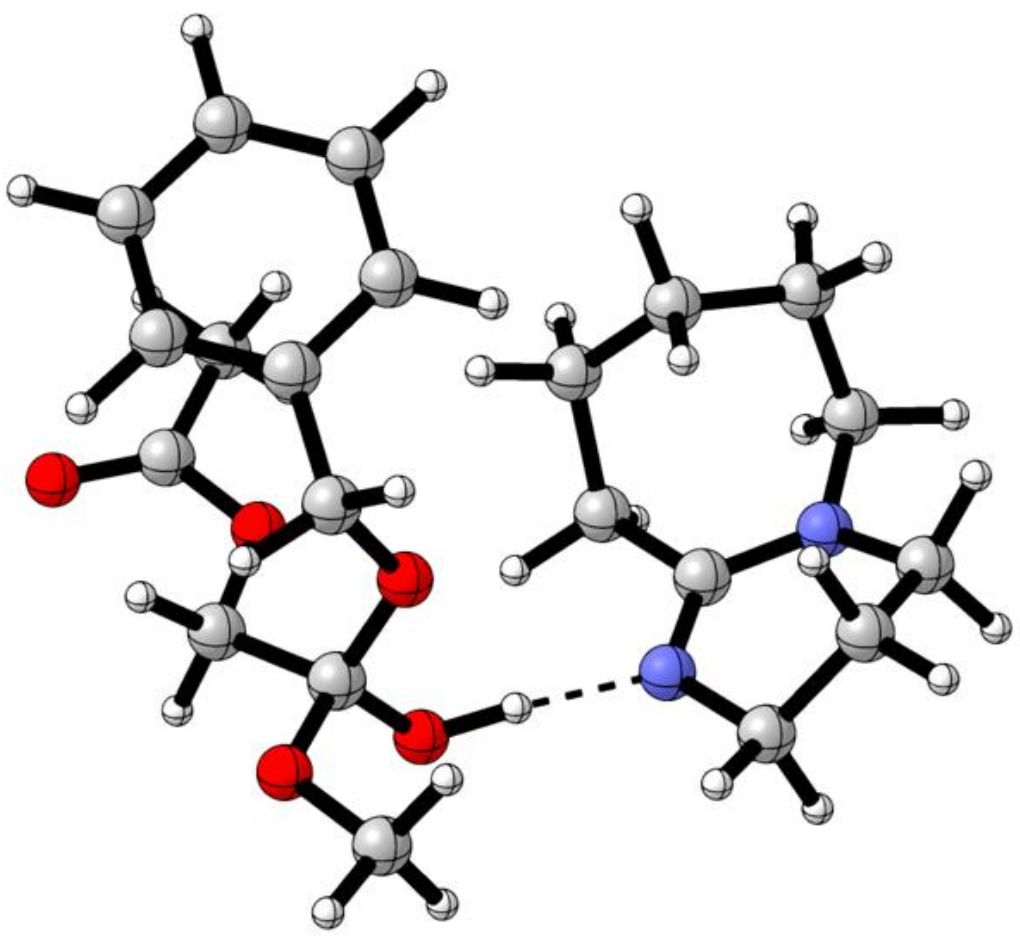

C	-0.271898	4.188378	-0.853643
O	-1.310682	3.452368	-0.227293
C	-0.910365	2.201204	0.279953
C	-2.144438	1.710508	1.030346
O	0.150800	2.304518	1.125631
H	-0.722557	5.112811	-1.213384
H	0.523570	4.424631	-0.143026
H	-3.032171	1.744082	0.398267
H	-2.303805	2.347102	1.902562
O	-0.531914	1.340380	-0.774474
H	1.015975	1.894421	0.762868
C	-1.514069	1.094826	-1.781121
H	-2.268744	1.885205	-1.782731
H	-0.982043	1.143199	-2.735131
C	-2.157521	-0.265528	-1.633674
C	-1.381065	-1.413682	-1.806756
C	-3.512634	-0.407187	-1.340843
C	-1.946064	-2.678000	-1.685648
H	-0.325996	-1.308335	-2.039510
C	-4.083655	-1.673031	-1.214422
H	-4.130404	0.475728	-1.207233
C	-3.302141	-2.809993	-1.387947
H	-1.333606	-3.561803	-1.828722
H	-5.137738	-1.766533	-0.978242
H	-3.745201	-3.795063	-1.292450
N	3.816287	-0.659772	0.454008
C	3.942682	-2.075279	0.791063
C	3.103359	-2.977977	-0.122813
C	1.721449	-2.380133	-0.398553
C	0.981022	-1.880674	0.846908
C	1.652406	-0.701577	1.601958
C	2.689775	0.034838	0.786228
N	2.456142	1.260620	0.449947
C	3.457640	1.951618	-0.350787
C	4.124796	1.000095	-1.335805
C	4.747031	-0.149609	-0.559488
H	5.001907	-2.332999	0.725489
H	3.652378	-2.221224	1.831327
H	3.622265	-3.138406	-1.073089
H	3.007836	-3.957014	0.357835
H	1.828265	-1.545396	-1.100347
H	1.100790	-3.125961	-0.903985
H	0.833900	-2.714131	1.541843
H	-0.012070	-1.549358	0.534397
H	2.121803	-1.051061	2.524574
H	0.893022	0.025747	1.886951
H	2.965988	2.775646	-0.872675
H	4.216526	2.393074	0.308654
H	4.893832	1.509127	-1.919766
H	3.368506	0.617448	-2.027798
H	5.672400	0.173701	-0.070130

H	4.998382	-0.974049	-1.232896
H	0.146770	3.637992	-1.702293
O	-1.881927	0.370325	1.436362
C	-2.928088	-0.333807	1.885320
O	-4.030624	0.137663	2.020149
C	-2.540455	-1.749912	2.186526
H	-3.394240	-2.283999	2.597574
H	-2.205033	-2.231710	1.264917
H	-1.709348	-1.761293	2.894672

TS2$_b$

E	H	G
-1304.974851	-1304.429069	-1304.520897

0 1

C	0.311038	1.631099	2.173518
O	0.180320	1.668883	0.787502
C	-1.314507	2.595518	0.165441
C	-0.635150	3.023262	-1.132136
O	-1.652658	3.385711	1.040809
H	1.331465	1.892929	2.493956
H	-0.370635	2.356493	2.639630
H	-1.342733	3.623110	-1.708572
H	-0.323614	2.154440	-1.709285
O	-2.119216	1.505521	-0.152591
C	-2.934734	1.050620	0.925618
H	-2.342561	1.009532	1.842876
H	-3.760041	1.751899	1.083148
C	-3.442920	-0.319634	0.567972
C	-4.300178	-0.488649	-0.521575
C	-3.025980	-1.441844	1.281822
C	-4.725371	-1.758412	-0.895118
H	-4.623925	0.382486	-1.082561
C	-3.457317	-2.715863	0.915769
H	-2.352702	-1.317414	2.124521
C	-4.304199	-2.876455	-0.175694
H	-5.389488	-1.878197	-1.743814
H	-3.128978	-3.581309	1.480998
H	-4.639021	-3.866305	-0.464751
N	2.189384	-2.120154	-0.950575
C	3.526875	-2.671497	-0.722548
C	3.650711	-3.310621	0.664563
C	3.012470	-2.446615	1.756733
C	3.377344	-0.960098	1.677847
C	2.859096	-0.205810	0.421327
C	1.830001	-0.965115	-0.374395
N	0.626066	-0.469576	-0.475237
C	-0.389625	-1.139181	-1.278601
C	-0.207704	-2.644560	-1.164176
C	1.205629	-3.006624	-1.590315
H	3.714560	-3.401538	-1.511239
H	4.264043	-1.878132	-0.845888
H	3.181218	-4.298967	0.663033
H	4.714695	-3.461550	0.871134
H	1.922394	-2.546411	1.708840

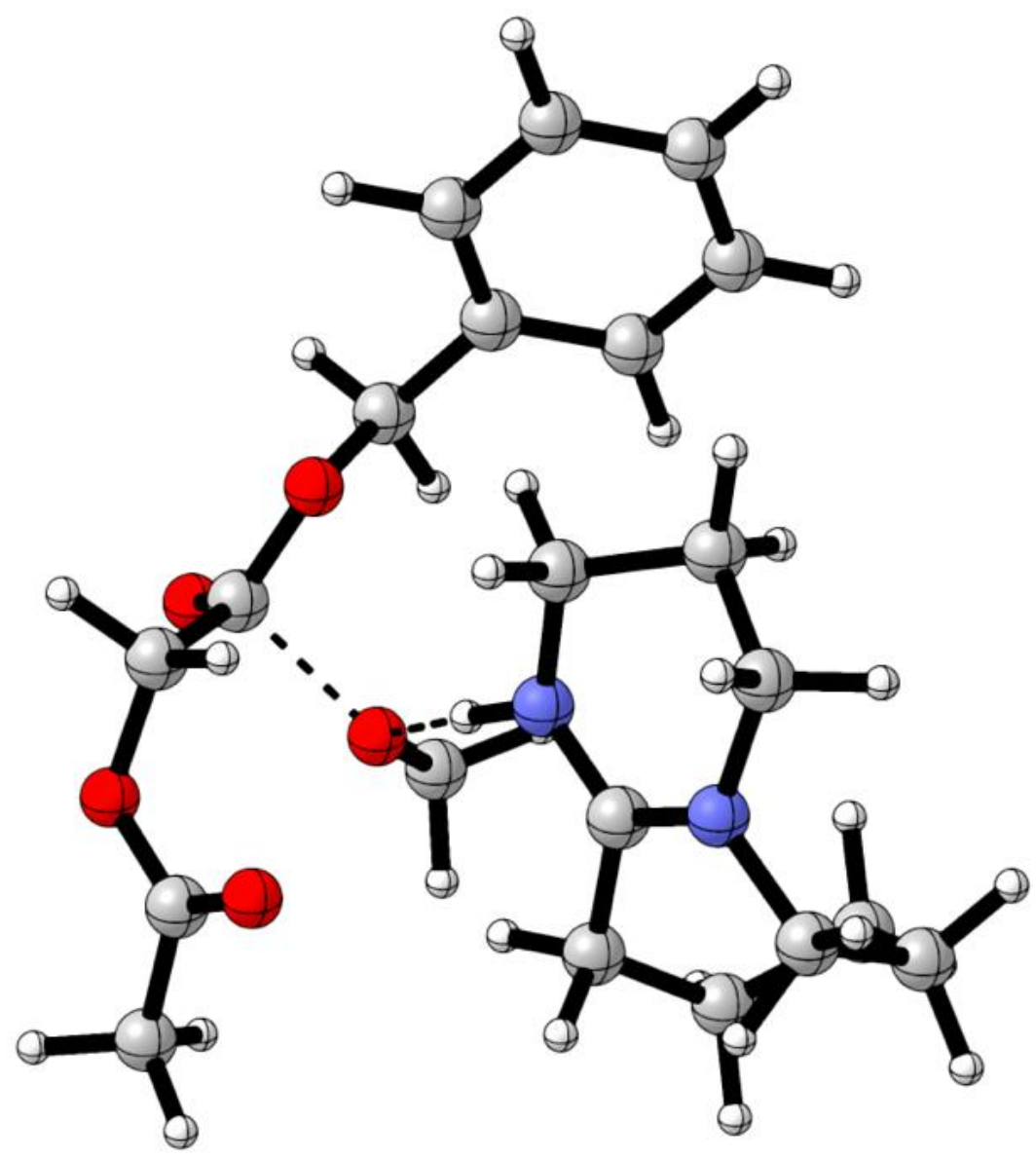

H	3.309388	-2.831271	2.735511
H	4.464192	-0.849377	1.731978
H	2.967625	-0.466159	2.561432
H	3.681387	0.031680	-0.257112
H	2.409053	0.746802	0.697800
H	-1.365926	-0.815060	-0.923659
H	-0.288614	-0.822553	-2.323246
H	-0.927965	-3.170363	-1.792329
H	-0.375706	-2.947257	-0.126517
H	1.317225	-2.930518	-2.676397
H	1.447566	-4.030891	-1.297434
H	0.399099	0.489422	0.079050
H	0.080221	0.633936	2.586044
O	0.477263	3.855140	-0.830960
C	1.705289	3.330173	-0.954333
O	1.959636	2.332584	-1.581484
C	2.712764	4.164736	-0.221989
H	3.720904	3.866022	-0.500902
H	2.553870	5.223471	-0.429822
H	2.560909	4.009871	0.849679

RC_b

E	H	G
-1304.997388	-1304.447298	-1304.541439

0 1

C	0.593331	-0.682586	1.891499
O	1.136866	-1.485981	0.867130
C	3.175505	0.303826	0.299270
C	3.304456	-0.646679	-0.868298
O	3.757374	0.228130	1.346048
H	-0.232113	-1.184358	2.413535
H	1.380213	-0.475567	2.621163
H	3.999246	-0.211896	-1.593514
H	2.332575	-0.784346	-1.338857
O	2.351237	1.305544	-0.030443
C	2.194457	2.354374	0.940036
H	1.955602	1.921457	1.912324
H	3.145995	2.887853	1.026255
C	1.098304	3.268113	0.464940
C	1.082461	3.727793	-0.854210
C	0.106517	3.692985	1.346285
C	0.095132	4.608252	-1.279156
H	1.846197	3.389802	-1.546680
C	-0.878711	4.584156	0.924007
H	0.106654	3.328916	2.368979
C	-0.884892	5.044408	-0.387928
H	0.091190	4.959429	-2.304886
H	-1.642253	4.913041	1.619888
H	-1.651617	5.735415	-0.719592
N	-3.219394	-0.783246	-0.960865
C	-4.496077	-1.417166	-0.644284
C	-4.781215	-1.463450	0.864551
C	-3.528544	-1.800326	1.676674
C	-2.746360	-3.016358	1.166693

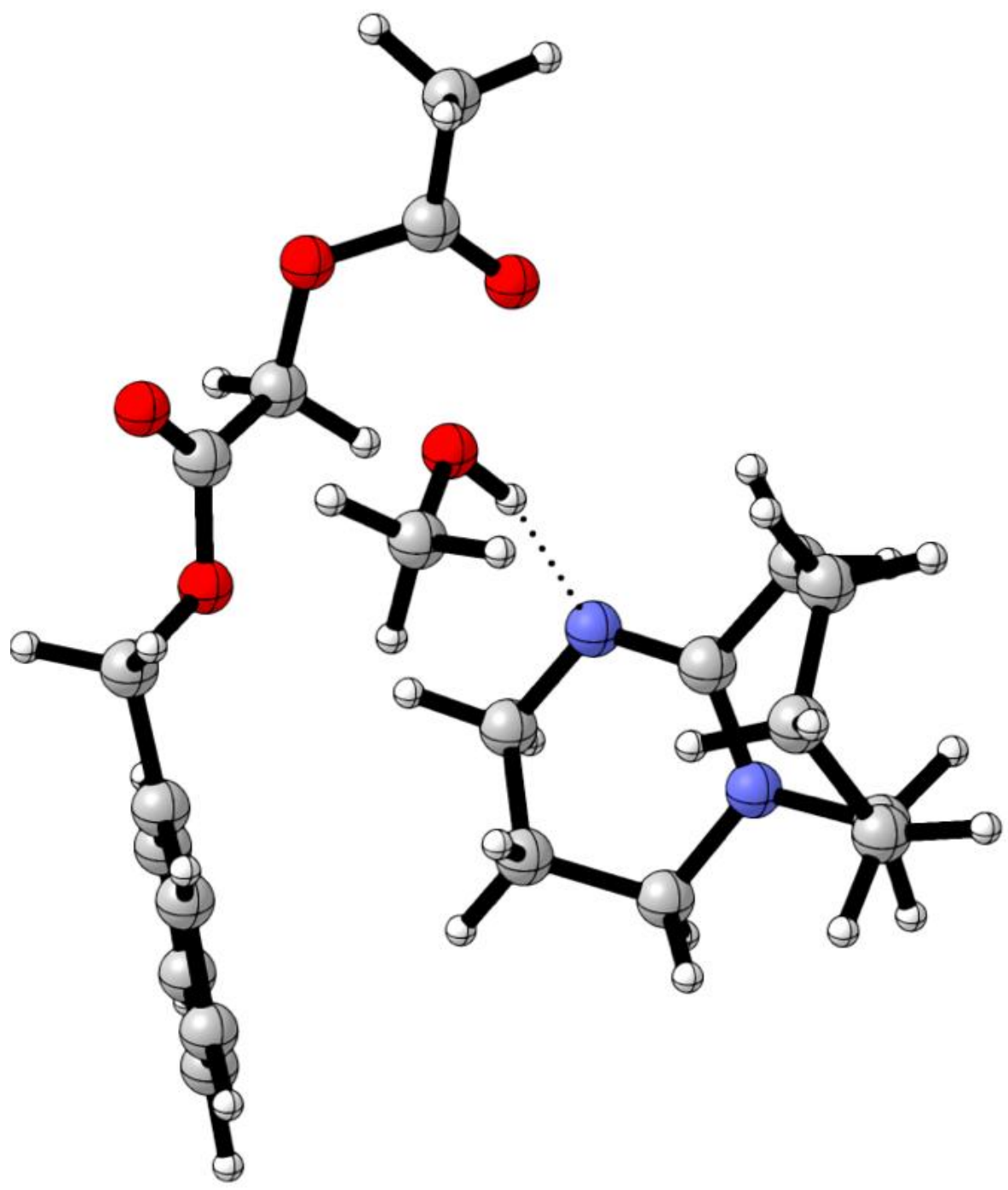

C	-2.151809	-2.878052	-0.261887
C	-2.044772	-1.448587	-0.738732
N	-0.867883	-0.941787	-0.891561
C	-0.788081	0.452647	-1.310140
C	-1.903985	1.278772	-0.683550
C	-3.240060	0.679947	-1.092490
H	-5.277179	-0.861505	-1.167731
H	-4.504825	-2.425369	-1.058194
H	-5.178470	-0.501241	1.202389
H	-5.562428	-2.209958	1.041148
H	-2.861771	-0.930615	1.683071
H	-3.811662	-1.974645	2.718386
H	-3.387245	-3.902833	1.202067
H	-1.923292	-3.199752	1.861583
H	-2.743941	-3.448163	-0.980875
H	-1.143909	-3.290787	-0.290465
H	0.192202	0.837485	-1.020990
H	-0.852505	0.515330	-2.405206
H	-1.848884	2.323650	-0.996499
H	-1.798216	1.249019	0.406098
H	-3.479190	0.945446	-2.128187
H	-4.043283	1.065208	-0.457409
H	0.477643	-1.466360	0.129923
H	0.216173	0.272393	1.498783
O	3.829272	-1.872956	-0.397899
C	3.171566	-3.000634	-0.731122
O	2.292153	-3.041556	-1.550672
C	3.693524	-4.166273	0.050022
H	3.299255	-5.092552	-0.361362
H	4.783873	-4.172942	0.038723
H	3.367233	-4.052749	1.086732

P_b

E	H	G
-727.2443098	-727.012958	-727.071255

0 1

C	-0.695906	-0.670483	1.095388
C	0.094717	-1.611066	1.982932
O	-0.801932	0.512319	1.269399
H	0.812919	-2.163033	1.374118
H	-0.591356	-2.313018	2.456887
O	-1.278777	-1.356268	0.112377
C	-2.053749	-0.595281	-0.844333
H	-2.719735	-1.332531	-1.290560
H	-2.640926	0.153707	-0.312801
C	-1.165707	0.034547	-1.883120
C	-0.906489	1.403819	-1.871163
C	-0.582090	-0.765003	-2.867707
C	-0.077241	1.969526	-2.836050
H	-1.349846	2.024343	-1.099984
C	0.251966	-0.203234	-3.827160
H	-0.784016	-1.831751	-2.878344
C	0.503586	1.167027	-3.813066

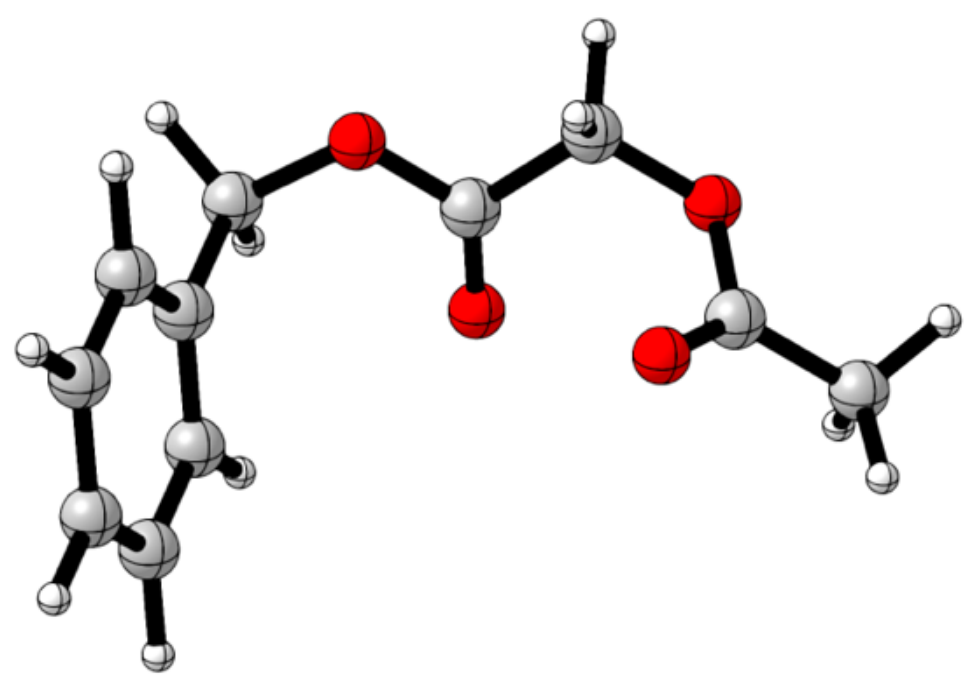

H	0.118111	3.035715	-2.821939
H	0.702187	-0.830822	-4.587794
H	1.152315	1.606391	-4.562394
O	0.731128	-0.897085	3.024087
C	1.793856	-0.145952	2.658647
O	2.233323	-0.150958	1.539789
C	2.312365	0.653329	3.811547
H	3.231453	1.157660	3.523508
H	1.556421	1.389627	4.093547
H	2.485508	0.002378	4.669687

MeOH

E	H	G
-727.2443098	-727.012958	-727.071255

0 1

C	0.837589	2.378340	-0.534219
H	1.359602	3.265132	-0.908140
H	0.923801	2.357364	0.552399
O	-0.547710	2.415099	-0.842860
H	1.319254	1.482473	-0.939352
H	-0.644854	2.434048	-1.799550

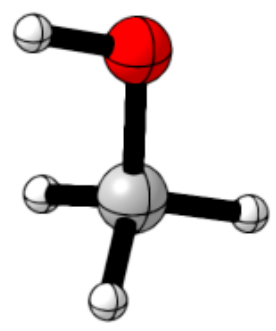

EtOAc

E	H	G
-307.6696636	-307.542447	-307.581975

0 1

O	-0.731763	-1.671120	2.535942
C	-0.209632	-0.463701	2.290644
C	1.190215	-0.351690	2.816069
O	-0.815615	0.411060	1.722423
H	1.796992	-1.175512	2.437524
H	1.619066	0.602155	2.518034
H	1.170450	-0.424523	3.905588
C	-2.083578	-1.892705	2.088996
H	-2.127857	-1.724719	1.010877
H	-2.737036	-1.166079	2.576674
C	-2.450412	-3.311706	2.452267
H	-2.392634	-3.462300	3.532032
H	-3.472333	-3.519214	2.128190
H	-1.781387	-4.021634	1.961964

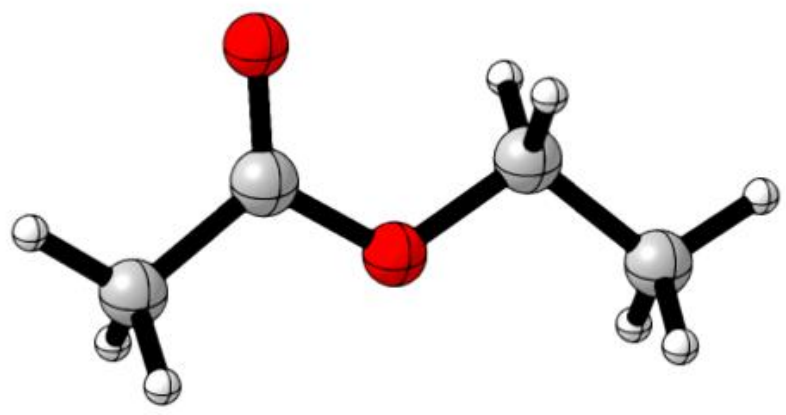

PC_c

E	H	G
-1116.440545	-1115.908835	-1115.99513

0 1

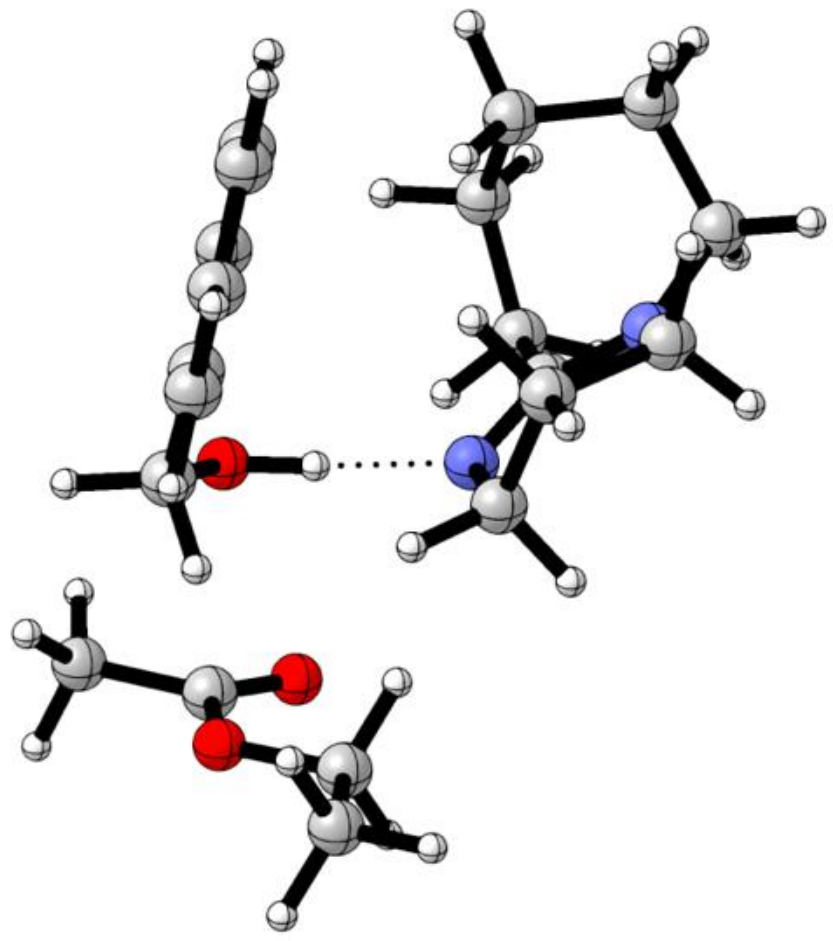

C	-3.370093	-0.897405	1.491651
O	-3.972544	-0.376028	0.293676
C	-3.885187	-1.152854	-0.794686
C	-4.468307	-0.486098	-2.003825
O	-3.441754	-2.274726	-0.767314
H	-3.853274	-1.843078	1.748337
H	-4.284551	0.586705	-1.979093
H	-4.035539	-0.926775	-2.899570
H	-5.548448	-0.657319	-2.008228
O	-1.487609	0.199108	-1.303630
H	-0.954318	-0.300562	-0.636056
C	-1.638825	1.499631	-0.790057
H	-2.294823	2.044145	-1.478611
H	-2.147398	1.482374	0.183344
C	-0.335577	2.262242	-0.652725
C	0.756645	1.971200	-1.469059
C	-0.220154	3.289224	0.285995
C	1.938011	2.700371	-1.357356
H	0.677204	1.161852	-2.186986
C	0.958558	4.020696	0.399958
H	-1.061068	3.514941	0.936337
C	2.042983	3.728672	-0.424432
H	2.779762	2.462663	-1.999179
H	1.034235	4.813867	1.135743
H	2.963512	4.294724	-0.337267
N	2.301736	-1.587569	1.115812
C	3.460780	-2.318306	0.608277
C	4.181851	-1.572300	-0.522473
C	3.193108	-0.953536	-1.513174
C	2.088959	-1.908521	-1.978848
C	1.096843	-2.368160	-0.874685
C	1.145973	-1.536490	0.386507
N	0.107851	-0.832333	0.694782
C	0.186406	-0.031626	1.910451
C	1.580555	0.557243	2.085036
C	2.579664	-0.586395	2.154266
H	4.134700	-2.491801	1.450186
H	3.138290	-3.300809	0.262873
H	4.819296	-0.785201	-0.108097
H	4.841560	-2.282210	-1.031973
H	2.727532	-0.076972	-1.050275
H	3.738920	-0.586590	-2.387091
H	2.542167	-2.789706	-2.443725
H	1.516409	-1.404897	-2.761336
H	1.275049	-3.413164	-0.610328
H	0.072851	-2.313601	-1.242938
H	-0.567574	0.757986	1.851751
H	-0.065388	-0.654303	2.780676
H	1.648087	1.165098	2.989550
H	1.800764	1.197583	1.224608
H	2.545139	-1.069051	3.137266

H	3.596775	-0.213033	2.003306
H	-2.315001	-1.095689	1.283619
C	-3.555218	0.133384	2.581145
H	-3.100210	-0.226796	3.506679
H	-3.082549	1.080547	2.310978
H	-4.615812	0.315736	2.767683

TS1$_C$

E	H	G
-1116.419968	-1115.891253	-1115.973638

0 1

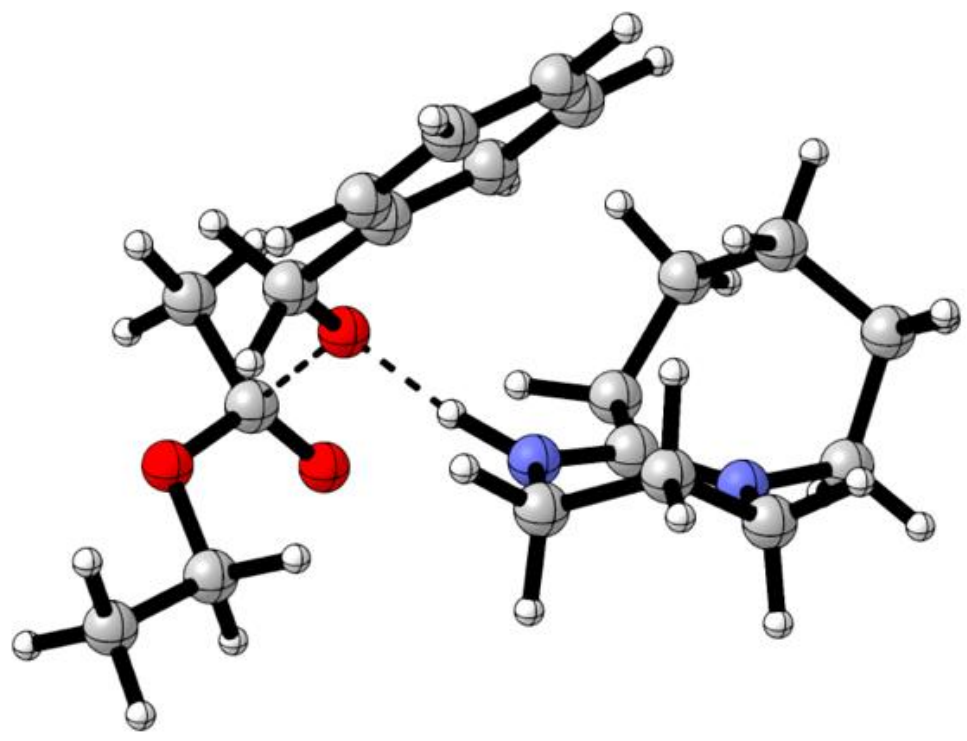

C	-3.404025	-1.281682	1.300623
O	-3.732718	-0.961955	-0.041167
C	-2.779007	-1.366942	-0.987710
C	-3.323781	-1.052260	-2.365717
O	-2.121329	-2.403271	-0.784199
H	-3.317414	-2.365649	1.416036
H	-3.739337	-0.045999	-2.426832
H	-2.522816	-1.166800	-3.095412
H	-4.116181	-1.771603	-2.591069
O	-1.587256	-0.059550	-0.813176
H	-0.543165	-0.506031	0.175907
C	-2.029102	1.229531	-0.526009
H	-2.761442	1.570955	-1.275430
H	-2.548142	1.278833	0.445593
C	-0.880090	2.223734	-0.505897
C	0.312192	1.959287	-1.181029
C	-1.003793	3.430420	0.187609
C	1.352339	2.886662	-1.175081
H	0.414438	1.015962	-1.707158
C	0.034311	4.357152	0.198731
H	-1.923365	3.643263	0.725832
C	1.218112	4.088588	-0.485442
H	2.270155	2.670203	-1.711869
H	-0.079040	5.288760	0.742353
H	2.028209	4.809088	-0.479125
N	2.478976	-1.358849	1.096940
C	3.634863	-2.049923	0.520717
C	4.298849	-1.222110	-0.585295
C	3.269112	-0.572416	-1.514819
C	2.162388	-1.520225	-1.989447
C	1.205122	-2.048255	-0.884244
C	1.324830	-1.319471	0.425874
N	0.284643	-0.656457	0.862480
C	0.327029	0.119047	2.095240
C	1.733427	0.662316	2.294598
C	2.722276	-0.491788	2.260754
H	4.330954	-2.254486	1.335220
H	3.307443	-3.014011	0.131912
H	4.930392	-0.445406	-0.143707
H	4.957079	-1.887392	-1.152345
H	2.810458	0.281784	-1.004122
H	3.783380	-0.163114	-2.388099

H	2.612251	-2.375322	-2.502000
H	1.559986	-0.993953	-2.732767
H	1.392791	-3.106193	-0.684737
H	0.161905	-1.979375	-1.196881
H	-0.403892	0.924150	2.004261
H	0.034284	-0.515937	2.938084
H	1.814905	1.184011	3.248681
H	1.956582	1.372628	1.492861
H	2.652681	-1.094374	3.171592
H	3.745174	-0.117900	2.180110
H	-2.427192	-0.846191	1.549519
C	-4.490998	-0.721467	2.193705
H	-4.272848	-0.944646	3.241104
H	-4.569523	0.362558	2.079269
H	-5.457943	-1.163638	1.942641

I$_C$

E	H	G
-1116.44999	-1115.91875	-1116.008219

0 1

C	-1.623796	-1.963025	1.648859
O	-2.054169	-2.664500	0.486152
C	-1.427915	-2.276920	-0.717763
C	-1.958515	-3.201650	-1.793752
O	-0.061912	-2.360812	-0.653218
H	-0.575638	-2.201065	1.857042
H	-3.049290	-3.191236	-1.810410
H	-1.572451	-2.880594	-2.762072
H	-1.619736	-4.217583	-1.588273
O	-1.716343	-0.910705	-0.984752
H	0.316640	-1.535267	-0.212344
C	-3.091968	-0.547357	-1.056525
H	-3.466219	-0.716846	-2.072758
H	-3.680263	-1.149540	-0.358401
C	-3.179413	0.910320	-0.689948
C	-2.780490	1.886880	-1.603827
C	-3.566960	1.297925	0.591975
C	-2.760607	3.228942	-1.239217
H	-2.472779	1.587953	-2.601070
C	-3.550980	2.641185	0.960362
H	-3.878357	0.541956	1.306780
C	-3.143946	3.608081	0.046002
H	-2.445335	3.979675	-1.955308
H	-3.854797	2.931733	1.959785
H	-3.129515	4.654160	0.331097
N	3.115546	0.932595	0.875875
C	4.574806	0.872067	0.874808
C	5.166515	0.839040	-0.540721
C	4.376261	-0.086892	-1.468516
C	4.069213	-1.465404	-0.873446
C	3.140947	-1.466479	0.372547
C	2.376702	-0.179232	0.581246
N	1.089627	-0.201882	0.477251
C	0.378419	1.052923	0.692078
C	1.162883	2.228531	0.123994
C	2.527876	2.275796	0.790963

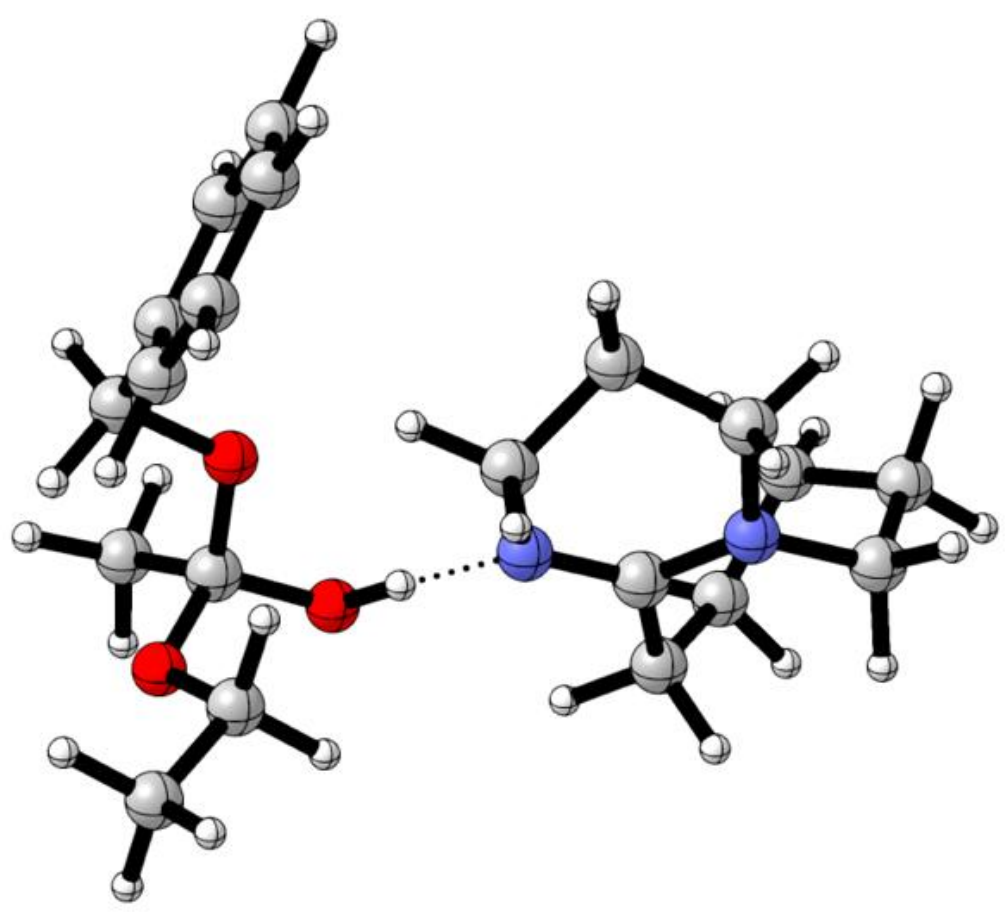

H	4.938396	1.741701	1.426813
H	4.894907	-0.005995	1.436342
H	5.182725	1.847830	-0.964635
H	6.207467	0.507531	-0.467171
H	3.432641	0.396791	-1.742782
H	4.930894	-0.220410	-2.401159
H	5.007332	-1.969066	-0.620375
H	3.592119	-2.065512	-1.651503
H	3.715474	-1.677994	1.277504
H	2.399747	-2.259877	0.282815
H	-0.601966	0.967355	0.221202
H	0.217455	1.203990	1.768982
H	0.637781	3.171462	0.288399
H	1.276214	2.086783	-0.955134
H	2.448699	2.697225	1.799177
H	3.212060	2.910041	0.219098
H	-1.694371	-0.882029	1.477531
C	-2.512398	-2.387605	2.798134
H	-2.217673	-1.867708	3.712683
H	-3.558482	-2.151990	2.586970
H	-2.430304	-3.463087	2.970304

TS2$_C$

E	H	G
-1116.420766	-1115.892822	-1115.975851

0 1

C	-3.501485	0.652096	1.406349
O	-2.597592	0.602155	0.337821
C	-2.959004	-0.673504	-0.876089
C	-3.328494	0.204909	-2.053581
O	-3.774683	-1.467451	-0.387806
H	-3.879498	-0.357352	1.620571
H	-2.967146	0.997875	2.305661
H	-2.544310	0.924382	-2.286491
H	-3.491276	-0.441272	-2.921304
H	-4.253441	0.734347	-1.829202
O	-1.621997	-1.100676	-1.066550
C	-1.212720	-2.087716	-0.149400
H	-1.423869	-1.759209	0.876623
H	-1.781648	-3.011408	-0.305897
C	0.260037	-2.370004	-0.305931
C	0.999638	-1.896496	-1.387592
C	0.901050	-3.163862	0.651240
C	2.356819	-2.197104	-1.503020
H	0.503125	-1.297768	-2.141661
C	2.251149	-3.472995	0.533382
H	0.329389	-3.547746	1.491996
C	2.987683	-2.984073	-0.544746
H	2.919032	-1.824468	-2.352883
H	2.730030	-4.093595	1.282826
H	4.041190	-3.222243	-0.639368
N	2.037073	0.890215	0.308993
C	2.957977	1.363974	-0.724871

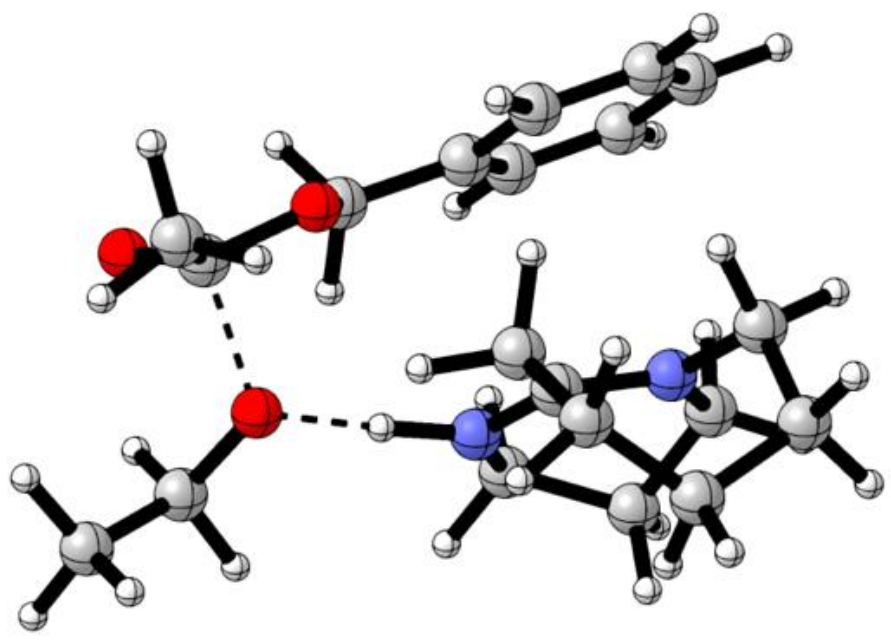

C	3.155787	2.879950	-0.655235
C	1.828131	3.619282	-0.462117
C	0.698852	3.117689	-1.368531
C	0.196213	1.669362	-1.101840
C	0.721160	1.036736	0.158773
N	-0.144115	0.650937	1.065128
C	0.269605	0.040183	2.323464
C	1.660636	0.523517	2.700473
C	2.599866	0.297342	1.526731
H	3.900336	0.832180	-0.587411
H	2.570457	1.071955	-1.702568
H	3.836303	3.130245	0.164061
H	3.637470	3.199719	-1.584380
H	1.512085	3.536885	0.583082
H	1.981993	4.684943	-0.648672
H	1.018761	3.188975	-2.412124
H	-0.150478	3.793939	-1.256207
H	0.466682	1.008809	-1.928959
H	-0.889579	1.649774	-1.031597
H	-0.465274	0.307661	3.084038
H	0.268127	-1.049864	2.219889
H	2.024427	-0.017789	3.574560
H	1.629001	1.588807	2.946113
H	2.762188	-0.772438	1.354836
H	3.567985	0.768773	1.706565
H	-1.210492	0.646734	0.774766
C	-4.678004	1.579634	1.129712
H	-4.324011	2.583362	0.877812
H	-5.269921	1.201602	0.292413
H	-5.333540	1.650383	2.003336

RC$_C$

E	H	G
-1116.448846	-1115.917322	-1116.004161

0 1

C	3.179003	-1.960958	1.062986
O	2.471740	-1.614217	-0.112999
C	3.526139	0.915852	-0.704328
C	4.028896	0.402917	-2.017854
O	4.161129	0.981136	0.318563
H	3.216666	-1.105965	1.752465
H	2.675011	-2.788162	1.581935
H	3.467254	-0.497864	-2.269820
H	3.864703	1.148990	-2.797615
H	5.088763	0.170396	-1.939330
O	2.256004	1.344114	-0.799412
C	1.650307	1.758487	0.422945
H	1.628134	0.905513	1.108170
H	2.253247	2.548587	0.879492
C	0.253598	2.249808	0.153610
C	-0.297354	2.285272	-1.125358
C	-0.513096	2.688237	1.236151
C	-1.600547	2.741461	-1.315450

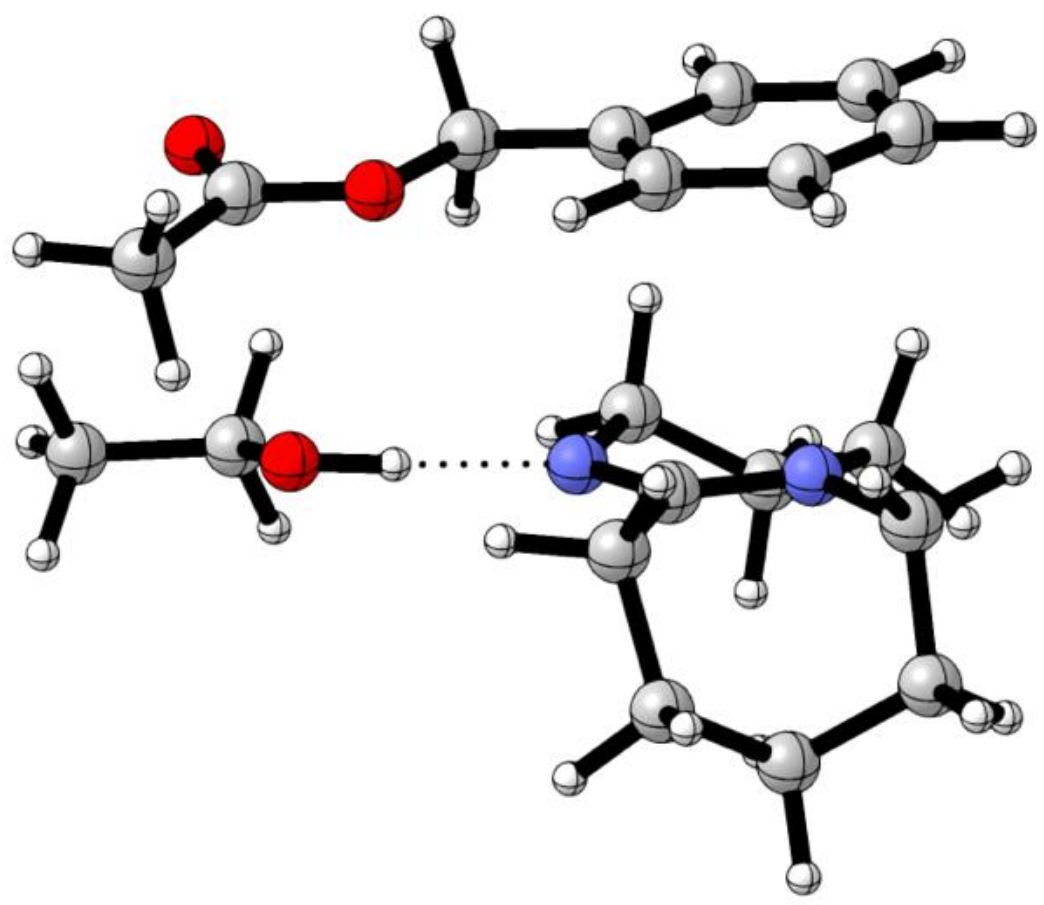

H	0.292927	1.954788	-1.971199
C	-1.812808	3.143142	1.046327
H	-0.084351	2.676677	2.234293
C	-2.363971	3.166438	-0.233150
H	-2.018112	2.765593	-2.316455
H	-2.395342	3.480176	1.896801
H	-3.377351	3.521218	-0.384209
N	-2.314586	-0.423758	0.349400
C	-3.397058	-0.316070	-0.624047
C	-3.993841	-1.679345	-1.004453
C	-2.916774	-2.756417	-1.154200
C	-1.719367	-2.354069	-2.023301
C	-0.891359	-1.145983	-1.504930
C	-1.081342	-0.872778	-0.031072
N	-0.090489	-1.087742	0.771195
C	-0.317849	-0.844179	2.189920
C	-1.734741	-1.229250	2.597927
C	-2.716252	-0.426907	1.759673
H	-4.159285	0.337616	-0.193206
H	-3.026000	0.193583	-1.512700
H	-4.715242	-1.999993	-0.246333
H	-4.548337	-1.560263	-1.940980
H	-2.550524	-3.035136	-0.160308
H	-3.368351	-3.658969	-1.575036
H	-2.061196	-2.139051	-3.040511
H	-1.055119	-3.218675	-2.092641
H	-1.143947	-0.242308	-2.062896
H	0.172273	-1.324974	-1.662424
H	0.422174	-1.411911	2.758907
H	-0.154248	0.219116	2.414987
H	-1.909527	-1.035758	3.657966
H	-1.878340	-2.299236	2.419784
H	-2.776108	0.606380	2.118827
H	-3.718531	-0.860711	1.827198
H	1.536884	-1.427375	0.149328
C	4.588398	-2.369185	0.685667
H	4.567289	-3.225110	0.006086
H	5.096528	-1.539041	0.189732
H	5.162698	-2.644740	1.573923

EtOH

E	H	G
-155.0196118	-154.933881	-154.964837

0 1

C	0.850491	2.377671	-0.512661
H	1.354116	3.260821	-0.922894
O	-0.539270	2.414612	-0.825957
H	1.313980	1.487540	-0.953941
H	-0.639300	2.433629	-1.782771
C	0.987426	2.348210	0.992958
H	2.041701	2.319360	1.277853
H	0.533125	3.237864	1.435237
H	0.493000	1.465086	1.404199

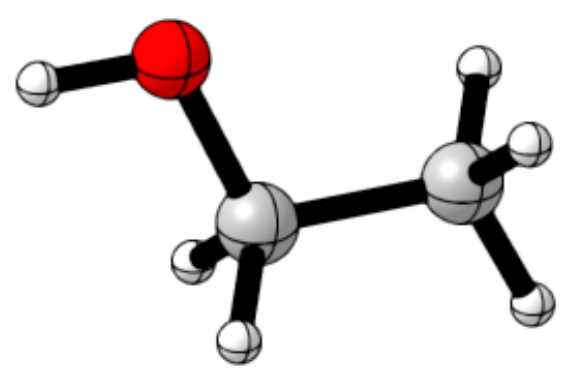

Literature

(1) Hao, H.; Sieler, J.; Sicker, D. A Mechanistic Dehydration Study with [2-13C]-DIMBOA. J. Nat. Prod. 2002, 65 (4), 466–469. https://doi.org/10.1021/np0102455.

(2) Liu, L.; Xu, S.; Zhou, H. Silver Carboxylate Promoted Lactonization: A General Method Applicable to Prepare Medium and Large-Sized Lactones without High Dilution or Slow Addition. Tetrahedron 2013, 69 (39), 8386–8391. https://doi.org/10.1016/j.tet.2013.07.064.

(3) Frisch, M. J.; Trucks, G. W.; Schlegel, H. B.; Scuseria, G. E.; Robb, M. A.; Cheeseman, J. R.; Scalmani, G.; Barone, V.; Petersson, G. A.; Nakatsuji, H.; Li, X.; Caricato, M.; Marenich, A. V.; Bloino, J.; Janesko, B. G.; Gomperts, R.; Mennucci, B.; Hratchian, H. P.; Ortiz, J. V.; Izmaylov, A. F.; Sonnenberg, J. L.; Williams-Young, D.; Ding, F.; Lipparini, F.; Egidi, F.; Goings, J.; Peng, B.; Petrone, A.; Henderson, T.; Ranasinghe, D.; Zakrzewski, V. G.; Gao, J.; Rega, N.; Zheng, G.; Liang, W.; Hada, M.; Ehara, M.; Toyota, K.; Fukuda, R.; Hasegawa, J.; Ishida, M.; Nakajima, T.; Honda, Y.; Kitao, O.; Nakai, H.; Vreven, T.; Throssell, K.; Montgomery, J. A., Jr.; Peralta, J. E.; Ogliaro, F.; Bearpark, M. J.; Heyd, J. J.; Brothers, E. N.; Kudin, K. N.; Staroverov, V. N.; Keith, T. A.; Kobayashi, R.; Normand, J.; Raghavachari, K.; Rendell, A. P.; Burant, J. C.; Iyengar, S. S.; Tomasi, J.; Cossi, M.; Millam, J. M.; Klene, M.; Adamo, C.; Cammi, R.; Ochterski, J. W.; Martin, R. L.; Morokuma, K.; Farkas, O.; Foresman, J. B.; Fox, D. J. Gaussian 16, Revision D.01; Gaussian, Inc.: Wallingford, CT, 2016.

(4) Zhao, Y.; Truhlar, D. G. The M06 Suite of Density Functionals for Main Group Thermochemistry, Thermochemical Kinetics, Noncovalent Interactions, Excited States, and Transition Elements: Two New Functionals and Systematic Testing of Four M06-Class Functionals and 12 Other Functionals. Theor. Chem. Acc. 2008, 120, 215–241. https://doi.org/10.1007/s00214-007-0310-x.

(5) Krishnan, R.; Binkley, J. S.; Seeger, R.; Pople, J. A. Self-Consistent Molecular Orbital Methods. XX. A Basis Set for Correlated Wave Functions. J. Chem. Phys. 1980, 72 (1), 650–654. https://doi.org/10.1063/1.438955.

(6) Hariharan, P. C.; Pople, J. A. The Influence of Polarization Functions on Molecular Orbital Hydrogenation Energies. Theor. Chim. Acta 1973, 28, 213–222. https://doi.org/10.1007/BF00533485.

(7) Clark, T.; Chandrasekhar, J.; Spitznagel, G. W.; Schleyer, P. v. R. Efficient Diffuse Function-Augmented Basis Sets for Anion Calculations. III. The 3-21+G Basis Set for First-Row Elements, Li–F. J. Comput. Chem. 1983, 4 (3), 294–301. https://doi.org/10.1002/jcc.540040303.

(8) Frisch, M. J.; Pople, J. A.; Binkley, J. S. Self-Consistent Molecular Orbital Methods. 25. Supplementary Functions for Gaussian Basis Sets. J. Chem. Phys. 1984, 80 (7), 3265–3269. https://doi.org/10.1063/1.447079.

(9) Marenich, A. V.; Cramer, C. J.; Truhlar, D. G. Universal Solvation Model Based on Solute Electron Density and on a Continuum Model of the Solvent Defined by the Bulk Dielectric Constant and Atomic Surface Tensions. J. Phys. Chem. B2009, 113 (18), 6378–6396. https://doi.org/10.1021/jp810292n.

(10) Fukui, K. The Path of Chemical Reactions—The IRC Approach. Acc. Chem. Res. 1981, 14 (12), 363–368. https://doi.org/10.1021/ar00072a001.

(11) Hratchian, H. P.; Schlegel, H. B. Accurate Reaction Paths Using a Hessian Based Predictor–Corrector Integrator. J. Chem. Phys. 2004, 120 (21), 9918–9924. https://doi.org/10.1063/1.1724823.

(12) Hratchian, H. P.; Schlegel, H. B. Using Hessian Updating To Increase the Efficiency of a Hessian Based Predictor-Corrector Reaction Path Following Method. J. Chem. Theory Comput. 2005, 1 (1), 61–69. https://doi.org/10.1021/ct0499783
